# Nanopores with an Engineered Selective Entropic Gate Detect Proteins at Nanomolar Concentration in Complex Biological Sample

**DOI:** 10.1021/jacs.4c17147

**Published:** 2025-04-22

**Authors:** Sabine Straathof, Giovanni Di Muccio, Giovanni Maglia

**Affiliations:** † Groningen Biomolecular Sciences & Biotechnology Institute, University of Groningen, 9747 AG, Groningen, The Netherlands; ‡ New York-Marche Structural Biology Center (NY-MaSBiC), Polytechnic University of Marche, Via Brecce Bianche, 60131 Ancona, Italy; § Department of Life and Environmental Sciences, Polytechnic University of Marche, Via Brecce Bianche, 60131 Ancona, Italy

## Abstract

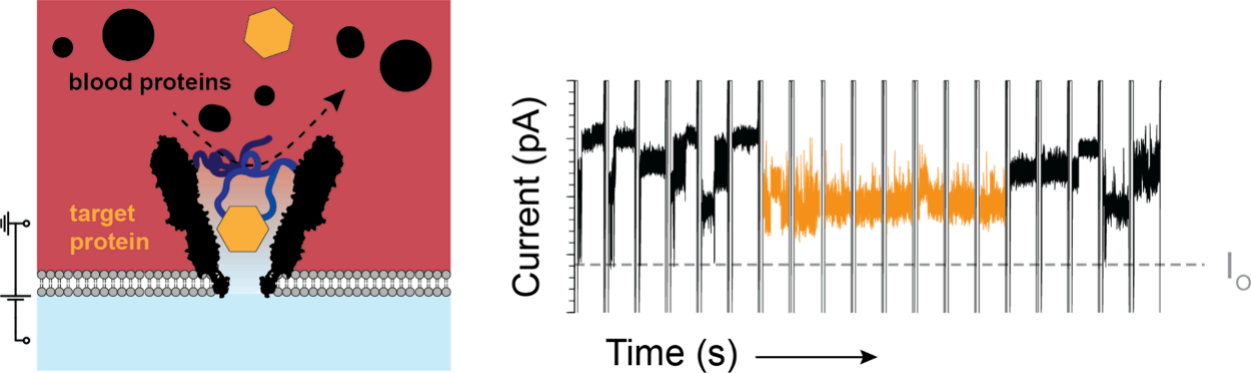

Biological nanopores enable the electrical detection of biomolecules, making them ideal sensors for use in health-monitoring devices. Proteins are widely recognized as biomarkers for various diseases, but they present a unique challenge due to their vast diversity and concentration range in biological samples. Here, inspired by the nuclear pore complex, we incorporated a layer of disordered polypeptides into the biological nanopore YaxAB. This polypeptide mesh formed an entropic gate, significantly reducing the entry of proteins from a highly concentrated mixture, including blood. The introduction of a specific recognition element within the disordered polypeptides allowed targeted proteins to penetrate through the nanopores, where they were recognized by specific current signatures. This biosensing approach allowed for the recognition of nanomolar proteins directly from blood samples without prior sample preparation. This work paves the way for the next generation of nanopore sensors for the real-time detection of proteins in blood.

## Introduction

Personalized healthcare is based on the idea that health and disease can be mapped and tracked at a molecular level, allowing customized intervention, depending on the individual’s needs. In particular, the ensemble of proteins in blood and other bodily fluids changes in composition and abundance during disease^1−9^ and aging.^10−12^ To identify and target these changes, there is a critical need for techniques that can detect specific proteins in real time with high sensitivity directly from blood.

Currently, several techniques are available in proteomics. Mass spectrometry (MS) is the workhorse of proteomic analysis. However, MS also requires complex sample preparation procedures, specialist operators, and large and lab-bound instrumentation, which makes real-time identification of proteins challenging. Immunoassays such as ELISAs^13^ and PEAs^14^ are useful to identify proteins using low-cost assays.^3^ However, they can only identify targets for which an antibody has been developed, need significant adaptation for single-molecule resolution,^15,16^ and cannot be used for continuous protein identification.

Biological nanopores are proteogenic, nanometer-sized, water-filled conduits that can insert into free-standing amphipathic membranes (e.g., lipid bilayers^17^). Upon applying an external potential, the nanopore can capture analytes (e.g., folded proteins^18−24^) from the solution, resulting in a temporary blockade of the current signal. This blockade current is characteristic to the analyte allowing identification, while the frequency of capture relates to its concentration.^24−28^ Importantly, the readout is an electrical current, which makes nanopores amenable for incorporation into portable devices.^29^ Nanopores would therefore be ideal detectors for real-time blood proteins in personalized healthcare devices.

Using large nanopores such as ClyA,^20,25,30^ PlyAB,^19,23^ and YaxAB,^24,27,28^ it has been shown that medically relevant proteins might be identified, in selected cases with single amino acid resolution,^23^ in a label-free and continuous manner. However, because all proteins from the biological sample can enter the nanopore, only highly abundant proteins can now be detected.

Selective protein identification might be acquired using specific protein binders. In an early approach, a PEG-polymer modified with a biotin moiety was attached to an alpha-hemolysin nanopore and streptavidin was identified by changes in the noise induced by the polymer fluctuations.^31^ Using a similar detection mechanism, different antibodies could be measured from the specific gating noise of a biotin-modified OmpG nanopore.^32^ We^18,30^ and others^33,34^ have shown that DNA aptamers, nanobodies or other binders genetically or covalently attached to a nanopore can identify cognate proteins (e.g., SARS-CoV-2 spike). Some of this work showed recognition in the presence of added small amount of background protein samples, serum, or blood. However, the direct detection of proteins from a blood or serum sample has not yet been demonstrated. Furthermore, since the signal originated from the indirect binding of the protein with the binder,^30^ nonspecific interactions lead to false positives, and modifications to the cognate proteins could not be detected.

An ideal nanopore for real-time protein detection in blood would thus require the nanopore to capture target proteins within its interior while preventing the entry of nontargeted proteins. In cells, the nuclear pore complex (NPC) has a selective filter that acts as a gateway between the eukaryotic genome in the nuclear envelope and the cytoplasm.^35,36^ In this work, we have drawn inspiration from NPC and remodeled the YaxAB nanopore with disordered polypeptides to function as an entropic barrier for generic proteins. A specific peptide sequence was then added to the polypeptides to allow the capture and identification of specific, low-abundance target proteins from blood in real time.

## Results

### Design of YaxAB To Detect Streptavidin by Peptide–Protein Interaction

**Figure 1:**
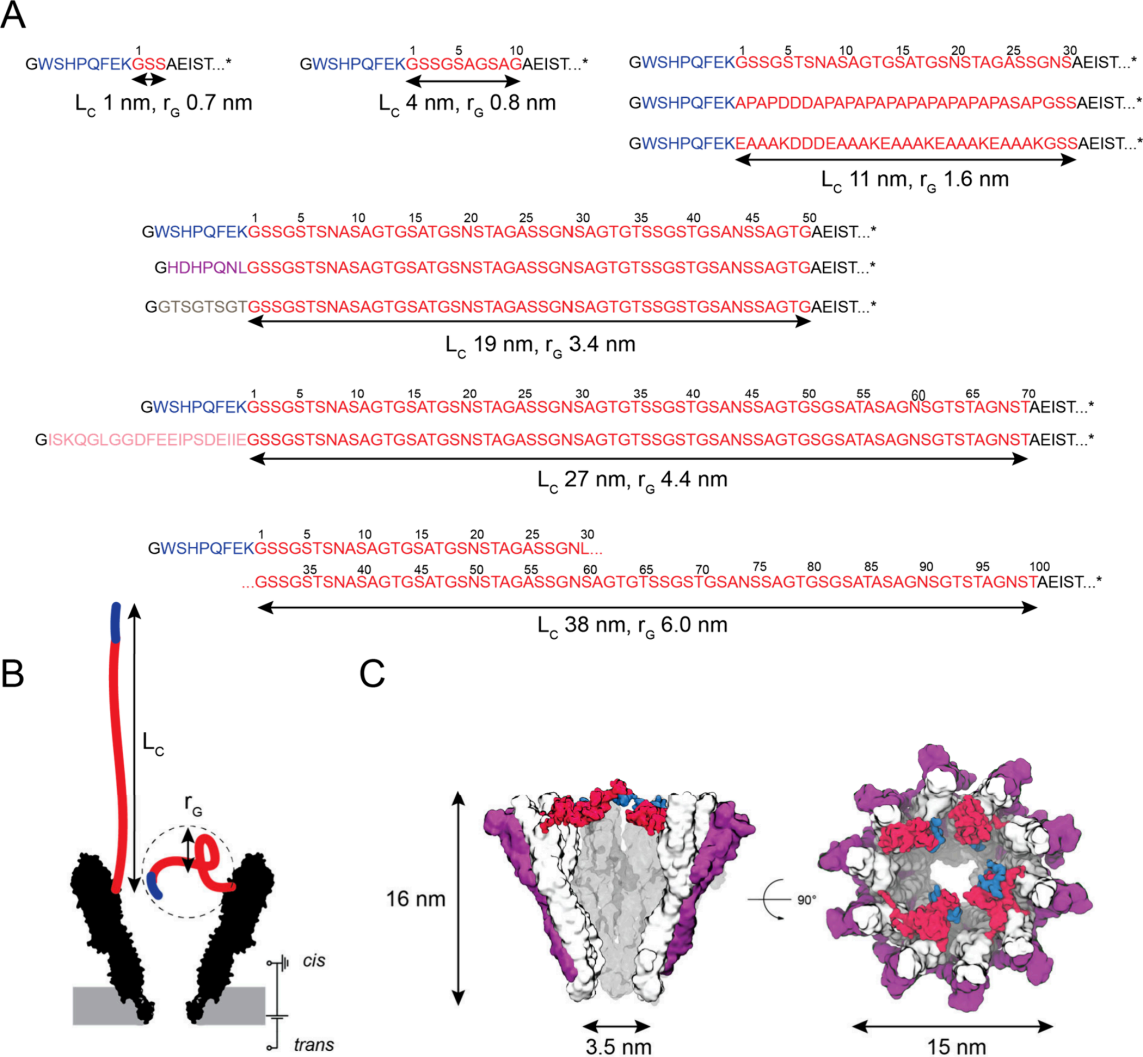
YaxA_Δ40_B_tag-linker_ design. (A) Protein sequences of YaxB component in YaxA_Δ40_B variants used in this work. Sequences are reported from the N- to C-terminus. The strepII-tag is in blue, and the modular linker is in red. Alternative tags, i.e., weak-binding tag, aspecific tag, and IS20-tag are in purple, gray, and pink, respectively. The residues of YaxB are in black, showing only the first 5 residues for simplicity. N-terminal glycine (G, black) is a residue from the TEV-cleavage site (ENLYFQ|G). (Table S2 for full-length sequences.) *L*_C_ indicates the contour length of the linker, and *r*_G_ indicates the radius of gyration as calculated by MD in this work (Table S1). (B) Depiction of the YaxAB nanopore indicating the linker lengths in terms of *L*_C_ (assuming 3.8 Å intra-C_α_ distance), and in terms of *r*_G_. The strepII-tag (in blue, 3.04 nm) is excluded for *L*_C_ in panel (A). (C) Snapshots of YaxA_Δ40_B_strepII-70aa_ after equilibration in MD simulation, showing a side view and a top view. YaxA monomers are shown in purple, YaxB monomers in white with the linkers in red, and the strepII-tag in blue. Dimensions of the didecameric pore are indicated.

YaxAB oligomers form conical nanopores that are a combination of 8–12 YaxA and YaxB dimers, with the didecamer the most prevalent oligomeric shape.^37^ YaxAB pores sometimes have intrinsic gating; therefore, in earlier work, we deleted the first 40 residues of YaxA (YaxA_Δ40_),^27^ which are disordered and point toward the interior of the nanopore, as shown in the Cryo-EM structure.^37^ Under a negative applied potential, YaxAB and YaxA_Δ40_B capture a broad range of protein sizes (12–125 kDa) when added to the *cis* side (Figure 1).^24,27,28^ The N-terminus of YaxB monomers faces the lumen of the YaxAB nanopore. Inspired by the nuclear pore complex (NPC), we introduced disordered polypeptides at these termini to act as entropic barrier, with a peptide-recognition sequence to induce selective transport (Figure 1). As a proof of concept, the recognition element used was the strepII-peptide (WSHPQFEK, 1058 Da, 0*e*, Figure 1A), which binds its cognate protein streptavidin^38^ (SA, 53 kDa) with an affinity (*K*_D_) of ∼70 μM.^39^ The separating linker sequence was chosen to be 3, 10, 30, 50, 70, and 100 amino acids (aa) long. The length of the linkers can be defined as their contour length (*L*_C_, the length of the linker when it is fully extended) and by the radius of gyration (*r*_G_, the average radius of a sphere that defines the volume occupied by the linker; Figure 1B, and Table S1). The sequence of the linker was composed of glycine (G), serine (S), and threonine (T) residues to maximize solubility and flexibility.^40,41^ Alanine (A) and asparagine (N) residues were also introduced to limit repeats and GC content in the genetic construct (Figure 1A, and Tables S2–S5).

### Functionalized YaxAB Detects Target Protein by Caging

Genetically functionalized YaxB variants and YaxA_Δ40_ were successfully oligomerized after purification (Figure S1). All of the YaxA_Δ40_B_strepII-linker_ constructs tested formed functional nanopores. Single-channel conductance was used to identify the different nanopore variants. In this study, we used the nanopores of 2.29 ± 0.23 nS (2.3* nanopores; at −35 mV in 150 mM NaCl), as well as nanopores with a conductance of 1.94 ± 0.09 nS (1.9* nanopores) and 2.63 ± 0.09 nS (2.6* nanopores).

**Figure 2:**
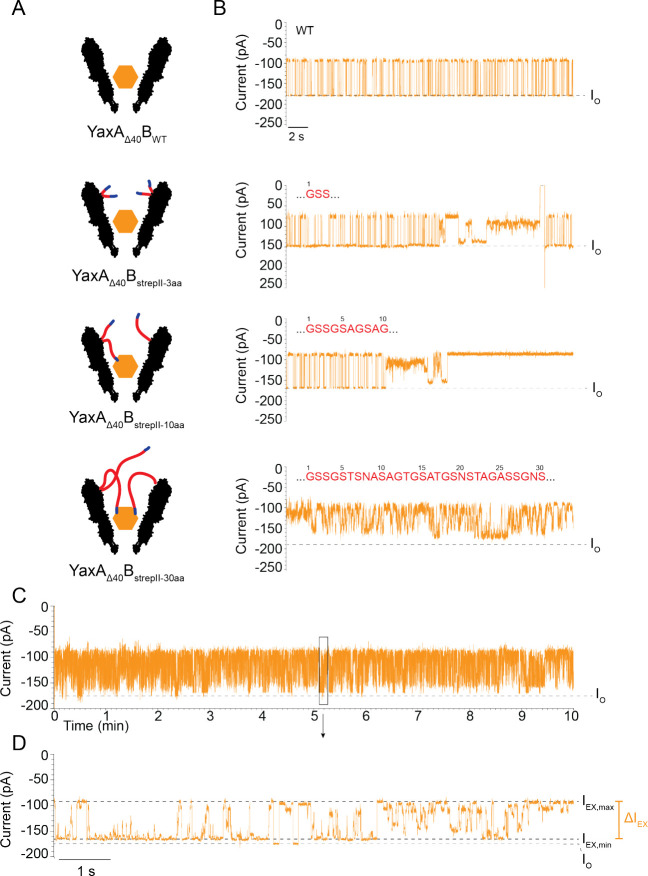
Specific protein detection by functionalized YaxAB. (A) Schematic representation of the YaxA_Δ40_B^2.3^* wild-type (WT) and linker constructs capturing target protein streptavidin (SA). (B) Representative current traces of corresponding nanopores capturing SA. *I*_O_ is indicated by a gray dotted line. The reversible blockades in YaxA_Δ40_B_WT_^2.3*^ showed that SA is often captured and released. YaxA_Δ40_B_strepII-3aa_^2.3*^ and YaxA_Δ40_B_strepII-10aa_^2.3*^ depicted a more-complex behavior, sometimes capturing and releasing SA and sometimes showing noisy SA events. YaxA_Δ40_B_strepII-30aa_^2.3*^ captured SA and did not release it often. (C) Extended 10 min trace of YaxA_Δ40_B_strepII-30aa_^2.3*^ caging SA. (D) Zoom-in on the SA-blockade of YaxA_Δ40_B_strepII-30aa_^2.3*^. Maximum and minimum current blockade (*I*_EX,max_ and *I*_EX,min_, respectively) are indicated by black dotted lines. The bandwidth of the current blockade (Δ*I*_EX_) is indicated in orange. Sublevels can be discerned in the SA blockade, and a brief moment where *I*_O_ is visible before the blockade starts again. SA was added to *cis* at 20 nM. Measurements were conducted at −75 mV, in 150 mM NaCl, 15 mM TrisHCl at pH 7.5, with DPhPC lipids composing the bilayer. Data were recorded at a sampling rate of 50 kHz, and using a 10 kHz Bessel filter. Traces in panel (B) were additionally filtered with 2 kHz low-pass Gaussian filter; traces in panels (C) and (D) were additionally filtered with 500 Hz low-pass Gaussian filter for visualization.

We first tested the effect of SA with the unmodified pore (YaxA_Δ40_B_WT_^2.3*^) by adding SA to the *cis* chamber (20 nM) and applying –75 mV potential. Individual SA proteins entered and escaped the nanopore, as observed by alternating blocked (*I*_B_) and open (*I*_O_) current (Figure 2B). The relative excluded current blockade *I*_EX_ (%) = [(*I*_O_ – *I*_B_)/*I*_O_] × 100 of SA events was 45.4% ± 0.9% and the average dwell time 65.7 ± 14.9 ms (*N* = 4 pores,Table S1).

When introducing the strepII-tag with a linker length of 3 aa or 10 aa (YaxA_Δ40_B_strepII-3aa_^2.3*^ and YaxA_Δ40_B_strepII-10aa_^2.3*^, respectively), the SA blockades did not change significantly, compared to the YaxA_Δ40_B_WT_^2.3*^ (Table S1). However, infrequently, the SA proteins were observed to dwell for longer (e.g., tens to hundreds of seconds, respectively, Figure 2B), although large pore-to-pore variations were observed (Figures S2 and S3).

When the YaxB linker was extended to 30 aa (YaxA_Δ40_B_strepII-30aa_), SA blockades became reproducibly “noisier”, showing several well-defined current states (Figure 2B) with a maximum current blockade (*I*_EX-max_) of 50.9% ± 2.1%. Although the sublevels of individual current blockades did not show reproducible values, the difference between the maximum and the minimum current levels were reproducible [Δ*I*_EX_ (Δ%) = *I*_EX-max_ – *I*_EX-min_] was 42.3 ± 0.6 Δ% (*N* = 5 pores, YaxA_Δ40_B_strepII-30aa_^1.9*/2.3*^, Figure S4). The *I*_O_ was seldomly visible (Figures 2C, 2D, Figure S5), indicating that SA could occasionally exit the nanopore. Compared to the previous systems, however, SA remained inside the nanopore for at least 3 orders of magnitude longer (several minutes), compared to YaxA_Δ40_B_WT_^2.3*^ (Table S1). Hereafter, we refer to this locked state as “caged”. Caging proved robust as the blockade was resistant to flipping of potential (Figure S5), and was visible at low and high voltages (−35 to –100 mV; Figure S6). Upon the addition of biotin – which competes at femtomolar affinity with strepII-peptide for the same binding pocket in SA^42^ – the I_O_ was restored (Figure S6), suggesting the SA caging is indeed caused by the interaction of SA with the strepII-tag attached to the nanopore. Taken together, genetically functionalized YaxA_Δ40_B_strepII-30aa_^1.9*/2.3*^ can specifically detect its target protein and keep the SA caged in the nanopore for minutes.

### Optimization of the Linker Length for Specific Protein Detection

Next, we prepared two constructs with even longer linkers of 50 aa (YaxA_Δ40_B_strepII-50aa_) and 70 aa (YaxA_Δ40_B_strepII-70aa_). In the absence of SA, YaxA_Δ40_B_strepII-50aa_^1.9*/2.3*/2.6*^ and YaxA_Δ40_B_strepII-70aa_^1.9*/2.3*/2.6*^ nanopores showed stable *I*_O_ (Figure S7). In rare instances, a shallow blockade was observed (*I*_EX_ < 10%), which could be reversed by flipping the applied potential to regenerate the *I*_O_ (Figure S7). Such events likely correspond to linkers entering the nanopore. However, the absence of long-lasting gating events suggest that the linkers mostly fluctuate on top of the nanopore, distant from the *trans*-constriction, which is consistent with earlier work on YaxAB.^27^

**Figure 3:**
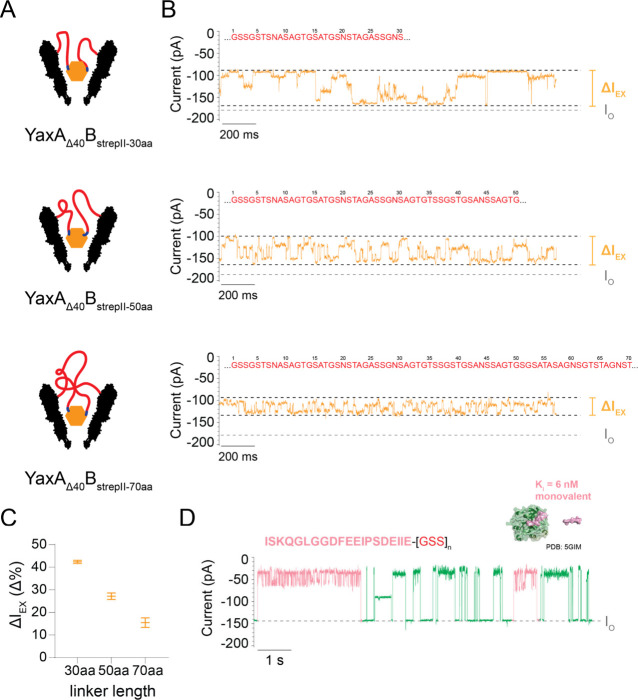
Specific protein capture by YaxAB with longer linkers. (A) Schematic structure of YaxA_Δ40_B_strepII-linker_ constructs, with linker length of 30, 50, and 70 amino acids (aa) caging SA (orange). Linker is in red, and strepII-tag is in blue. (B) Representative current trace of SA blockades measured with the corresponding YaxAB construct. *I*_O_ (gray dotted line) is not visible for these linker constructs, suggesting that the SA molecule is almost permanently caged inside the nanopore. The SA blockade showed multiple levels, where the difference between the deeper and shallower current blockades (Δ*I*_EX_, orange) decreased with increasing linker length. (C) Quantification of Δ*I*_EX_ of multiple replicates, showing that Δ*I*_EX_ decreases as the linker length increases. At least *N* = 3 pores were used for quantification, including pores of 1.9*, 2.3* and 2.6* nS conductance. Error bars represent standard deviation. SA was added to *cis* at 20 nM. (D) Representative current trace of Bovine Thrombin (BT, green) blockades detected by YaxA_Δ40_B_IS20–70aa_^1.9*^ nanopore containing a IS20-tag (ISKQGLGGDFEEIPSDEIIE, C-terminal of avathrin, pink) with monovalent affinity (*K*_i_ = 5.8 ± 0.2 nM (ref (43))) for BT. Occasionally, events with longer-lived sublevels in the blockade level (pink) were observed, consistent with specific binding of BT to the IS20-tag. Structural representation (PDB: 5GIM) of BT (green) and avathrin (pink, structure resolved only for DFEEIPSDEI-sequence (ref (43))). BT was added to *cis* at 50 nM. Measurements were conducted at −75 mV, in 150 mM NaCl and 15 mM TrisHCl at pH 7.5, with DPhPC lipids composing the bilayer. Data were recorded at a sampling rate of 50 kHz, using a 10 kHz Bessel filter. Traces were additionally filtered with 500 Hz low-pass Gaussian filter for visualization. In-house MATLAB script was used to calculate Δ*I*_EX_.

YaxA_Δ40_B_strepII-50aa_^1.9*/2.3*/2.6*^ and YaxA_Δ40_B_strepII-70aa_^1.9*/2.3*/2.6*^ caged SA (20 nM *cis*, −75 mV) for minutes (Figures 3A and 3B, Figures S8 and S9), with an maximum blockade *I*_EX-max_ = 49.1% ± 2.6% and 48.1% ± 2.3%, respectively, *N* > 3 pores; Table S1). Similar to YaxA_Δ40_B_strepII-30aa_^1.9*/2.3*^, the SA blockade remained after flipping of the potential (Figures S8 and S9), and stable across different voltages (−35 mV to −100 mV; Figures S10 and S11). The SA blockade also displayed a multilevel signature, with a Δ*I*_EX_ of 27.1% ± 1.2 Δ% (*N* = 3 pores) for YaxA_Δ40_B_strepII-50aa_^1.9*/2.3*/2.6*^ and Δ*I*_EX_ = 15.4% ± 1.8 Δ% (*N* = 4 pores) for YaxA_Δ40_B_strepII-70aa_^1.9*/2.3*/2.6*^ (Figure 3B, as well as Figures S12 and S13). Comparing the Δ*I*_EX_ for the 30, 50, and 70 aa linkers, the Δ*I*_EX_ decreased with increasing linker length (Figure 3C). Specifically, the maximum *I*_EX_ level remained constant and comparable to that of YaxA_Δ40_B_WT_^2.3*^, whereas the minimum *I*_EX_ level decreased with increasing linker length (Figure S14). Furthermore, whereas the SA multilevel of YaxA_Δ40_B_strepII-30aa_^1.9*/2.3*^ and YaxA_Δ40_B_strepII-50aa_^1.9*/2.3*/2.6*^ occasionally displayed erratic behavior on the 10–20 s time scale (Figures S5 and S8), the YaxA_Δ40_B_strepII-70aa_^1.9*/2.3*/2.6*^ construct displayed a reproducible set of SA fingerprints, even on this macroscale (Figure S9), thereby being the most recognizable fingerprint at any time point. Finally, a construct with 100 aa linkers (YaxA_Δ40_B_strepII-100aa_) could be expressed and oligomerized (Figure S1). SA could be captured by YaxA_Δ40_B_strepII-100aa_^1.9*/2.3*/2.6*^, but the current blockade showed strong pore-to-pore variation (Figures S15 and S16). Taken together, a minimal linker length of 30 aa is required for specific detection of SA by fingerprinting blockades, with 70 aa being an optimum of consistent blockade and longer linker length possibly confounding the specific SA capture.

To test whether proteins with only one peptide-binding site could also be detected, we replaced the strepII-tag for a peptide (ISKQGLGGDFEEIPSDEIIE,^43^ IS20; YaxA_Δ40_B_IS20–70aa_) with high monovalent affinity (*K*_i_ = 5.76 nM^43^) for bovine thrombin (BT, 35 kDa). We measured BT (50 nM to *cis*, −75 mV) with YaxA_Δ40_B_IS20–70aa_ pores with conductance 1.9* nS and 2.3* nS, and found that BT was captured and released as individual blockades (*I*_EX_^1.9^* = 75.9% ± 1.4%, *N* = 4; Figures 3D and Figure S17), suggesting that BT could not be caged. BT blockades in YaxA_Δ40_B_WT_^1.9^* (ref (27)) and YaxA_Δ40_B_strepII-70aa_^2.3*^ have intrinsic, fast-paced blockade noise (Figure S18). BT blockades measured with YaxA_Δ40_B_IS20–70aa_^1.9*^ pores displayed occasionally (∼10%) longer-lived sublevels in the blockade, consistent with specific binding and unbinding of BT to the IS20-tag in the pore (Figure S18). This behavior was predominantly visible for slightly smaller pores of 1.9* nS conductance (Figure S17), because BT dwells longer in smaller YaxAB pores.^27^ Taken together, BT can be specifically detected by its monovalent cognate tag, but due to the limited avidity, the specific multicurrent BT-signal signal is short-lived and infrequent.

### Multivalent Binding Originates Multilevel Fingerprint

**Figure 4:**
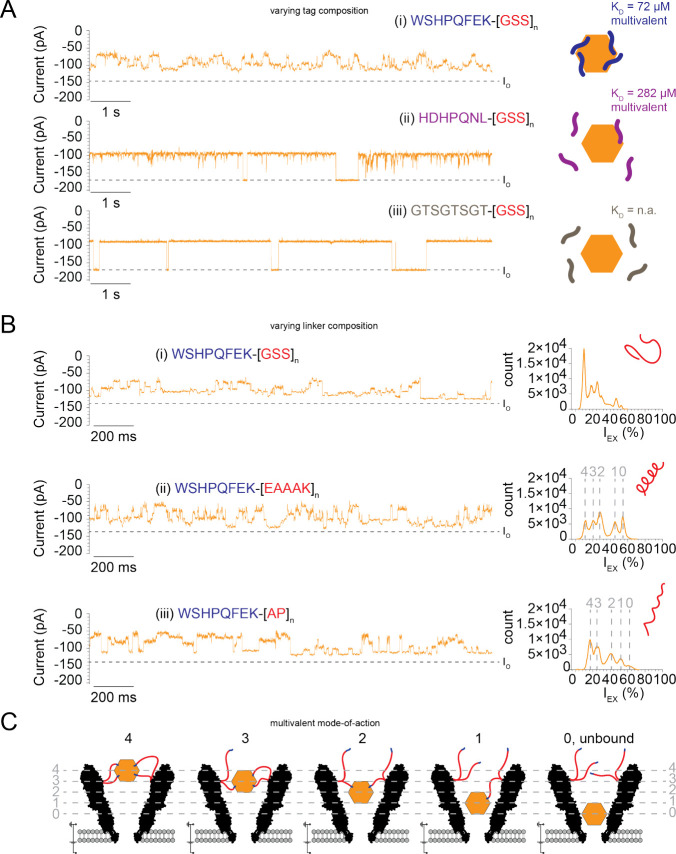
Multilevel fingerprint originates from multivalent binding. (A) SA-blockades (orange) to YaxA_Δ40_B_tag-50aa_^2.3*^ constructs with different linkers and tags: (i) strepII-tag WSHPQFEK (blue) with affinity to SA (*K*_D_) of ∼70 μM;^39^ (ii) weak-tag HDHPQNL (purple, *K*_D_ ≈ 280 μM);^39^ (iii) GTSGTSGT-tag (gray, no affinity). Linkers were 50 aa of length. *I*_O_ is indicated by a black dotted line. On the right side of the trace is the schematic representation of SA and corresponding tag affinity. (B) SA blockades to a range of YaxA_Δ40_B_strepII-linker_^1.9*^ constructs with varying linker composition: (i) WSHPQFEK-[GSS]_*n*_ flexible linker; (ii) WSHPQFEK-[EAAAK]_*n*_ rigid linker; and (iii) WSHPQFEK-[AP]_*n*_ rigid linker. Linkers are 30, 31, and 32 aa of length, respectively. On the right, histogram of 60 s trace of SA-blockade and cartoon representation of corresponding linker. Peaks (gray) likely correspond to the number of tags bound to SA, which are indicated by gray dotted lines. SA was added to *cis* at 20 nM. Measurements were conducted at −75 mV, in 150 mM NaCl, 15 mM TrisHCl pH 7.5, with DPhPC lipids composing the bilayer. Data were recorded at 50 kHz sampling rate, and 10 kHz Bessel filter. Traces were additionally filtered with 500 Hz low-pass Gaussian filter for visualization. (C) Schematic representation of multivalent protein capture. Didecameric YaxAB has 10 available peptides, and the target protein SA has four available peptide-binding pockets. Each level (gray dotted lines) observed in the multilevel blockade likely corresponds to a number or a combination of peptide linkers bound to SA.

To investigate the origin of the SA-multilevel current in YaxA_Δ40_B_strepII-30aa_^1.9*/2.3*^, YaxA_Δ40_B_strepII-50aa_^1.9*/2.3*/2.6*^, and YaxA_Δ40_B_strepII-70aa_^1.9*/2.3*/2.6*^ (Figure 3B), we first replaced the strepII-tag for a lower affinity tag (HDHPQNL,^39^*K*_D_ ≈ 280 μM, YaxA_Δ40_B_weak-50aa_^2.3*/2.6*^, Table S2). The multilevel signature of SA was dramatically reduced (*I*_EX-max_ = 46.5% ± 2.1%, dwell time = 5582 ± 5110 ms; Table S1 and Figure S19), and the *I*_O_ was often visible compared to YaxA_Δ40_B_strepII-50aa_^1.9*/2.3*/2.6*^ (Figure 4A). In a construct with a long polypeptide chain with an aspecific tag (GTSGTSGT, YaxA_Δ40_B_58aa_^2.3*^), the SA-blockades were similar to the YaxA_Δ40_B_WT_^2.3*^ blockades (*I*_EX_ = 47.8% ± 1.8%), but the dwell time increased (1273 ± 95 ms; Figure 4A, Table S1 and Figure S20). This further validates that the SA signature of multilevel and caging is due to specific interactions of the strepII-tags and SA.

We then tested the interaction between SA and the nanopore with linkers composed of a rigid motif,^40,41^ EAAAK and alanine–proline repeats, AP, YaxA_Δ40_B_strepII-31aa-EAAAK_^1.9*/2.3*^ and YaxA_Δ40_B_strepII-32aa-AP_^1.9*/2.3*^, respectively (Table S2). SA caging was similar as observed for YaxA_Δ40_B_strepII-30aa_^1.9*/2.3*^ (Table S1 and Figures S21–S24). Intriguingly, however, the multilevel current signatures resolved to five discrete peaks in an all-point *I*_EX_ histogram (Figure 4B) for most replicates with a rigid linker (Figures S22 and S24). Five sublevels are compatible with multivalent SA caging (Figure 4C). SA is a tetrameric protein with four binding pockets, and the functionalized, didecameric YaxAB nanopores have 10 strepII-tags available for binding. Each level likely reflects a different number or combination of linkers being bound to SA, as shown in Figure 4C, i.e., the deepest level corresponding to unbound SA, and the other levels, respectively, reflecting one, two, three, and four linkers bound simultaneously. Furthermore, when biotin was added to the *trans* side, we observed a gradual reduction of the shallow current blockades (Figure S25), suggesting that, as biotin gradually replaces the strepII-tag, SA can penetrate the nanopore deeper. Indeed, in the presence of biotin, the proteins were observed to exit the pore most often from the deepest level. This interpretation is consistent with the observation of only two levels measured for the monovalent binding of BT with YaxA_Δ40_B_IS20–70aa_^1.9*^ (Figures 3D and S18).

### An Entropic Gate for Specific Protein Detection

**Figure 5:**
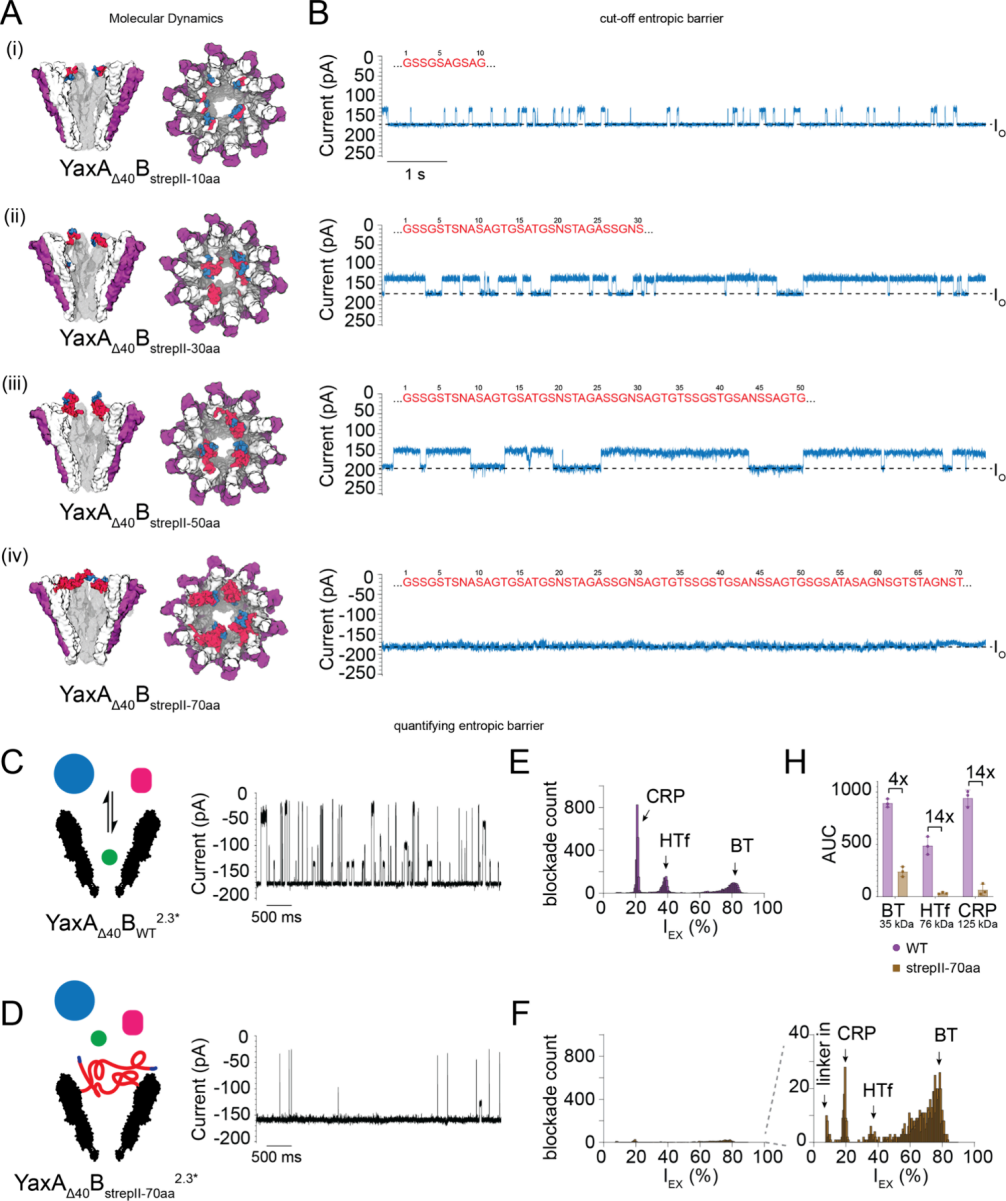
Entropic barrier of functionalized YaxAB constructs. (A) Snapshots of YaxA_Δ40_B_strepII-linker_ constructs after equilibration in MD simulation, showing a side view and a top view. YaxA monomers are shown in purple, YaxB monomers in white with the linkers in red and the strepII-tag in blue. See also Videos S1–S5. (B) Representative current trace (right, 10 s) of corresponding YaxA_Δ40_B^2.3*^ constructs capturing C-reactive protein (CRP, cyan). Linker sequences are shown in red. *I*_O_ indicated by the black dotted line. Shorter linkers of (i) 10, (ii) 30, and (iii) 50 aa depicted typical CRP blockades, whereas the YaxAB with (iv) 70 aa-linkers only depicted *I*_O_ and no protein blockades. CRP was added to *cis* at a concentration of 20 nM. (C) Schematic image and representative trace (5 s) of YaxA_Δ40_B_WT_^2.3*^, and (D) YaxA_Δ40_B_strepII-70aa_^2.3*^ capturing BT, HTf, and CRP, premixed at equal molar ratio (20 nM in *cis* of each). (E) Corresponding histograms (from 519 s traces) of protein blockade events of YaxA_Δ40_B_WT_^2.3*^; and (F) YaxA_Δ40_B_strepII-70aa_^2.3*^. (H) Quantification of nontarget protein capture of WT vs 70aa linker, where the Area Under the Curve (AUC) of each protein peak was calculated and compared. YaxA_Δ40_B_strepII-70aa_^2.3*^ depicts 4-fold reduced protein capture for smaller proteins (BT), and 14-fold reduced capture for larger proteins (HTf, CRP). This reduction of protein capture suggests the linkers form a barrier on top of the YaxAB nanopore. Experiments were executed in triplicate (*N* = 3 pores for each construct). Error bars in panel (H) represent the standard deviation of the mean and were computed with GraphPad Prism 9.5. Data were recorded at –75 mV, and the first 519 s were used for analysis. Quantification was done with in-house MATLAB script, graphs were generated with GraphPad Prism 9.5. All measurements were performed at 150 mM NaCl, 15 mM TrisHCl pH 7.5, with DPhPC lipids composing the bilayer. Data were recorded at a sampling rate of 50 kHz, using a 10 kHz Bessel filter. Traces were filtered with 2 kHz low-pass Gaussian filter for visualization.

Having established that genetically functionalized YaxAB with linkers of 30 to 70 aa can detect a specific target protein, we sought to explore the optimal linker length that would also behave as an entropic barrier to filter out nonspecific proteins. We tested the filtering properties by measuring aspecific C-reactive protein (CRP, 125 kDa) with YaxA_Δ40_B_strepII-10aa_^1.9*/2.3*^, YaxA_Δ40_B_strepII-30aa_^2.3*^, YaxA_Δ40_B_strepII-50aa_^1.9*/2.3*/2.6*^, and YaxA_Δ40_B_strepII-70aa_^1.9*/2.6*^. Functionalized YaxAB nanopores with linkers of up to 50 aa in length showed CRP blockades (20 nM in *cis*, −75 mV; Figures 5 and S26). However, YaxA_Δ40_B_strepII-70aa_^1.9*/2.6*^ showed a much-reduced CRP capture (Figures 5B and S26).

Next, we sought to quantify this reduced level of protein capture for a range of proteins. We measured an equal molar mixture of three proteins of different size–BT (35 kDa), HTf (human transferrin, 76–81 kDa; Figures S27 and S28) and CRP (125 kDa): 1:1:1, 20 nM of each in *cis*. Although YaxA_Δ40_B_WT_^2.3*^ captured each protein with size-dependent blockades,^27^ rewardingly, YaxA_Δ40_B_strepII-70aa_^2.3*^ captured dramatically less proteins (Figures 5C–H, Figures S29 and S30). We compared the number of blockades (area under the curve (AUC) of blockade histogram) of corresponding proteins over an equal amount of time, and we found that YaxA_Δ40_B_strepII-70aa_^2.3*^ captures about 14-fold less for larger proteins HTf and CRP, and about 4-fold less for smaller protein BT (Figure 5H).

To better visualize the different linkers of YaxAB and explore the mechanisms behind protein exclusion, we conducted a large set of all-atom molecular dynamics (MD) simulations. First, a 25-ns implicit solvent MD was conducted for all of the systems reported in Table S1, three replicas per system. Then, for selected systems (nanopores with linkers of 30, 50, 70, and 100 amino acids), we performed an additional 120 ns of explicit solvent MD (Figures S31 and S32, and Supporting Videos S1–S5). All the simulations encompassed the entire nanopore system including the disordered tails extending the sampling (1.8 M atoms for equilibrated states). Despite the low diffusivity of the tails once collapsed into entangled states and the atomistic force fields are still limited in predicting the average properties of disordered protein domains (more refined force fields exist, e.g., 4-point water mode,^44,45^ but would further increase the computational cost), the MD simulations revealed the likely mechanism for the exclusion of proteins by the engineered nanopores.

In almost all the simulations, the linkers interacted with their neighbors, forming a “crown-like” structure, suggesting that the reduced capture might result from smaller effective diameter at the *cis*-entrance of the nanopore (Figure 5A). Additionally, when the linker lengths were longer than 30 aa, the linkers occasionally reached the tails on the opposite side of the pore (Figure S32) creating a polypeptide “mesh” above the entry of the nanopore. The collision with such a mesh, combined with the reduced diameter of the nanopore when adjacent linkers interact, is likely to cause the observed decrease in protein capture by the YaxAB nanopore, i.e., the entropic barrier.

Taken together, the polypeptide linkers in YaxA_Δ40_B_strepII-70aa_ nanopores act as an entropic barrier limiting the entry of aspecific proteins into the nanopore. A theoretical description of the free energy entropic contributions on the capture frequency and a molecular mechanism behind the entropic barrier’s ability to exclude large proteins is given in the supplementary note in the Supporting Information.

### Quantifying SA in Mixed-Protein Solution

Next, we quantified the limit of detection of YaxA_Δ40_B_strepII-70aa_^1.9*^ for SA in a highly crowded background. We prepared a mixed-protein solution that reflects medical reference concentrations of proteins in serum containing serum albumin (BSA, 66 kDa, 40 mg/mL,^46−51^ 602 μM; Figure S33), transferrin (HTf, 76–81 kDa, 2.6 mg/mL,^48,50,52^ 35 μM; Figure S28), as well as thrombin (BT, 35 kDa, 100 nM^53^) and CRP (125 kDa, 100 nM^50,54,55^). DPhPC lipid bilayers cannot tolerate high concentrations of serum or blood,^27,56−58^ with a serum concentration limit of ∼2%.^25^ Hence, we used a mix of DPhPC and PDB_11_PEO_8_ (1:1 w/w, 5 mg/mL, w/v in pentane) in pentane, as described elsewhere.^59^ In hybrid membranes, nanopores showed similar properties as in DPhPC membranes. However, hybrid membranes favored the insertion of smaller 1.9* nanopores.

**Figure 6:**
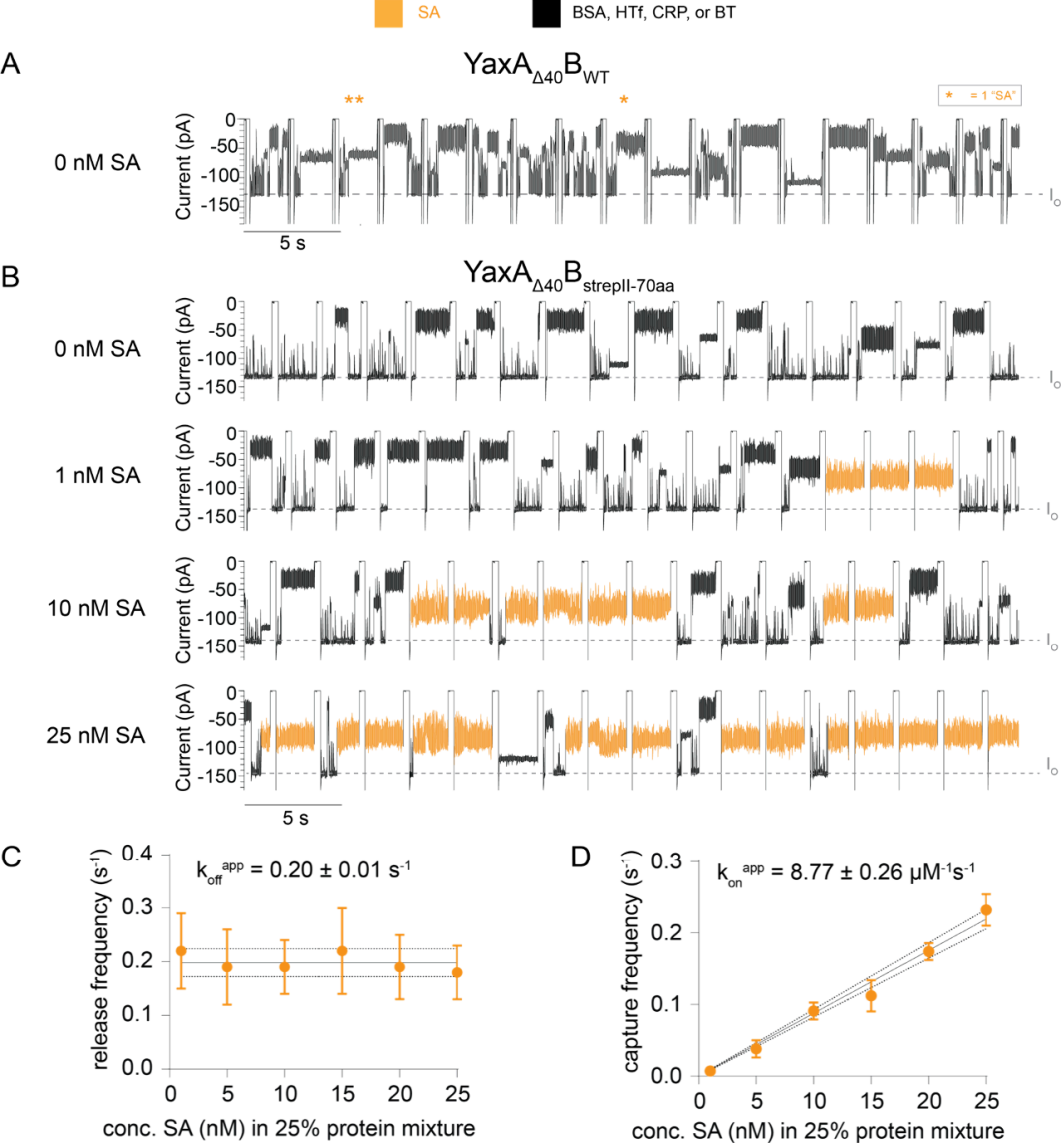
Calibration of Streptavidin (SA) in a 25% Mixed-Protein Solution Detected by YaxA_Δ40_B_strepII-70aa_^1.9*^. (A) Current trace of YaxA_Δ40_B_WT_^1.9*^ detecting 25% mixed protein solution in *cis*. Orange star indicates a false positive “SA” event. *I*_O_ is indicated by the gray dotted line. (B) Representative traces of SA detection by YaxA_Δ40_B_strepII-70aa_^1.9*^ were obtained when titrated in 25% (v/v) mixed protein solution in *cis*. Characteristic SA blockades (orange) can be observed among other proteins. (C) Release frequency of SA in the background of mixed protein solution is stable over SA concentration. The apparent off-rate (*k*_off_^app^) of 0.20 ± 0.01 s^–1^ was computed by GraphPad Prism 9.5, using least-squares regression analysis. (D) Capture frequency of SA in the background of mixed-protein solution correlated with the SA concentration in *cis*. The apparent on-rate (*k*_on_^app^) of 8.77 ± 0.26 μM^–1^ s^–1^ was computed by GraphPad Prism 9.5, using least-squares regression analysis. Dotted lines represent 95% confidence interval (CI); error bars in panels (C) and (D) represent the standard deviations of at least *N* = 3 pores per data point. The mixed protein solution was composed of 602 μM BSA, 35 μM HTf, 100 nM BT, 100 nM CRP (reminiscent to real-life blood serum concentrations^46−55^), premixed before adding to *cis* at a final concentration of 25% (v/v). 80 nM biotin was added to *trans* to increase the off-rate of SA-strepII interaction. Measurements were conducted in 150 mM NaCl, 15 mM TrisHCl pH 7.5, and a PDB_11_PEO_8_:DPhPC (1:1)-hybrid bilayer. Data were recorded at −75 mV in sweeps protocol, at a sampling rate of 50 kHz, using a 10 kHz Bessel filter. Traces were additionally filtered with a 2 kHz low-pass Gaussian filter for visualization.

Addition of this mixed protein solution (25% v/v in *cis*) to YaxA_Δ40_B_WT_^1.9*^ induced many blockades over a wide range of excluded currents corresponding to the proteins entering the nanopore (Figures 6A and S34). Some protein blockades showed *I*_EX_ and dwell time values similar to SA in buffer (Table S1 and Figure S35). In a 25% mixed-protein solution without SA, YaxA_Δ40_B_WT_^1.9*^ depicted “SA” events at 0.06 ± 0.03 s^–1^ (*N* = 4 pores), compared to 0.27 ± 0.11 s^–1^ (*N* = 6 pores) in the presence of 25 nM SA. This suggests that (i) in a 25% mixed-protein solution with 25 nM SA, ∼20% of “SA” events are false positive in YaxA_Δ40_B_WT_^1.9*^, and (ii) YaxA_Δ40_B_WT_^1.9*^ could not detect SA specifically.

When measuring the mixed-protein solution (25% v/v in *cis*) with YaxA_Δ40_B_strepII-70aa_^1.9*^, we observed a much-reduced capture of proteins. BSA, CRP and HTf were barely captured, while smaller BT was occasionally visible (Figure 6B), which was consistent with entropic gate characteristics (Figure 5). Many protein blockades at −75 mV lasted for several seconds (Figure S17), therefore, the potential was regularly flipped using a sweep protocol to eject those from the nanopore (Figure 6B). Next, we titrated SA (1–25 nM) in a background of this mixed protein solution (25% v/v in *cis*) and measured the SA capture by YaxA_Δ40_B_strepII-70aa_^1.9*^ for 10 min (Figure 6B). The SA fingerprint could be identified at concentrations as low as 1 nM (final concentration in *cis*, Figure 6B) by its characteristic multilevel blockade. Since the binding of SA was often permanent, a small amount of biotin was added to *trans* (80 nM final concentration) to increase the SA off-rate from the nanopore (*k*_off_^app^ = 0.20 ± 0.01 s^–1^, Figure 6C), allowing for measuring multiple blockades.

We found that, upon increasing the SA concentration, the number of SA-fingerprint events increased. For each titration point, the capture frequency of SA was calculated by counting the number of sweeps that contained SA events and normalizing for the number of sweeps that did not contain SA (see the Methods section). This capture frequency correlated with the SA concentration in *cis* (Figure 6D), and fitting to a linear regression revealed an apparent on-rate (*k*_on_^app^) of 8.77 ± 0.26 μM^–1^ s^–1^. The apparent binding constant (*K*_D_^app^ = *k*_off_^app^/*k*_on_^app^) was therefore ∼23 nM. This is significantly lower than the reported binding affinity of SA for strepII-peptide (*K*_D_ ≈ 70 μM (ref (39))), reflecting the avidity effect of the multiple binding sites in YaxA_Δ40_B_strepII-70aa_^1.9*^, and despite the presence of biotin in *trans* side of the nanopore, which increased the *k*_off_^app^.

### Direct SA Detection in Blood

Finally, we investigated the biosensing capabilities of YaxA_Δ40_B_strepII-70aa_^1.9*/2.3*^ with the apex of complex samples: blood (Figure S36). Blood contains proteins in all shapes, sizes, and abundancies that relate to the state of an individual’s health throughout every stage of life.^1−12^ Until recently, it was not possible to measure high concentrations (>2%)^25^ of blood (products) with nanopores, due to the instability of lipid bilayers with blood.^28,54−56^ We used a hybrid mix of DPhPC and PDB_11_PEO_8_ (1:1, 5 mg/mL, w/v in pentane) as described elsewhere,^59^ which allowed measuring up to 25% (v/v) dilutions of blood with YaxAB for up to ∼10 min.

**Figure 7:**
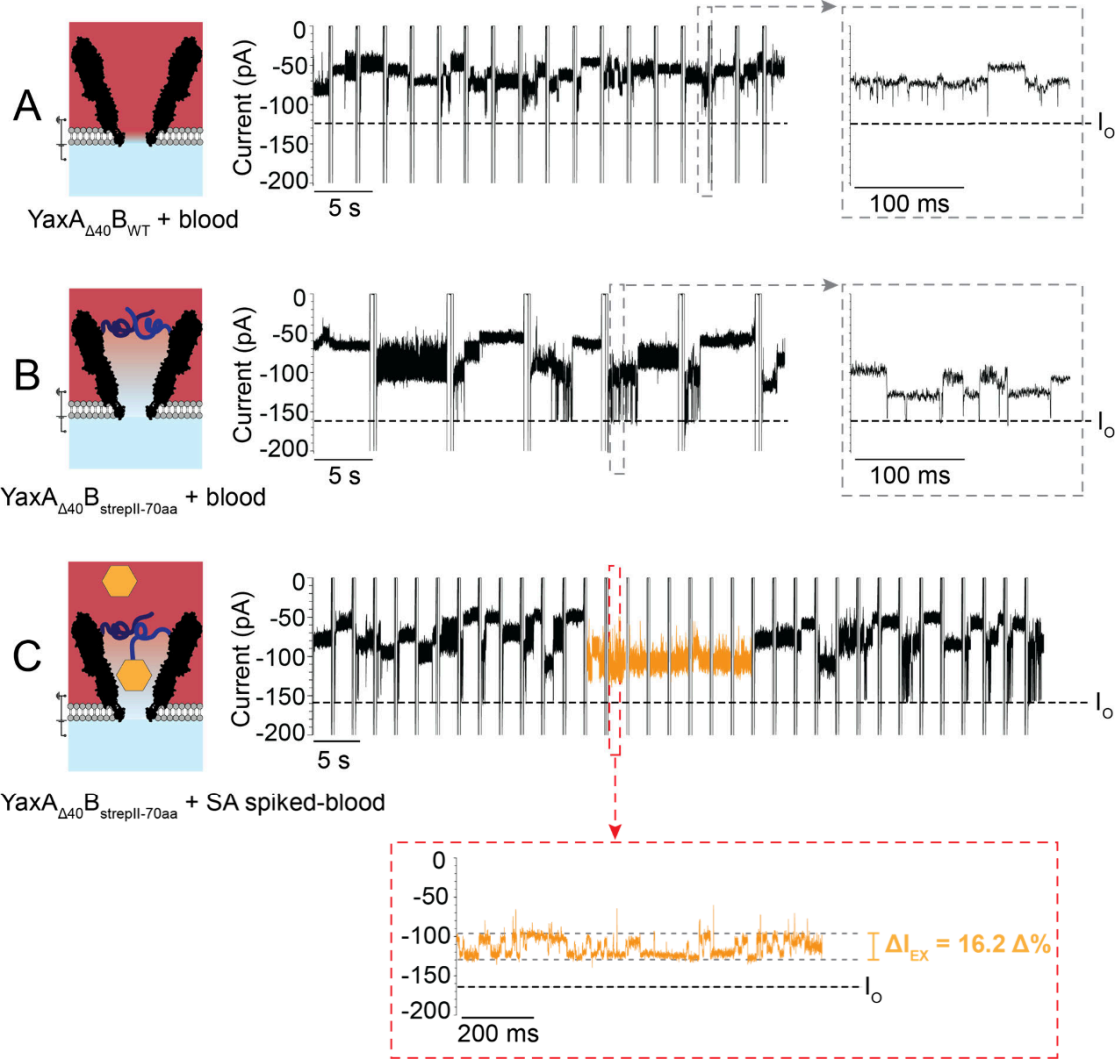
Detecting SA at nanomolar concentration from blood with engineered YaxAB. (A) Schematic image and representative current trace of YaxA_Δ40_B_WT_^1.9*^ detecting 25% (v/v) whole blood. *I*_O_ (black dotted line) is rarely visible, even after zoom-in (gray dotted square). New blockades appear in each sweep showing that the nanopore is not permanently clogged. (B) 25% (v/v) whole blood detected by YaxA_Δ40_B_strepII-70aa_^2.3*^. *I*_O_ is visible, suggesting that fewer proteins enter the nanopore compared to YaxA_Δ40_B_WT_^1.9*^. (C) 10% (w/v) whole blood containing SA (added to the blood sample, 5 nM SA final concentration in *cis*) detected by the YaxA_Δ40_B_strepII-70aa_^2.3*^ nanopore. Characteristic SA blockade (orange) is visible among blood proteins (see Figure S41 for a complete trace). Biotin was added to the *trans* (10 nM final concentration) to increase the off-rate of SA-strepII binding. Measurements were conducted in 150 mM NaCl, 15 mM TrisHCl pH 7.5, and a PDB_11_PEO_8_:DPhPC (1:1)-hybrid bilayer with blood added to *cis* after pore insertion. Data were recorded at −75 mV in sweeps protocol, at a 50 kHz sampling rate, and 10 kHz Bessel filter. Traces were additionally filtered with 2 kHz low-pass Gaussian filter for visualization. (See Figures S37–S48 for full traces and replicates.)

The addition of defibrinated sheep blood (25% v/v) in the *cis* side of YaxA_Δ40_B_WT_^1.9*^ induced many and diverse protein blockades at −75 mV (Figure 7A, and Figure S37). The *I*_O_ was rarely observed, suggesting that YaxA_Δ40_B_WT_^1.9*^ was almost continuously occupied by proteins. Flipping of the potential (+100 mV to −75 mV), the blockades from long-dwelling events could be reversed, indicating that proteins could be removed from the lumen of the nanopore, i.e., blood proteins were not caged. The addition of defibrinated sheep blood (25% v/v, in *cis*) to YaxA_Δ40_B_strepII-70aa_^2.3*^ also induced multiple, diverse current blockades. However, the *I*_O_ could be occasionally observed (Figure 7B), indicating that the 70-aa linkers prevented many proteins from entering the nanopore. Importantly, no protein current blockade in blood was similar to the SA-fingerprint blockade (total 33 recorded minutes over 3 nanopores; Figures S38–S40). Next, we premixed 50 nM SA with defibrinated sheep blood and measured 10% of this solution (v/v) with YaxA_Δ40_B_strepII-70aa_^2.3*^ (5 nM SA final concentration in *cis*, 10 nM biotin in *trans*). Within 10 min of recording, we could detect the SA fingerprint, composed of multilevel and caged signals, three times (Δ*I*_EX_ = 15.4 ± 3.2 Δ%, *n* = 3; Figure 7C, Figure S41). SA at nanomolar concentration could also be detected by slightly smaller YaxA_Δ40_B_strepII-70aa_ pores (e.g., 1.6 nS conductance; Figures S42 and S43) and when the blood supernatant spiked with SA was measured (i.e., cell-free; up to 25% v/v final concentration in *cis*; Figures S44–S48). Therefore, YaxA_Δ40_B_strepII-70aa_^1.9*/2.3*^ nanopores are capable of detecting SA directly in blood without sample preparation, highlighting the potential to be further developed as a blood-protein biosensor in home diagnostics and wearable devices.

## DISCUSSION AND CONCLUSIONS

In this study, we designed a nanopore that could detect a specific protein directly from the blood. Inspired by the nuclear pore complex, we introduced a peptide mesh above and within the nanopore to generate an entropic barrier for generic proteins. After exploring different polypeptide lengths, we showed that a YaxA_Δ40_B construct with a 70-aa polypeptide extension could substantially reduce the entry of a generic protein. The introduction of a binding element to the polypeptide created a nanopore that selectively recognized its cognate protein, while preventing the majority of noncognate proteins from entering the nanopore.

This approach worked with proteins with one or multiple binding sites. However, multivalent detection produced better results, because it showed very efficient trapping and highly unique protein signatures. We expect that nanopores with different binding elements targeting different proteins will allow multiprotein detection within a single nanopore, while using binders with different affinities for the same protein will allow very precise modulation of the nanopore binding affinity and specificity for a single targeted protein. For quantitative measurements, the reversibility of the binding is important. This might be achieved by adding molecules competing for the same binding sites to *trans* compartment, as done here with biotin. Alternatively, especially for multivalent binders, point mutations may be introduced in the nanopore’s binding peptide to reduce affinity for specific epitopes.

Investigation of the polypeptide length and flexibility revealed the mechanism of protein caging and detection. YaxA_Δ40_B nanopores with polypeptide linkers between 30 aa and 70 aa in length depicted highly unique SA blockades. Most likely these linkers are sufficiently long to allow the binding to multiple binding sites simultaneously. Interestingly, rigid polypeptide linkers provided five distinguishable sublevels within SA blockades, most likely reflecting the occupancy of the different binding sites in the tetrameric protein.

Using this approach, we found that YaxA_Δ40_B_strepII-70aa_^1.9*/2.3*^ nanopores could detect protein directly from blood at nanomolar concentrations without a prior sample preparation step. This is remarkable, considering the high protein concentration in blood (∼70 mg/mL)^50^. Previous work showed nanopore detection of proteins in the presence of an added background of small amounts of blood or serum,^27,33^ or the concentration of proteins at very high concentrations (e.g., hemoglobin^23^). However, nanomolar detection of proteins in blood by a nanopore sensor has yet to be reported. Considering a wide range of proteins (35–125 kDa^27^) can penetrate into the YaxAB nanopore lumen, this is a versatile platform to be engineered for sensing a wide range of protein targets. Of note, the lipid–polymer hybrid membrane used in this work allowed measurements in solution containing 25% of blood. However, we found this composition is not stable for tens of minutes. Integration into a working device will most likely require further optimization in the composition of the amphipathic membrane.

Continuous protein detection from blood in a portable device would revolutionize personalized medicine by allowing early diagnostics, by significantly improving therapeutic efficacy as well as improving the cost efficiency^60,61^ of healthcare administration in hospitals and society. This work shows that nanopores can detect nanomolar protein concentrations in a complex environment, providing a first important step in the real-time monitoring of proteins. Many medically relevant proteins, however, are present in blood at femtomolar^62−64^ or attomolar^15,16,65,66^ concentrations. Hence, the next technical challenge is to sample subnanomolar concentrations. We have shown here that one pore can detect low nanomolar protein concentrations within ∼10 min. Thus, arrays of nanopores would be capable of detecting lower concentration, providing that the signal for each targeted protein is unique as observed for SA. Alternatively, if continuous identification is not required, i.e., in home or medical diagnostic devices, simple sample preparation or enrichment steps might allow the detection of subnanomolar concentration of proteins.

## Methods

### Materials

6-Cyclohexylhexyl β-d-maltoside (Cymal-6, CAS No. 228579–27–9), bovine serum albumin (BSA, CAS No. 9048–46–8), bovine thrombin (BT; CAS No. 9002–04–4), C-reactive protein (CRP, AG723), diphytanoyl-*sn*-glycero-3-phosphocholine (DPhPC, CAS No. 207131–40–6), hexadecane 99% (CAS No. 544–76–3), and human transferrin (HTf; CAS #11096–37–0) were ordered from Sigma/Merck.

Defibrinated sheep blood (Catalog No. R54008), DpnI enzyme (Catalog No. ER1701), GeneJET PCR Purification Kit (Catalog No. K0701), GeneJET Plasmid Miniprep Kit (Catalog No. K0503), Low Protein Binding Microcentrifuge Tubes (1.5 mL, Catalog No. 15352617), Phusion U Hot Start polymerase (Catalog No. F555L), and Streptavidin (SA, Catalog No. 21122), were ordered from ThermoFisher Scientific.

TEV-protease (Catalog No. P8112S) and USER-enzyme (Catalog No. M5505L) were ordered from Bioke.

(Poly)butadiene-*b*-ethylene oxide (PDB_11_PEO_8_,^59^ 650-*b*-350 g/mol, P41807C-BdEO) was ordered from Polymer Source, Inc.

d(+)-Biotin (CAS No. 58–85–5) and other chemicals used were ordered from Carl-Roth.

Sequencing was performed by Macrogen and Eurofins. DNA primers were ordered from Integrated DNA Technologies (IDT).

### Cloning of YaxB Variants

YaxA_Δ40_ and YaxB_WT_ were prepared and obtained as described previously.^27^ YaxB variants (Table S2) were prepared by introducing extra amino acids at the N-terminus with USER cloning.^67^ Linker sequences were prepared using a synthetic gBlock (Table S3) and/or using primers compatible with USER cloning (primers are given in Tables S4 and S5). Typically, the constructs were amplified with Phusion U Hot Start polymerase (50 μL final volume; initial denaturation at 98 °C for 30 s, 30 cycles of denaturation at 98 °C for 5 s, annealing at corresponding temperature for 15 s, and extension at 72 °C for 30 s, final extension at 72 °C for 5 min), and purified with GeneJET PCR Purification Kit. The purified PCR products were digested with DpnI to remove template DNA at 37 °C for 1 h. PCR products were ligated together with USER-enzyme (10 μL final volume; 37 °C for 20 min, then 25 °C for 20 min). The ligation mix (5 μL) was then used to transform 100 μL of chemically competent *E. cloni* cells as follows: incubated on ice for 20 min, heat-shocked at 42 °C for exactly 30 s, primary recovery on ice for 2 min; added 700 μL of recovery medium (LB + 1% glucose) and secondary recovery at 37 °C for 1 h before plating out. Transformants were picked next day and grown in LB media (5 mL, 37 °C, 180 rpm, overnight). Plasmids were purified with GeneJET Plasmid Miniprep Kit and sent for sequencing (Macrogen or Eurofins). Correct clones were selected for further protein production.

### Protein Purification and Oligomerization

Protein expression and purification of YaxA_Δ40_ and YaxB monomers, and subsequent oligomerization was performed as described in ref (27) with the following addition. The concentration of the purified monomers was determined with Nanodrop. After purification, the his-tag was cleaved by TEV-protease at 30 °C for 2 h, followed by a 4 °C treatment overnight. Oligomerization and SEC purification were performed the next day.

### Electrical Recordings in Planar Bilayers

An electrophysiology chamber composed of two 500 μL compartments (*cis* and *trans*) separated by a 20 μm PTFE film with a central aperture of ∼100 μm was used for all experiments. To make a bilayer, a drop of hexadecane (4% v/v in pentane) or a drop of hexadecane/silicon oil (1:1, 2%, v/v in pentane) was loaded on an aperture on the *trans*-side of the PTFE film and allowed to evaporate for ∼2 min. Each compartment was then filled with 400 μL of SDEX buffer (150 mM NaCl, 15 mM TrisHCl pH 7.5), and two drops of lipids (DPhPC, 5 mg/mL w/v in pentane) or a mixture of block copolymers and lipids (PDB_11_PEO_8_:DPhPC, 1:1 v/v, 5 mg/mL, w/v in pentane) were added. DPhPC could be used with hexadecane or hexadecane/silicon oil. Hybrid lipid:copolymer mixtures could only be used with hexadecane oil, as the bilayer would be leaky in the presence of silicon oil. Ag/AgCl electrodes were inserted to each compartment: *trans* was the working electrode, *cis* was the ground electrode. By lowering and raising the buffer level in one compartment above the aperture, a bilayer could be formed. A formed bilayer was equilibrated for 5–10 min before pores were added. Prior to use, YaxAB pores from a SEC fraction were diluted 500–100 times in buffer (150 mM NaCl, 50 mM HEPES pH 7.0, 0.05% cymal-6). Then, a small volume (<0.1–0.3 μL) of appropriately diluted YaxAB pores were added to the *cis* chamber. Generally, a pore would be inserted within 10 min. The pore size was determined by reading the current at –35 mV applied potential. The SEC fraction from the center of the elution peak predominantly contained pores of 2.29 ± 0.23 nS (at −35 mV in 150 mM NaCl; previously 80* in ref (27), referred to as 2.3* in this work), and sometimes smaller pores of 1.94 ± 0.09 nS (1.9*) and larger pores of 2.63 ± 0.09 nS (2.6*) conductance (see also ref (27)). In this work, mainly 2.3* nS nanopores were used, unless otherwise specified. Protein (analyte) concentration was determined with a Bradford assay or spectrophotometer absorbance at 280 nm. Analytes were added to the *cis* chamber. Mixed protein solutions were premixed in low-binding tubes before they were added to the *cis* chamber. Experiments were executed at least in triplicate. In this work, only *bona fide*-pores with stable, clean and quiet open pore current (*I*_O_) with the targeted conductance were used, as recorded prior to adding any analyte (blank). *Bona fide*-pores represented ∼20% of the total recordings. Between blood experiments, the chamber was briefly (∼10 min) incubated with 250 mM NaOH to remove all protein residue. Measurements were conducted with a sampling frequency of 50 kHz and a 10 kHz Bessel filter, unless otherwise specified.

### Electrophysiological Data Recording and Analysis

All experimental nanopore data using proteins were recorded under a negative applied potential (−35 mV to −100 mV, *trans*), using an Axopatch 200B patch clamp amplifier connected to a DigiData 1440 A/D converter (Axon Instruments), and using Clampex 10.7 software (Molecular Devices). Current/potential (*I*/*V*) curves were taken from −100 mV to +100 mV at increments of 10 mV. Data recordings were made in gap-free settings or under sweep protocol (+100 mV for 200 ms,–75 mV for 2 s, 10 min total recording). Recordings were analyzed with Clampfit 10.7 software (Molecular Devices). Data were digitally filtered with a Gaussian low-pass filter with 500 or 2000 Hz cutoff prior to analysis. *I*_O_ was determined from the blank with Gaussian fit to all-point histogram with a bin width of 0.5 pA. Protein blockades (*I*_B_), dwell time, and interevent time were detected by the Single-Channel Search function in Event Detection. Event detection was monitored manually. Excluded current percent (*I*_EX_ [%]) was calculated as [((*I*_O_ – *I*_B_)/*I*_O_) × 100%] for all events using an in-house MATLAB script (script #1).

For experiments carried out in buffered solution, the average dwell time (τ_off_) and average interevent time (τ_on_) were calculated by an in-house MATLAB script (script #1), by plotting the respective dwell time or interevent time of at least 150 events as a cumulative histogram (edges: 0–500 ms), to which a standard exponential could be fitted. The release frequency (*f*_R_ = 1/τ_off_ in s^–1^) is equal to the off-rate (*k*_off_, s^–1^), i.e., *k*_off_ = 1/τ_off_. The capture frequency (f_C_ = 1/τ_on_ in s^–1^) was converted to the on-rate (*k*_on_, M^–1^ s^–1^) by *f*_C_ = *k*_on_× [POI], where [POI] is the concentration of protein of interest as added to *cis*. The average on-rate per POI was calculated by least-squares regression using GraphPad Prism software, version 9.5.

### Streptavidin Caging Analysis

In experiments where SA was caged, i.e., the absence of *I*_O_, the data were analyzed as follows. The recording was digitally filtered to 500 Hz cut off and an all-point histogram (bin width of 0.1 pA) of exactly 60 s was exported from Clampfit 10.7. An in-house MATLAB script (script #2) converted the histogram from *I*_B_ to *I*_EX_, and a Gaussian curve was fitted to the outer peaks to determine their mean (μ) *I*_EX_. Fitting was monitored manually.

### Entropic Gate Experiments

Three proteins of known size (CRP, 125 kDa; HTf, 76–81 kDa; BT, 35 kDa) were premixed to equal molar ratio (1:1:1, at 2 μM each, 100 μL final volume) in a low-binding Eppendorf tube. Protein concentration was previously determined with a Bradford assay. Upon pore insertion, a *I*/*V* curve and blank were recorded, after which 4 μL of this protein mixture was added to *cis* (i.e., 20 nM of each protein final concentration), and experiment was recorded at −75 mV for 519 s under gap-free protocol. *I*_EX_ of individual protein blockades was plotted as a histogram, where each protein peak after Gaussian fit was integrated, and the area under the curve (AUC) was calculated, using an in-house MATLAB script (script #1).

### Mixed-Protein Solution Experiments

Mixed-protein solution was composed of 602 μM BSA (40 mg/mL), 35 μM HTf, 100 nM BT, 100 nM CRP, reminiscent of real serum,^46−55^ and premixed in low-binding tube before use. For the SA-calibration curve, after adding 25% (v/v) mixed protein solution to the *cis* chamber, another “blank” was taken. Then, biotin (80 nM final concentration) was added to *trans*, and SA was titrated to *cis* and recorded in a sweep protocol for 10 min for each titration step. For experiments with YaxA_Δ40_B_WT_^1.9*^, an event was annotated as “SA” if both its parameters *I*_EX_ and dwell time fell within μ ± 2σ boundaries of the SA-signature in 1.9*-pores (see Table S1). For experiments with YaxA_Δ40_B_strepII-70aa_^1.9*^, the SA events were identified manually, including start and end times as well as the number of sweeps that the SA event occupied. To calculate the capture frequency of SA events for a given concentration, the number of SA events (*n*) was divided over the total recording time excluding SA time, i.e., eq 1a for YaxA_Δ40_B_WT_^1.9*^ and eq 1b for YaxA_Δ40_B_strepII-70aa_^1.9*^, with the measuring time per sweep being 2 s for all recordings.
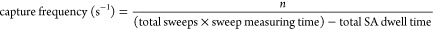
1a
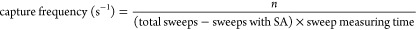
1b

The apparent on rate (*k*_on_^app^) could then be calculated with GraphPad Prism 9.5, using a Straight Line, Least Squares fit, constraining the *Y*-intercept to 0.

The average dwell time of SA at each concentration was calculated by log-transforming the dwell times and taking the average of the log-transformed dwell times, followed by back-transformation. See eq 2, where μ_dwell_ is the average dwell time (in s), and *n* is the number of SA events. (Note: direct exponential fitting was not reliable for few events, in case of low concentration titration points.)
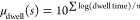
2

The release frequency [*f*_R_ (s^–1^) = 1/μ_dwell_] is equal to the off-rate (*k*_off_, s^–1^), i.e., *k*_off_ = 1/μ_dwell_. The apparent off-rate (*k*_off_^app^) could be calculated with GraphPad Prism 9.5 using Straight Line, Least-Squares Fit, constraining the slope to 0.

### Blood Experiments

Blood was spiked with SA by premixing defibrinated sheep blood and SA (50 nM final concentration) in a low-binding tube, mixed by pipetting and inverting, and stored on ice or at 4 °C until use. Alternatively, after mixing, the spiked blood was shortly centrifuged (1 min, 10 000*g*) to pellet cells, and the blood supernatant with SA was further used. In experiments involving blood or a mixed protein solution, a hybrid polymer/lipid mix of PDB_11_PEO_8_:DPhPC (1:1, 5 mg/mL, w/v in pentane) was used to compose the bilayer. Pores were allowed to insert in the presence of SDEX buffer. Upon insertion, an *I*/*V*-curve and blank were recorded. Then, the buffer in the *cis* compartment was replaced, e.g., for 25% (v/v) blood, i.e., and 100 μL of buffer was replaced with 100 μL of defibrinated sheep blood (400 μL final volume) and mixed well. All blood and mixed-protein solution experiments were recorded in sweeps protocol.

### Molecular Modeling and MD Simulations

#### Structure Initialization

For each YaxB variant listed in Table S2, the sequence to be modeled began immediately after the TEV-cleaved region and was truncated to include all amino acids up to the end of the linker region, along with an additional 34 amino acids from the YaxB_WT_ pore (ending with the sequence “...LAQF”). These sequences were then input into AlphaFold2Multimer^68,69^ via the ColabFold^70^ platform for protein structure prediction. Template-based modeling was performed with two recycling steps and one model generation per sequence. As anticipated, the linker sequences were predicted with low predicted Local Distance Difference Test (pLDDT) scores (around 50) and high Predicted Aligned Error (PAE) values (>20), indicating the absence of a defined fold in these regions. In contrast, the 34 amino acids from the YaxB_WT_ nanopore were correctly modeled as an α helix. Each modeled N-terminal linker tail was aligned to its respective YaxB chain within a complete YaxA_Δ40_B_WT_ heterodecamer complex, which was previously^27^ modeled using the SWISSMODEL^71^ server, resulting in 10 alignments in total. To prevent clashes between the modeled tails and the rest of the structure, each tail was steered outward along the pore axis to its maximum length. This steering process, with a force constant of *k* = 0.01 kcal/(mol Å̊^2^), was conducted while keeping the first 34 Cα atoms of YaxB_WT_ (starting sequence AEIS) fixed. After steering, the modeled tails were merged with the complete YaxA_Δ40_B_WT_ pore structure using the VMD software^72^ (see Figure S31).

#### Implicit Solvent Molecular Dynamics Simulations

Starting from the generated structures, molecular dynamics (MD) simulations were conducted using NAMD 2.14^73^ using the Generalized Born Implicit Solvent (GBIS) model.^74−76^ This method was selected for its computational efficiency in modeling large systems, such as the one studied here, which would contain over 4 million atoms in an all-atom explicit solvent model in the initial configurations. The simulations were performed at a constant temperature of 303.15 K, controlled via Langevin dynamics with a damping coefficient of 1 ps^–1^. The GBIS model used an ion concentration of 0.15 M and a solvent dielectric constant of 78.5. Nonbonded interactions were managed using a switch distance of 15 Å, a cutoff of 16 Å, and a pairlist distance of 18 Å. Hydrophobic effects were calculated by using the solvent-accessible surface area (SASA) model with a surface tension of 0.006 kcal/mol/Å^2^. The integration time step was set to 1 fs, with nonbonded interactions computed every 2 steps and full electrostatics every 4 steps.

#### Explicit Solvent Molecular Dynamics Simulations

After the linkers reached a compact steady state, each equilibrated pore was embedded into a POPC lipid membrane of 26 nm × 26 nm, using VMD Membrane Plugin, as described elsewhere.^82^ Each system was then solvated with TIP3P water molecules and ionized with a 0.15 M NaCl solution using GROMACS standard tools (pdb 2gmx, solvate, and genion). The YaxAB backbone was constrained by excluding the modeled tails. We used the CHARMM36 force field (release July 2022) for protein, lipids, water (TIP3P) and ions. Every system was then re-equilibrated using GROMACS software, by performing 10 000 steps of steepest descent energy minimization, followed by ∼6 ns of NPT equilibration, until the volume reached a steady-state value. Then, a 120 ns NVT production run was performed, with a time step of 2 fs (fs). The integration algorithm used was leapfrog, saving the coordinates every 100 ps. The Verlet cutoff scheme was employed with a 1.2 nm cutoff for both electrostatic interactions (using the Particle-Mesh Ewald method) and van der Waals interactions. A force-switching method was applied for van der Waals forces starting at 1.0 nm and switching off at 1.2 nm. Temperature control was managed using the V-rescale thermostat with a reference temperature of 305 K and a coupling constant of 0.1 ps for the entire system.

#### Note on the Force Field

Recently, promising experimentally driven coarse-grained models have been developed^77−79^ to accelerate simulations of disordered regions of complex systems, like the nuclear pore complex (NPC). These models, which simplify the system by representing each amino acid as a single bead, account for electrostatic and hydrophobic interactions through the modified Coulomb laws. These methods have been applied to study the conformation of FG-nups and the role of these interactions in NPC function and transport.^77,80,81^ However, while this approach allows for modeling the full geometry of the NPC, it sacrifices some detail at the amino acid level. In our study, since the system is smaller than the full NPC, we preferred to keep atomistic details at a single amino acid level.

## Data Availability

Data and corresponding analysis generated in this study have been deposited in the Zenodo database under DOI: 10.5281/zenodo.13132666.

## Supporting Information Available

Tables containing the full sequences of proteins, synthetic gBlock, DNA primers, PCR scheme; protein purifications; replicate measurements of electrophysiology experiments; SDS-PAGE of proteins tested, voltage- and concentration-dependent capture of protein; image of blood-filled electrophysiology chamber; videos of the MD simulations (PDF)
Molecular dynamics (MD) simulations for 25 ns of implicit solvent (AVI)
Molecular dynamics (MD) simulations for 120 ns of implicit solvent (30 amino acids) (AVI)
Molecular dynamics (MD) simulations for 120 ns of implicit solvent (50 amino acids) (AVI)
Molecular dynamics (MD) simulations for 120 ns of implicit solvent (70 amino acids) (AVI)
Molecular dynamics (MD) simulations for 120 ns of implicit solvent (100 amino acids) (AVI)

## Supplementary Material

ja4c17147_si_001.pdf

ja4c17147_si_002.avi

ja4c17147_si_003.avi

ja4c17147_si_004.avi

ja4c17147_si_005.avi

ja4c17147_si_006.avi
