# Supplementary material for: Nanopores with an Engineered Selective Entropic Gate Detect Proteins at Nanomolar Concentration in Complex Biological Sample
Source: J Am Chem Soc. 2025 Apr 22;147(18):15050–65. doi: 10.1021/jacs.4c17147 (PMC12063177; doi:10.1021/jacs.4c17147)
Supplement: Supplementary file 1 — ja4c17147_si_001.pdf [file ja4c17147_si_001.pdf]

Supporting Information

Nanopores with an engineered selective entropic gate detect proteins at nanomolar concentration in complex biological sample

Sabine Straathof[1], Giovanni Di Muccio[2], Giovanni Maglia[1*]

[1]*Groningen Biomolecular Sciences & Biotechnology Institute, University of Groningen, 9747 AG Groningen, The Netherlands*.

[2]*New York-Marche Structural Biology Center (NY-MaSBiC), Polytechnic University of Marche, Via Brecce Bianche, 60131 Ancona, Italy*

[3]*Department of Life and Environmental Sciences, Polytechnic University of Marche, Via Brecce Bianche, 60131 Ancona, Italy*

* Email: giovanni.maglia@rug.nl

Table of Content

Supporting Tables

Supporting Table 1: SA capture characteristics of $YaxA_{\Delta40}B$ linker constructs. 4
Supporting Table 2: Protein sequences used in this work. 4
Supporting Table 3: Synthetic gene sequence of flexible linker in this work. 6
Supporting Table 4: Primers used in this work. 6

Supporting Figures

Supporting Figure 1: YaxAB oligomer variant purifications on Superose 6-FPLC used in this work. 8
Supporting Figure 2: $YaxA_{\Delta40}B_{strepII\text{-}3aa}{}^{2.3*}$ (3 aa) capturing SA. 9
Supporting Figure 3: $YaxA_{\Delta40}B_{strepII\text{-}10aa}{}^{2.3*}$ (10 aa) capturing SA. 10
Supporting Figure 4: $YaxA_{\Delta40}B_{strepII\text{-}30aa}{}^{1.9*/2.3*}$ (30 aa) caging SA. 11
Supporting Figure 5: Macroscale SA caging by $YaxA_{\Delta40}B_{strepII\text{-}30aa}{}^{1.9*/2.3*}$ (30aa). 12
Supporting Figure 6: Voltage dependence of SA-blockades in $YaxA_{\Delta40}B_{strepII\text{-}30aa}{}^{1.9*}$ (30aa) and biotin release. 13
Supporting Figure 7: Linker capture and effect of the amphipathic membrane on $YaxA_{\Delta40}B$ recordings. 14
Supporting Figure 8: Macroscale SA caging by $YaxA_{\Delta40}B_{strepII\text{-}50aa}{}^{1.9*/2.3*/2.6*}$ (50aa). 15
Supporting Figure 9: Macroscale SA caging by $YaxA_{\Delta40}B_{strepII\text{-}70aa}{}^{1.9*/2.3*/2.6*}$ (70 aa). 16
Supporting Figure 10: Voltage dependence of SA-blockades in $YaxA_{\Delta40}B_{strepII\text{-}50aa}{}^{1.9*}$ (50aa) and biotin release. 17
Supporting Figure 11: Voltage dependence of SA-blockades in $YaxA_{\Delta40}B_{strepII\text{-}70aa}{}^{2.3*}$ (70 aa) and biotin release. 18
Supporting Figure 12: $YaxA_{\Delta40}B_{strepII\text{-}50aa}{}^{1.9*/2.3*/2.6*}$ (50aa) caging of SA. 19
Supporting Figure 13: $YaxA_{\Delta40}B_{strepII\text{-}70aa}{}^{1.9*/2.3*/2.6*}$ (70 aa) caging SA. 20
Supporting Figure 14: Length dependence of I_{EX}-values of the multilevel SA-signal of $YaxA_{\Delta40}B^{1.9*/2.3*/2.6*}$ linker constructs. 21
Supporting Figure 15: $YaxA_{\Delta40}B_{strepII\text{-}100aa}{}^{1.9*/2.3*/2.6*}$ capturing SA. 22
Supporting Figure 16: I/V curves of $YaxA_{\Delta40}B_{strepII\text{-}linker}$ constructs. 23
Supporting Figure 17: BT capture by $YaxA_{\Delta40}B_{IS20\text{-}70aa}$ 1.9* and 2.3* nS pores. 24
Supporting Figure 18: BT capture by $YaxA_{\Delta40}B_{IS20\text{-}70aa}{}^{1.9*}$ and control constructs. 25
Supporting Figure 19: $YaxA_{\Delta40}B_{weak\text{-}50aa}{}^{2.3*/2.6*}$ capturing SA. 26
Supporting Figure 20: $YaxA_{\Delta40}B_{58aa}{}^{2.3*}$ capturing SA. 27
Supporting Figure 21: Macroscale SA caging by $YaxA_{\Delta40}B_{strepII\text{-}EAAAK}{}^{1.9*/2.3*}$. 28
Supporting Figure 22: $YaxA_{\Delta40}B_{strepII\text{-}EAAAK}{}^{1.9*/2.3*}$ caging SA. 29
Supporting Figure 23: Macroscale SA caging by $YaxA_{\Delta40}B_{strepII\text{-}AP}{}^{1.9*/2.3*}$. 30
Supporting Figure 24: $YaxA_{\Delta40}B_{strepII\text{-}AP}{}^{1.9*/2.3*}$ caging SA. 31
Supporting Figure 25: Biotin-induced escape of SA from $YaxA_{\Delta40}B_{strepII\text{-}EAAAK}{}^{2.3*}$ nanopores. 32
Supporting Figure 26: CRP capture by $YaxA_{\Delta40}B_{strepII\text{-}linker}{}^{1.9*/2.3*/2.6*}$ nanopores. 33
Supporting Figure 27: Characterization of Human Transferrin (HTf) by $YaxA_{\Delta40}B_{WT}{}^{2.3*}$ nanopores. 34
Supporting Figure 28: Human Transferrin (76-81 kDa) on 12% SDS-PAGE gel. 35
Supporting Figure 29: Aspecific protein capture by $YaxA_{\Delta40}B_{WT}{}^{2.3*}$. 36
Supporting Figure 30: Premixed protein sample capture by $YaxA_{\Delta40}B_{strepII\text{-}70aa}{}^{2.3*}$ linker construct (N=3 replicates). 37
Supporting Figure 31: Implicit-solvent MD simulations of YaxAB linker compaction. 38
Supporting Figure 32: All-atom MD simulations, explicit solvent. 39
Supporting Figure 33: Bovine Serum Albumin (BSA) on 12% SDS-PAGE gel. 40
Supporting Figure 34: $YaxA_{\Delta40}B_{WT}{}^{1.9*}$ detecting mixed protein solution. 41

Supporting Figure 35: YaxA$_{Δ40}$B$_{WT}$$^{1.9*}$ detecting 25% mixed protein solution with 0 and 25 nM SA. 42
Supporting Figure 36: 25% (v/v) blood experiments. 44
Supporting Figure 37: Blood proteins detected by YaxA$_{Δ40}$B$_{WT}$$^{1.9*}$. 45
Supporting Figure 38: Blood proteins detected by YaxA$_{Δ40}$B$_{strepII\text{-}70aa}$$^{1.9*}$. 46
Supporting Figure 39: Blood proteins detected by YaxA$_{Δ40}$B$_{strepII\text{-}70aa}$$^{1.9*}$. 47
Supporting Figure 40: Blood proteins detected by YaxA$_{Δ40}$B$_{strepII\text{-}70aa}$$^{1.9*}$. 48
Supporting Figure 41: Nanomolar SA detection in blood by YaxA$_{Δ40}$B$_{strepII\text{-}70aa}$$^{2.3*}$. 49
Supporting Figure 42: YaxA$_{Δ40}$B$_{strepII\text{-}70aa}$$^{1.6*}$ detecting 25% whole blood and SA. 50
Supporting Figure 43: YaxA$_{Δ40}$B$_{strepII\text{-}70aa}$$^{1.6*}$ detecting 25% whole blood and SA. 51
Supporting Figure 44: YaxA$_{Δ40}$B$_{strepII\text{-}70aa}$$^{1.9*}$ detecting 10% SA-spiked whole blood. 52
Supporting Figure 45: YaxA$_{Δ40}$B$_{strepII\text{-}70aa}$$^{1.9*}$ detecting 10% SA-spiked blood supernatant. 53
Supporting Figure 46: YaxA$_{Δ40}$B$_{strepII\text{-}70aa}$$^{1.9*}$ detecting 10% SA-spiked blood supernatant. 54
Supporting Figure 47: YaxA$_{Δ40}$B$_{strepII\text{-}70aa}$$^{1.9*}$ detecting 25% SA-spiked blood supernatant. 55
Supporting Figure 48: YaxA$_{Δ40}$B$_{strepII\text{-}70aa}$$^{1.9*}$ detecting 25% blood supernatant supplemented with SA. 56
Supporting Figure 49: Schematic representation of the capture process in nanopores with and without an entropic gate. 57
Supporting Figure 50: a) Calculated free energy 64
Supporting Figure 51: Free Energy Landscapes 65

Supporting Table 1: SA capture characteristics of YaxA$_{\Delta40}$B linker constructs. Mutant names refer to the YaxB functionalization. 20 nM SA was added to *cis*. Error bars represent standard deviation. Measurements were conducted at –75 mV, at 150 mM NaCl, 15 mM TrisHCl pH 7.5, with DPhPC lipids composing the bilayer. Data were recorded at 50 kHz sampling rate, and 10 kHz Bessel filter.

Mutant name	Linker length			Current					Dwell time
	aa	L_C (nm)	r_G (nm)	I_{EX} (%)	$I_{EX\text{-}max}$ (%)	$I_{EX\text{-}min}$ (%)	ΔI_{EX} (Δ%)	N	τ (ms)
**WT-1.9***	NA	NA	NA	53.5 ± 1.1	NA	NA	NA	3	10^(1.8±0.4)
**WT-2.3***	NA	NA	NA	47.0 ± 0.9	NA	NA	NA	4	10^(1.7±0.5)
**WT-2.6***	NA	NA	NA	48.0 ± 0.6	NA	NA	NA	3	10^(1.8±0.4)
**3aa**	3	~1.1	0.65	50.1 ± 5.8	NA	NA	NA	5	74.0 ± 32.1
**10aa**	10	~3.8	0.81	47.0 ± 0.4	NA	NA	NA	3	96.4 ± 59.1
**30aa**	30	~11.4	1.58	-	50.9 ± 2.1	8.6 ± 1.8	42.3 ± 0.6	5	>mins
**31aa-EAAAK**	31		1.64	-	49.2 ± 5.5	9.2 ± 2.3	40.1 ± 4.5	6	>mins
**32aa-AP**	32		1.86	-	45.7 ± 11.0	10.9 ± 4.0	34.8 ± 7.2	4	>mins
**50aa**	50	~19.0	3.38	-	49.1 ± 2.6	22.0 ± 1.5	27.1 ± 1.2	3	>mins
**50aa-weak tag**	50		3.70	-	46.5 ± 2.1	ND	ND	3	5582 ± 5110
**50aa-no tag**	50		4.13	47.8 ± 1.8	NA	NA	NA	3	1273 ± 95
**70aa**	70	~26.6	4.41	-	48.1 ± 2.3	32.7 ± 3.3	15.4 ± 1.8	4	>mins
**100aa**	100	~38	5.97	-	ND	ND	ND	6	ND

Supporting Table 2: Protein sequences used in this work. YaxA$_{\Delta40}$ and YaxB$_{WT}$ are the same as in ref.[1] TEV-cleavage site is underlined. **Tags (strepII and weak[2]) are in bold. IS20-tag[3] is in pink.** Linker sequence is in red.

YaxA$_{\Delta40}$<br>MHHHHHHENLYFQSSGGIFTKEDLINLKLYVRKGLSLPTRQDEVEAYLGYKKIDVAGLEPK<br>DIKLLFDEIHNHALNWNDVEQAVLQQSLDLDIAAKNIISTGNEIINLINQMPITLRVKTLLGDIT<br>DKQLENITYESADHEVASALKDILDDMKGDINRHQTTTENVRKKVSDYRITLTGGELSSGD<br>KVNGLEPQVKTKYDLMEKSNMRKSIKELDEKIKEKRQRIEQLKKDYDKFVGLSFTGAIGGII<br>AMAITGGIFGAKAENARKEKNALISEVAELESKVSSQRALQTALEALSLSFSDIGIRMVDAE<br>SALNHLDFMWLSVLNQITESQIQFAMINNALRLTSFVNKFQQVITPWQSVGDSARQLVDIF<br>DEAIKEYKKVYG*
YaxB$_{WT}$<br>MSYYHHHHHHDYDIPTTENLYFQGAEISTFPHSGLSYPDINFKIFSQGVKNISHLAQFKTT<br>GVEVLQEKALRVSLYSQRLDVIVRESLSSLQVKLENTLALTYFTTLEEIDEALISQDIDEESK<br>SEMRKERINIIKNLSNDITQLKQLFIEKTELLDKSSSDLHNVVIEGTDKVLQAEQLRQKQLT<br>EDIATKELERKEIEKKRDKIIEALDVIREHNLVDAFKDLIPTGENLSELDLAKPEIELLKQSLEI<br>TKKLLGQFSEGLKYIDLTDARKKLDNQIDTASTRLTELNRQLEQSEKLIAGVNAIIKIDQEKS<br>AVVVEAEKLSRAWHIFIHEITALQGTSLNEVELSKPLIKQQIYLESLIKQLI*
YaxB$_{strepII\text{-}3aa}$<br>MSYYHHHHHHDYDIPTTENLYFQG**WSHPQFEK**GSSAEISTFPHSGLSYPDINFKIFSQGV<br>KNISHLAQFKTTGVEVLQEKALRVSLYSQRLDVIVRESLSSLQVKLENTLALTYFTTLEEIDE<br>ALISQDIDEESKSEMRKERINIIKNLSNDITQLKQLFIEKTELLDKSSSDLHNVVIEGTDKVLQ<br>AEQLRQKQLTEDIATKELERKEIEKKRDKIIEALDVIREHNLVDAFKDLIPTGENLSELDLAK<br>PEIELLKQSLEITKKLLGQFSEGLKYIDLTDARKKLDNQIDTASTRLTELNRQLEQSEKLIAG<br>VNAIIKIDQEKSAVVVEAEKLSRAWHIFIHEITALQGTSLNEVELSKPLIKQQIYLESLIKQLI*
YaxB$_{strepII\text{-}10aa}$

MSYYHHHHHHDYDIPTTENLYFQG**WSHPQFEK**GSSGSAGSAGAEISTFPHSGLSYPDIN<br>FKIFSQGVKNISHLAQFKTTGVEVLQEKALRVSLYSQRLDVIVRESLSSLQVKLENTLALTY<br>FTTLEEIDEALISQDIDEESKSEMRKERINIIKNLSNDITQLKQLFIEKTELLDKSSSDLHNVVII<br>EGTDKVLQAEQLRQKQLTEDIATKELERKEIEKKRDKIIEALDVIREHNLVDAFKDLIPTGEN<br>LSELDLAKPEIELLKQSLEITKKLLGQFSEGLKYIDLTDARKKLDNQIDTASTRLTELNRQLE<br>QSEKLIAGVNAIIKIDQEKSAVVVEAEKLSRAWHIFIHEITALQGTSLNEVELSKPLIKQQIYL<br>ESLIKQLI*
YaxB$_{strepII-30aa}$<br>MSYYHHHHHHDYDIPTTENLYFQG**WSHPQFEK**GSSGSTSNASAGTGSATGSNSTAGAS<br>SGNSAEISTFPHSGLSYPDINFKIFSQGVKNISHLAQFKTTGVEVLQEKALRVSLYSQRLDV<br>IVRESLSSLQVKLENTLALTYFTTLEEIDEALISQDIDEESKSEMRKERINIIKNLSNDITQLKQ<br>LFIEKTELLDKSSSDLHNVVIIEGTDKVLQAEQLRQKQLTEDIATKELERKEIEKKRDKIIEAL<br>DVIREHNLVDAFKDLIPTGENLSELDLAKPEIELLKQSLEITKKLLGQFSEGLKYIDLTDARK<br>KLDNQIDTASTRLTELNRQLEQSEKLIAGVNAIIKIDQEKSAVVVEAEKLSRAWHIFIHEITAL<br>QGTSLNEVELSKPLIKQQIYLESLIKQLI*
YaxB$_{strepII-32aa-AP}$<br>MSYYHHHHHHDYDIPTTENLYFQG**WSHPQFEK**APAPDDDAPAPAPAPAPAPAPAPAPAS<br>APGSSAEISTFPHSGLSYPDINFKIFSQGVKNISHLAQFKTTGVEVLQEKALRVSLYSQRLD<br>VIVRESLSSLQVKLENTLALTYFTTLEEIDEALISQDIDEESKSEMRKERINIIKNLSNDITQLK<br>QLFIEKTELLDKSSSDLHNVVIIEGTDKVLQAEQLRQKQLTEDIATKELERKEIEKKRDKIIEA<br>LDVIREHNLVDAFKDLIPTGENLSELDLAKPEIELLKQSLEITKKLLGQFSEGLKYIDLTDARK<br>KLDNQIDTASTRLTELNRQLEQSEKLIAGVNAIIKIDQEKSAVVVEAEKLSRAWHIFIHEITAL<br>QGTSLNEVELSKPLIKQQIYLESLIKQLI*
YaxB$_{strepII-31aa-EAAAK}$<br>MSYYHHHHHHDYDIPTTENLYFQG**WSHPQFEK**EAAAKDDDEAAAKEAAAKEAAAKEAAA<br>KGSSAEISTFPHSGLSYPDINFKIFSQGVKNISHLAQFKTTGVEVLQEKALRVSLYSQRLDV<br>IVRESLSSLQVKLENTLALTYFTTLEEIDEALISQDIDEESKSEMRKERINIIKNLSNDITQLKQ<br>LFIEKTELLDKSSSDLHNVVIIEGTDKVLQAEQLRQKQLTEDIATKELERKEIEKKRDKIIEAL<br>DVIREHNLVDAFKDLIPTGENLSELDLAKPEIELLKQSLEITKKLLGQFSEGLKYIDLTDARK<br>KLDNQIDTASTRLTELNRQLEQSEKLIAGVNAIIKIDQEKSAVVVEAEKLSRAWHIFIHEITAL<br>QGTSLNEVELSKPLIKQQIYLESLIKQLI*
YaxB$_{strepII-50aa}$<br>MSYYHHHHHHDYDIPTTENLYFQG**WSHPQFEK**GSSGSTSNASAGTGSATGSNSTAGAS<br>SGNSAGTGTSSGSTGSANSSAGTGAEISTFPHSGLSYPDINFKIFSQGVKNISHLAQFKTT<br>GVEVLQEKALRVSLYSQRLDVIVRESLSSLQVKLENTLALTYFTTLEEIDEALISQDIDEESK<br>SEMRKERINIIKNLSNDITQLKQLFIEKTELLDKSSSDLHNVVIIEGTDKVLQAEQLRQKQLT<br>EDIATKELERKEIEKKRDKIIEALDVIREHNLVDAFKDLIPTGENLSELDLAKPEIELLKQSLEI<br>TKKLLGQFSEGLKYIDLTDARKKLDNQIDTASTRLTELNRQLEQSEKLIAGVNAIIKIDQEKS<br>AVVVEAEKLSRAWHIFIHEITALQGTSLNEVELSKPLIKQQIYLESLIKQLI*
YaxB$_{weak-50aa}$<br>MSYYHHHHHHDYDIPTTENLYFQG**HDHPQNL**GSSGSTSNASAGTGSATGSNSTAGASS<br>GNSAGTGTSSGSTGSANSSAGTGAEISTFPHSGLSYPDINFKIFSQGVKNISHLAQFKTTG<br>VEVLQEKALRVSLYSQRLDVIVRESLSSLQVKLENTLALTYFTTLEEIDEALISQDIDEESKS<br>EMRKERINIIKNLSNDITQLKQLFIEKTELLDKSSSDLHNVVIIEGTDKVLQAEQLRQKQLTE<br>DIATKELERKEIEKKRDKIIEALDVIREHNLVDAFKDLIPTGENLSELDLAKPEIELLKQSLEIT<br>KKLLGQFSEGLKYIDLTDARKKLDNQIDTASTRLTELNRQLEQSEKLIAGVNAIIKIDQEKSA<br>VVVEAEKLSRAWHIFIHEITALQGTSLNEVELSKPLIKQQIYLESLIKQLI*
YaxB$_{-58aa}$<br>MSYYHHHHHHDYDIPTTENLYFQGGTSGTSGTGSSGSTSNASAGTGSATGSNSTAGAS<br>SGNSAGTGTSSGSTGSANSSAGTGAEISTFPHSGLSYPDINFKIFSQGVKNISHLAQFKTT<br>GVEVLQEKALRVSLYSQRLDVIVRESLSSLQVKLENTLALTYFTTLEEIDEALISQDIDEESK<br>SEMRKERINIIKNLSNDITQLKQLFIEKTELLDKSSSDLHNVVIIEGTDKVLQAEQLRQKQLT<br>EDIATKELERKEIEKKRDKIIEALDVIREHNLVDAFKDLIPTGENLSELDLAKPEIELLKQSLEI<br>TKKLLGQFSEGLKYIDLTDARKKLDNQIDTASTRLTELNRQLEQSEKLIAGVNAIIKIDQEKS<br>AVVVEAEKLSRAWHIFIHEITALQGTSLNEVELSKPLIKQQIYLESLIKQLI*

$\text{YaxB}_{\text{strepII-70aa}}$<br>MSYYHHHHHHDYDIPTTENLYFQG**WSHPQFEK**GSSGSTSNASAGTGSATGSNSTAGAS<br>SGNSAGTGTSSGSTGSANSSAGTGSGSATASAGNSGTSTAGNSTAEISTFPHSGLSYPD<br>INFKIFSQGVKNISHLAQFKTTGVEVLQEKALRVSLYSQRLDVIVRESLSSLQVKLENTLALT<br>YFTTLEEIDEALISQDIDEESKSEMRKERINIIKNLSNDITQLKQLFIEKTELLDKSSSDLHNV<br>VIIEGTDKVLQAEQLRQKQLTEDIATKELERKEIEKKRDKIIEALDVIREHNLVDAFKDLIPTG<br>ENLSELDLAKPEIELLKQSLEITKKLLGQFSEGLKYIDLTDARKKLDNQIDTASTRLTELNRQ<br>LEQSEKLIAGVNAIIKIDQEKSAVVVEAEKLSRAWHIFIHEITALQGTSLNEVELSKPLIKQQI<br>YLESLIKQLI*
$\text{YaxB}_{\text{IS20-70aa}}$<br>MSYYHHHHHHDYDIPTTENLYFQG**ISKQGLGGDFEEIPSDEIIE**GSSGSTSNASAGTGSAT<br>GSNSTAGASSGNSAGTGTSSGSTGSANSSAGTGSGSATASAGNSGTSTAGNSTAEISTF<br>PHSGLSYPDINFKIFSQGVKNISHLAQFKTTGVEVLQEKALRVSLYSQRLDVIVRESLSSLQ<br>VKLENTLALTYFTTLEEIDEALISQDIDEESKSEMRKERINIIKNLSNDITQLKQLFIEKTELLD<br>KSSSDLHNVVIIEGTDKVLQAEQLRQKQLTEDIATKELERKEIEKKRDKIIEALDVIREHNLV<br>DAFKDLIPTGENLSELDLAKPEIELLKQSLEITKKLLGQFSEGLKYIDLTDARKKLDNQIDTA<br>STRLTELNRQLEQSEKLIAGVNAIIKIDQEKSAVVVEAEKLSRAWHIFIHEITALQGTSLNEV<br>ELSKPLIKQQIYLESLIKQLI*G
$\text{YaxB}_{\text{strepII-100aa}}$<br>MSYYHHHHHHDYDIPTTENLYFQG**WSHPQFEK**GSSGSTSNASAGTGSATGSNSTAGAS<br>SGNLGSSGSTSNASAGTGSATGSNSTAGASSGNSAGTGTSSGSTGSANSSAGTGSGSA<br>TASAGNSGTSTAGNSTAEISTFPHSGLSYPDINFKIFSQGVKNISHLAQFKTTGVEVLQEK<br>ALRVSLYSQRLDVIVRESLSSLQVKLENTLALTYFTTLEEIDEALISQDIDEESKSEMRKERI<br>NIIKNLSNDITQLKQLFIEKTELLDKSSSDLHNVVIIEGTDKVLQAEQLRQKQLTEDIATKELE<br>RKEIEKKRDKIIEALDVIREHNLVDAFKDLIPTGENLSELDLAKPEIELLKQSLEITKKLLGQF<br>SEGLKYIDLTDARKKLDNQIDTASTRLTELNRQLEQSEKLIAGVNAIIKIDQEKSAVVVEAEK<br>LSRAWHIFIHEITALQGTSLNEVELSKPLIKQQIYLESLIKQLI*

Supporting Table 3: Synthetic gene sequence of flexible linker in this work. DNA sequences of $\text{YaxA}_{\Delta 40}$ and YaxB_{WT} can be found in ref.[1]

gBlock for flexible linker (5' > 3')<br>GCACTACTCATATGCATCACCATCAAGGGTCTAGTGGAAGTACCTCAAATGCGAGTG<br>CCGGTACAGGAAGCGCAACAGGAAGTAACTCTACGGCAGGAGCGAGTTCTGGAAAT<br>AGCGCGGGAACAGGGACGAGTAGCGGGTCCACAGGAAGTGCCAATAGCAGCGCTG<br>GTACAGGGAGCGGATCCGCAACTGCCTCGGCTGGTAATTCAGGAACAAGCACGGCT<br>GGAAACTCGACGAGCGGCTAGAAGCTTCATGAC

Supporting Table 4: Primers used in this work. Primer numbers indicated are used for obtaining PCR-fragments of each construct in this work, see also Supporting Table 5. Uracil (U) is highlighted in **red**.

Primer #	Sequence (5' > 3')
**43**	ACATATGTA**U**ATCTCCTTCTTAAAGTTAAACAAAATTAT
**67**	AGACCCGT**U**TAGAGGC
**68**	AACGGGTC**U**TGAGGG
**76**	ATACATATG**U**CGTACTACCATCACCATC
**77**	AACCCGC**U**GATCCAGAACTTCCTTTCTCAAACTGAGGGTGACTC
**78**	AGCGGGT**U**CGGCTGGGGCCGAAATAAGCACATTTC
**89**	ACTAGACCC**U**TTCTCAAACTGAGG
**90**	AGGGTCTAG**U**GGAAGTACCTC
**91**	ATGTGCTTA**U**TTCGGCGCTATTTCCAGAACTCGCTCC

**93**	ATGTGCTTAUTTCGGCCCCTGTACCAGCGCT
**94**	ATGTGCTTAUTTCGGCCGTCGAGTTTCCAGCCG
**95**	ATAAGCACAUTTCCACACAG
**105**	ACTAGACCCUGTTCCTGACGTACCTGAGGTCCCTCCCTGAAAATACAGGTTTTCGG
**106**	ACTAGACCCUAAATTCTGTGGGTGATCGTGTCCCTGAAAATACAGGTTTTCGG
**107**	AGGCAGCUGCGAAAGAAGCGGCAGCAAAGGAGGCAGCGGCAAAGGGATCAAGCGCCGAAATAAG
**108**	AGCTGCCUCTTTTGCCGCGGCCTCATCGTCATCTTTAGCCGCAGCCTCTTTCTCAAACTGAGGGTGACTC
**109**	ACCGGCUCCCGCTCCGGCCCCAGCTCCCGCTCCTGCCCCCGCAAGTGCTCCGGGATCAAGCGCCGAAATAAG
**110**	AGCCGGUGCTGGTGCAGGCGCATCGTCATCTGGGGCTGGTGCTTTCTCAAACTGAGGGTGACTC
**149**	ACCCAAGAUTTCCAGAACTCGCTCCTG
**150**	ATCTTGGGUCTAGTGGAAGTACC
**157**	AAAATCACCUCCTAAGCCTTGCTTGCTAATTCCCTGAAAATACAGGTTTTCGG
**158**	AGGTGATTTUGAAGAGATTCCCTCCGACGAAATTATCGAAGGGTCTAGTGGAAGTACCTC

Supporting Table 5: Overview of primers used for obtaining PCR-fragment of each construct. Primers are indicated by a number corresponding sequence can be found in Supporting Table 4. For primer combinations indicated with (*), the synthetic gene in Supporting Table 3 was used as template. For primer combinations indicated with (#), the pRSET-A-YaxB$_{strepII-50aa}$ plasmid was used as template. For primer combinations indicated with (+), the pRSET-A-YaxB$_{strepII-70aa}$ plasmid was used as template. All other PCR-fragments were obtained with pRSET-A-YaxB$_{WT}$ plasmid (from ref.[1]) as template.

**Name**	**Tag**		**Linker**		**YaxB**		**Vector (pRSET-A)**	
	fw	*rv*	*fw*	*rv*	*fw*	*rv*	*fw*	*rv*
**10aa**	76	77	78	-	-	67	68	43
**30aa**	76	89	90*	91*	95	67	68	43
**50aa**	76	89	90*	93*	95	67	68	43
**70aa**	76	89	90*	94*	95	67	68	43
**100aa**	76+	149+	150+	-	-	67+	68	43
**50aa no tag**	76	105	90#	-	-	67#	68	43
**50aa weak tag**	76	106	90#	-	-	67#	68	43
**30aa EAAAK**	76	108	107	-	-	67	68	43
**30aa AP**	76	110	109	-	-	67	68	43
**70aa IS20 tag**	76+	157+	158+	-	-	67+	68	43

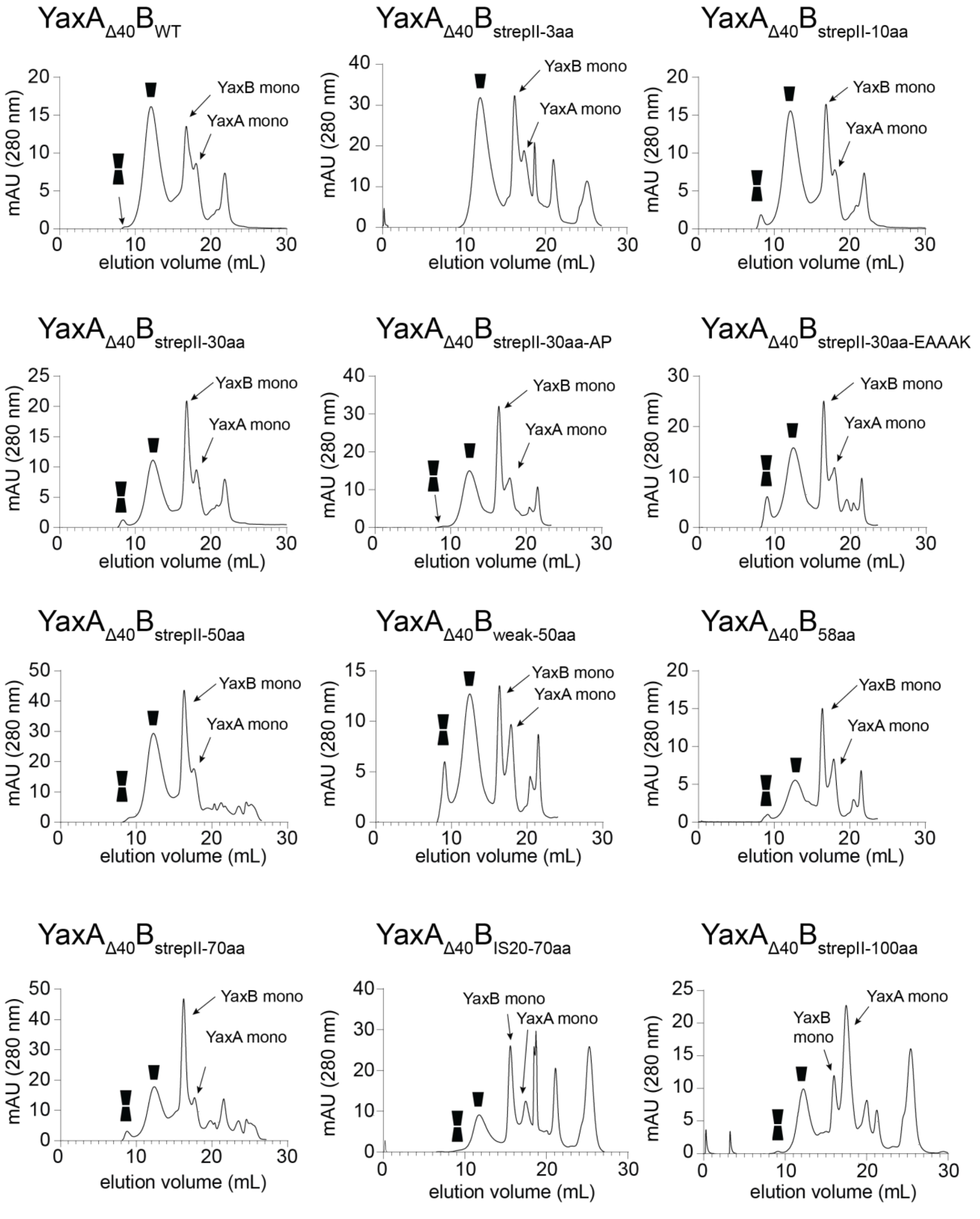

Supporting Figure 1: YaxAB oligomer variant purifications on Superose 6-FPLC used in this work. YaxAB fast protein liquid chromatography (FPLC) elution profiles on size exclusion chromatography (SEC) Superose-6 column. Per YaxAB-variant the peaks are indicated: the hourglass-shaped nanopore dimers, the individual oligomers, monomeric YaxA and YaxB components. SEC buffer: 150 mM NaCl, 25 mM HEPES pH 7.0, 0.05% cymal-6, Superose-6 column, 1 mL/min flow rate.

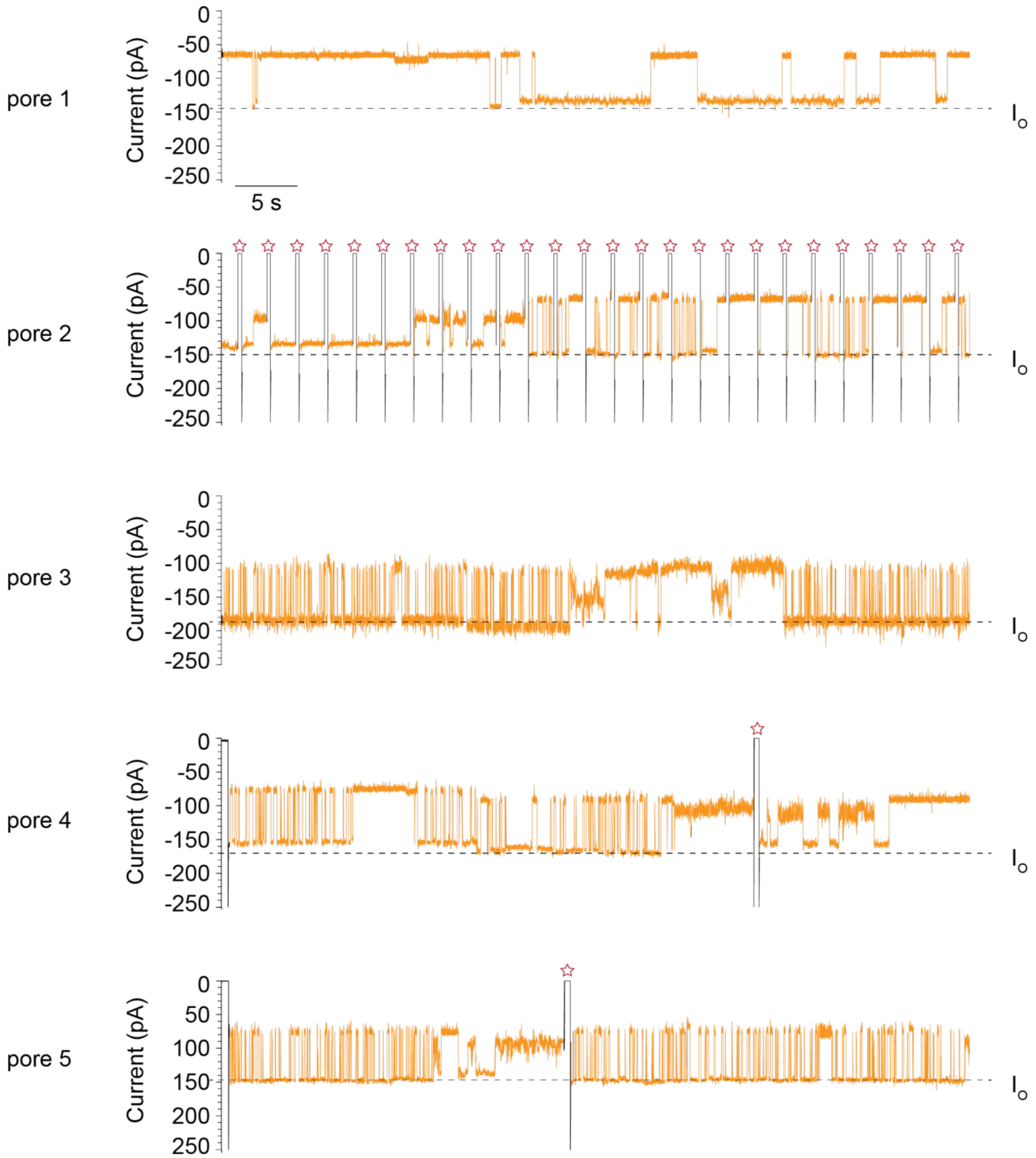

Supporting Figure 2: YaxA$_{\Delta 40}$B$_{strepII\text{-}3aa}$$^{2.3*}$ (3 aa) capturing SA. SA proteins (orange) were captured and released by YaxA$_{\Delta 40}$B$_{strepII\text{-}3aa}$$^{2.3*}$. Open pore current ($I_O$) is indicated by black dotted line. Flipping of potential (+/– 75 mV) is indicated by red star. 20 nM SA was added to *cis*. Measurements were conducted at –75 mV, at 150 mM NaCl, 15 mM TrisHCl pH 7.5, with DPhPC lipids composing the bilayer. Data were recorded at 50 kHz sampling rate, and 10 kHz Bessel filter. Traces were additionally filtered with 500 Hz low-pass Gaussian filter for visualization.

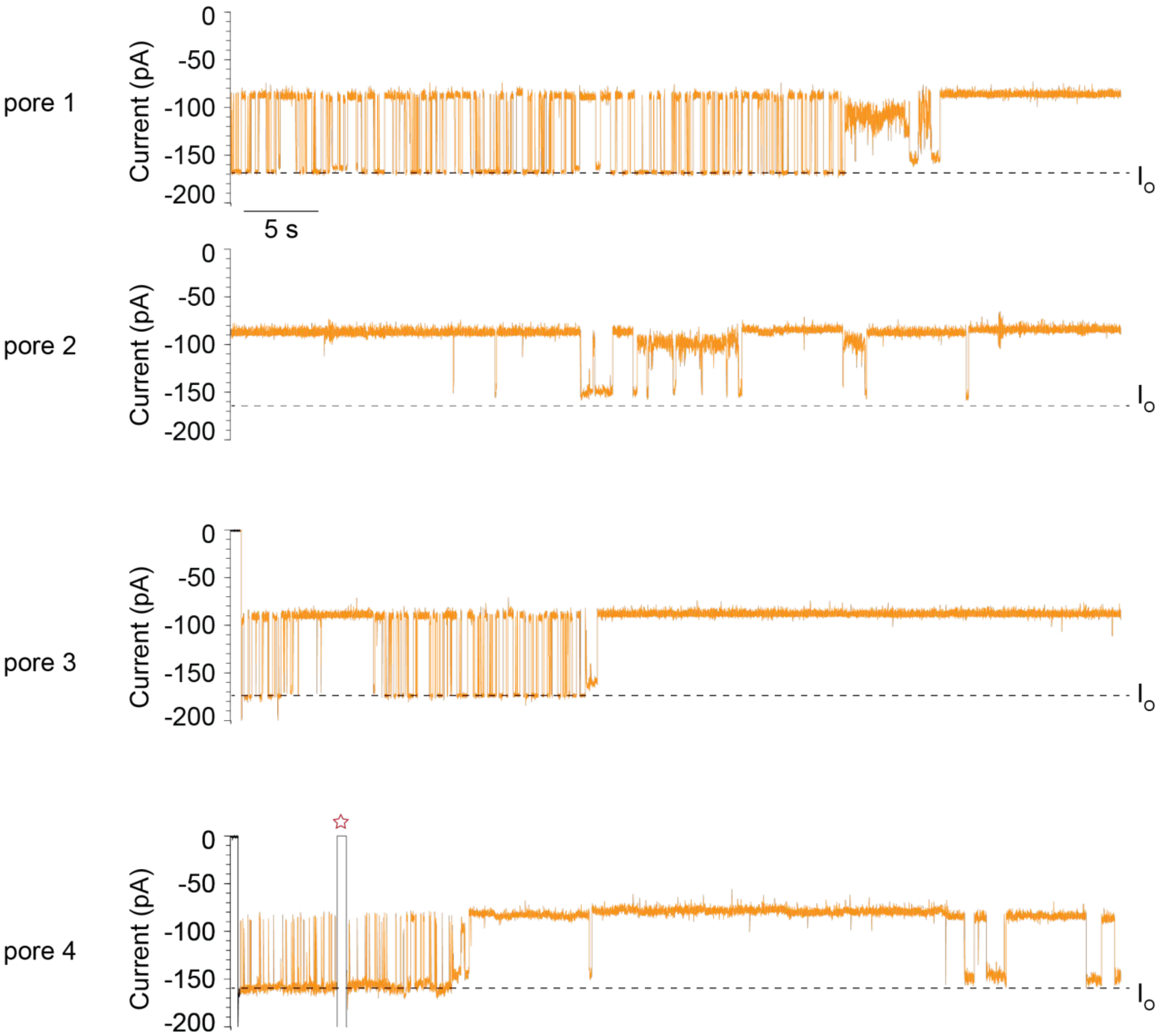

Supporting Figure 3: YaxA$_{\Delta 40}$B$_{strepII\text{-}10aa}$$^{2.3*}$ (10 aa) capturing SA. SA proteins (orange) were captured and released by YaxA$_{\Delta 40}$B$_{strepII\text{-}10aa}$$^{2.3*}$. I_O indicated by black dotted line. Flipping of potential (+/– 75 mV) indicated by red star. 20 nM SA was added to *cis*. Measurements were conducted at –75 mV, at 150 mM NaCl, 15 mM TrisHCl pH 7.5, with DPhPC lipids composing the bilayer. Data were recorded at 50 kHz sampling rate, and 10 kHz Bessel filter. Traces were additionally filtered with 500 Hz low-pass Gaussian filter for visualization.

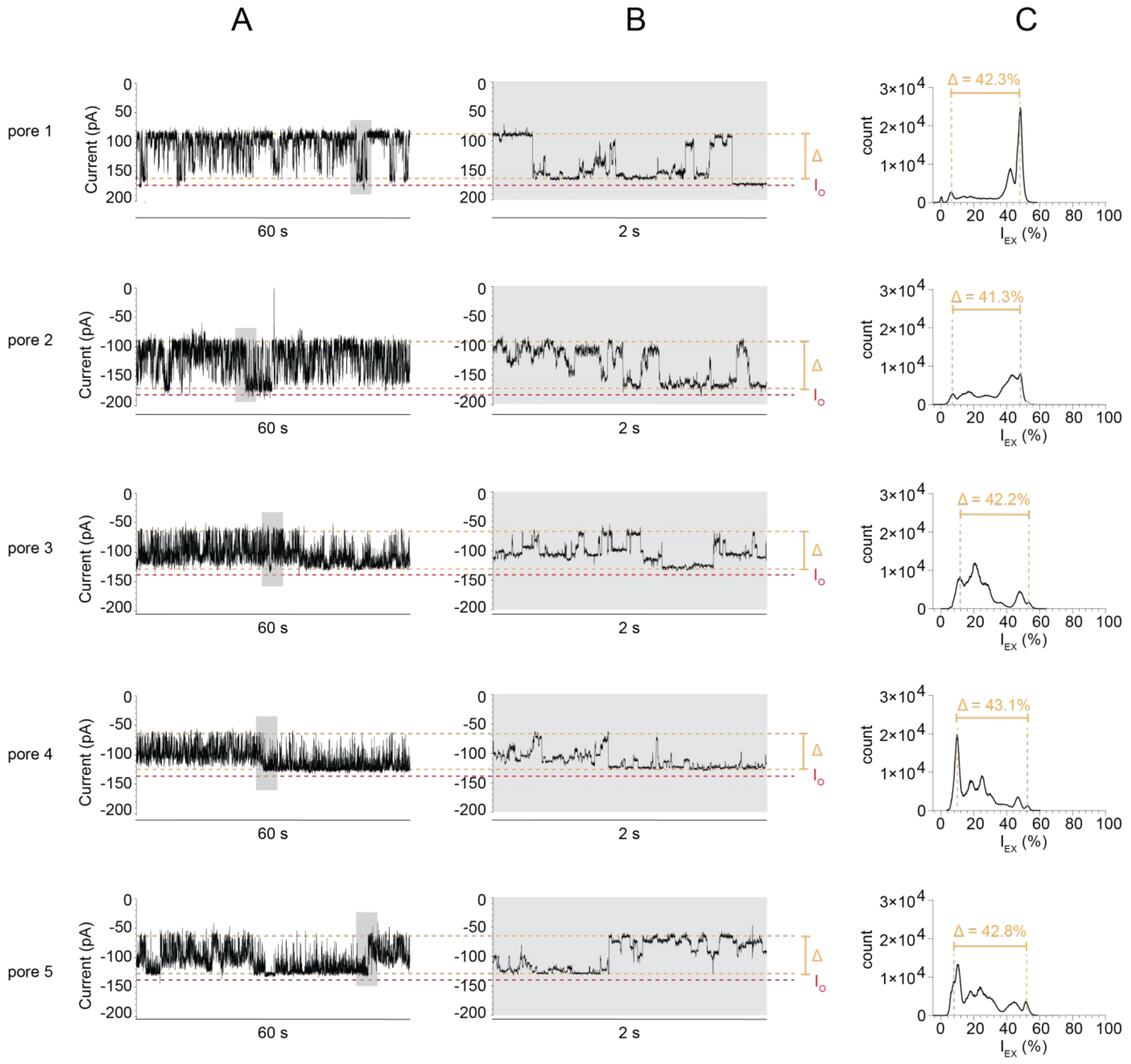

Supporting Figure 4: YaxA$_{\Delta 40}$B$_{strepII\text{-}30aa}$$^{1.9*/2.3*}$ (30 aa) caging SA. A) Five representative electrophysiology traces (60 s) showing the binding of SA to YaxA$_{\Delta 40}$B$_{strepII\text{-}30aa}$. I_O is indicated by red dotted line. Outer blockade-boundaries are indicated by orange dotted line, spanning a bandwidth indicated by Δ. **B)** Zoom-in trace (2 s) of the corresponding section in panel A. **C)** All-point histograms (60 s) of the trace in showed in panel A, showing the delta between outer I_{EX}-boundaries (ΔI_{EX}, orange). $\Delta I_{EX,30aa}$ = 42.34 ± 0.60 Δ% (N=5, error bars represent standard deviation). 20 nM SA was added to *cis*. Measurements were conducted at –75 mV, at 150 mM NaCl, 15 mM TrisHCl pH 7.5, with DPhPC lipids composing the bilayer. 1.9* and 2.3* nS pores were used. Data were recorded at 50 kHz sampling rate, and 10 kHz Bessel filter. Traces were additionally filtered with 500 Hz low-pass Gaussian filter for all-point histogram (0.1 pA bin size) and for visualization. Inhouse MATLAB script was used to calculate I_{EX} of outer peaks and the ΔI_{EX}

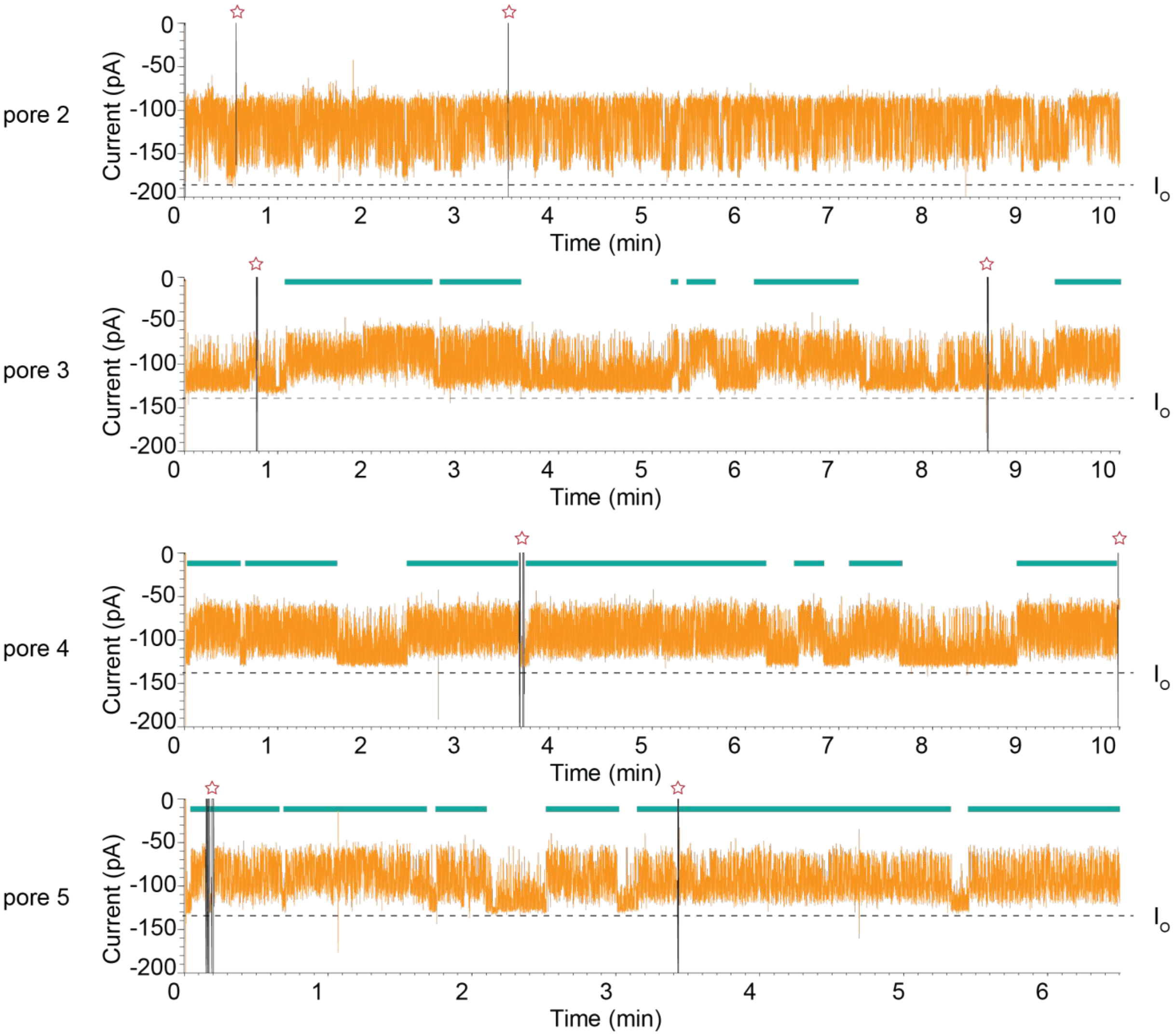

Supporting Figure 5: Macroscale SA caging by $YaxA_{\Delta40}B_{strepII\text{-}30aa}{}^{1.9^*/2.3^*}$ (30aa). Representative electrophysiology traces of at least 6 min showing the binding of SA (orange) to $YaxA_{\Delta40}B_{strepII\text{-}30aa}$. I_O indicated by black dotted line. Flipping of potential (+/– 75 mV) indicated by red star. Teal squares indicate macrostates in the multilevel. 20 nM SA was added to *cis*. 1.9* and 2.3* nS pores were used. Measurements were conducted at –75 mV, at 150 mM NaCl, 15 mM TrisHCl pH 7.5, with DPhPC lipids composing the bilayer. Data were recorded at 50 kHz sampling rate, and 10 kHz Bessel filter. Traces were additionally filtered with 500 Hz low-pass Gaussian filter for visualization

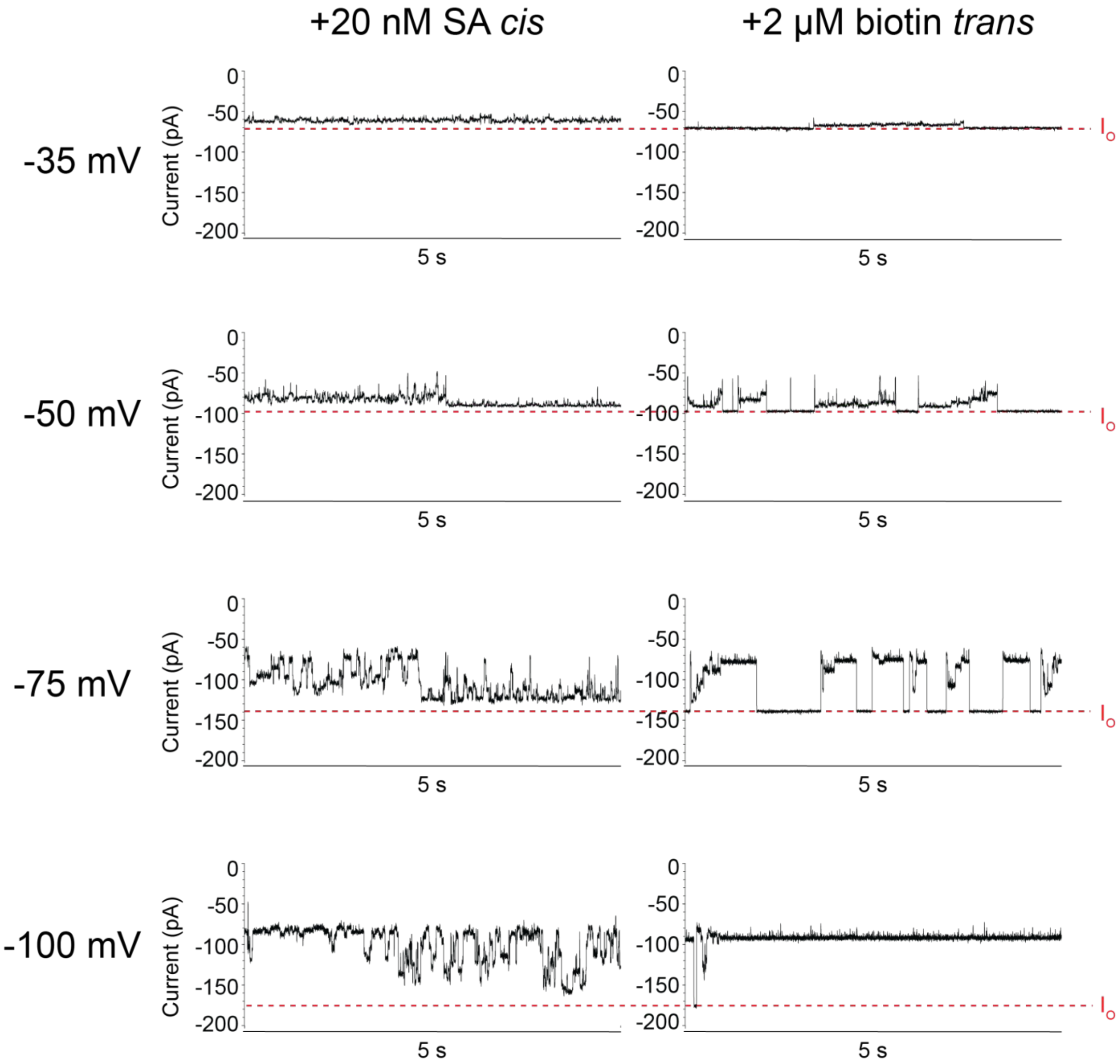

Supporting Figure 6: Voltage dependence of SA-blockades in $YaxA_{\Delta40}B_{strepII\text{-}30aa}^{1.9*}$ (30aa) and biotin release. SA-blockades (left) and SA+biotin blockades (right) detected by $YaxA_{\Delta40}B_{strepII\text{-}30aa}^{1.9*}$ at different voltages. I_O is indicated by red dotted line. SA was added to *cis* at 20 nM. Biotin was added to *trans* at 2 µM. Measurements were conducted at –75 mV, in 150 mM NaCl, 15 mM TrisHCl pH 7.5, with DPhPC lipids composing the bilayer. Data were recorded at 50 kHz sampling rate, and 10 kHz Bessel filter. Traces were additionally filtered with 500 Hz low-pass Gaussian filter for visualization.

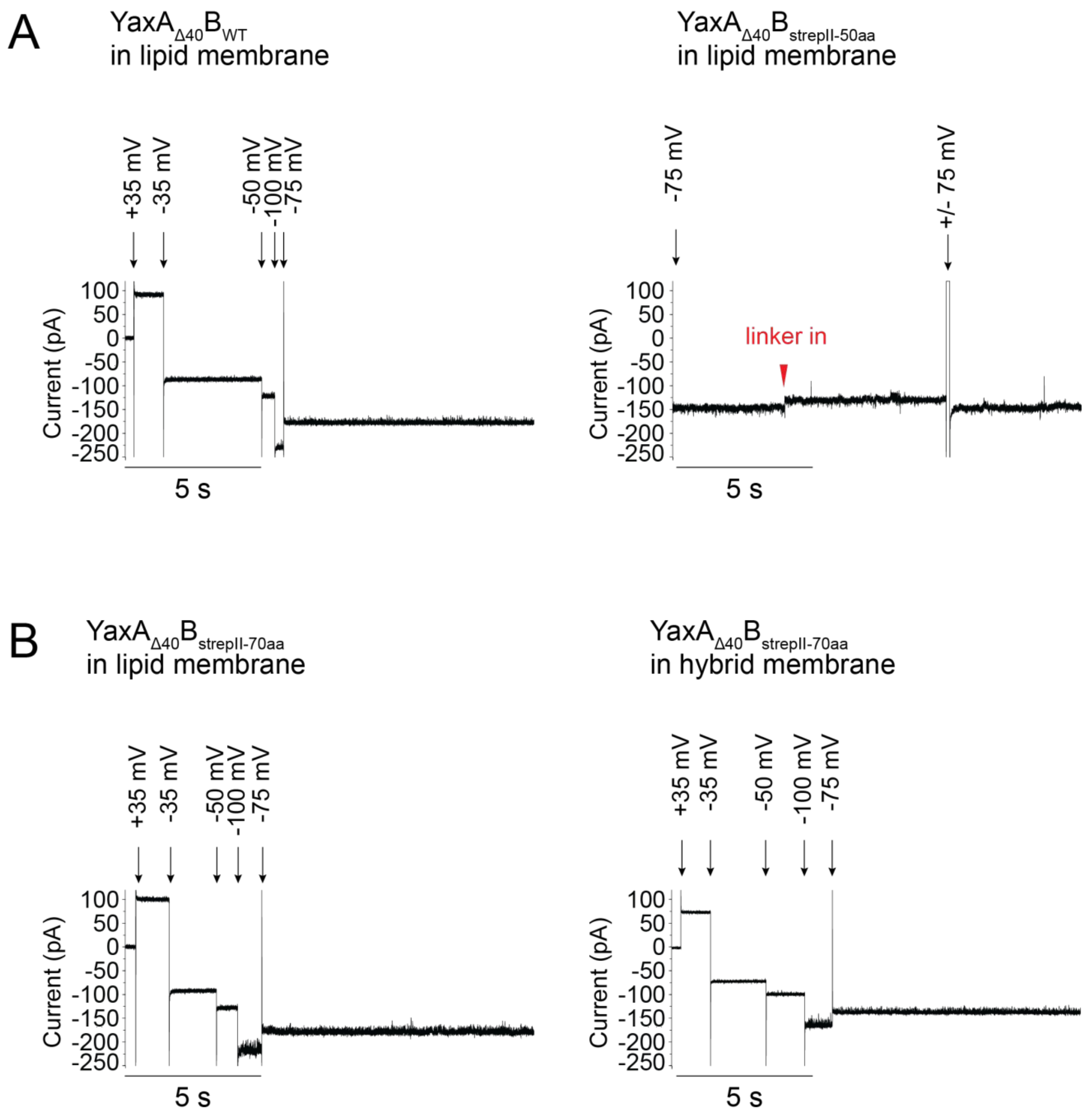

Supporting Figure 7: Linker capture and effect of the amphipathic membrane on YaxA$_{\Delta40}$B recordings. A) (left) Typical I_O of YaxA$_{\Delta40}$B$_{WT}$ in DPhPC lipid membrane; **(right)** Rare case of linker entering (red arrow) the nanopore, in this case YaxA$_{\Delta40}$B$_{strepII\text{-}50aa}^{2.3*}$. Flipping the potential (+75 mV to –75 mV) is indicated. **B) (left)** Typical I_O of YaxA$_{\Delta40}$B$_{strepII\text{-}70aa}^{2.3*}$ in DPhPC lipid membrane; **(right)** Typical I_O of YaxA$_{\Delta40}$B$_{strepII\text{-}70aa}^{1.9*}$ in PDB$_{11}$PEO$_8$:DPhPC (1:1)-hybrid membrane. Measurements were conducted in 150 mM NaCl, 15 mM TrisHCl pH 7.5. Data were recorded at 50 kHz sampling rate, and 10 kHz Bessel filter. Traces were additionally filtered with 2 kHz low-pass Gaussian filter for visualization.

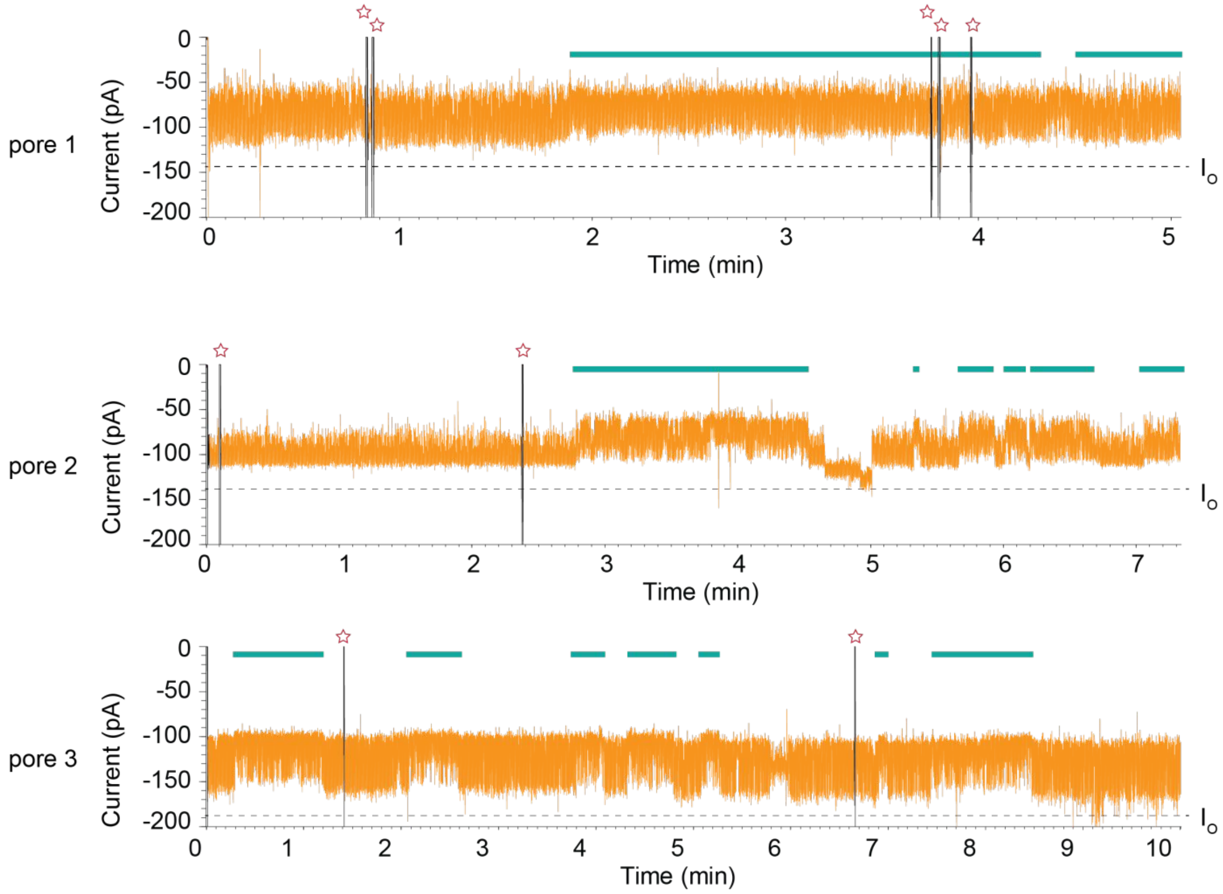

Supporting Figure 8: Macroscale SA caging by $YaxA_{\Delta40}B_{strepII\text{-}50aa}{}^{1.9*/2.3*/2.6*}$ (50aa). Three representative electrophysiology traces of at least 5 min showing the binding of SA (orange) to $YaxA_{\Delta40}B_{strepII\text{-}50aa}{}^{1.9*/2.3*/2.6*}$. I_O indicated by black dotted line. Flipping of potential (+/– 75 mV) indicated by red star. Teal squares indicate macrostates in the multilevel. 20 nM SA was added to *cis*. 1.9*, 2.3*, and 2.6* nS pores were used. Measurements were conducted at –75 mV, at 150 mM NaCl, 15 mM TrisHCl pH 7.5, with DPhPC lipids composing the bilayer. Data were recorded at 50 kHz sampling rate, and 10 kHz Bessel filter. Traces were additionally filtered with 500 Hz low-pass Gaussian filter for visualization.

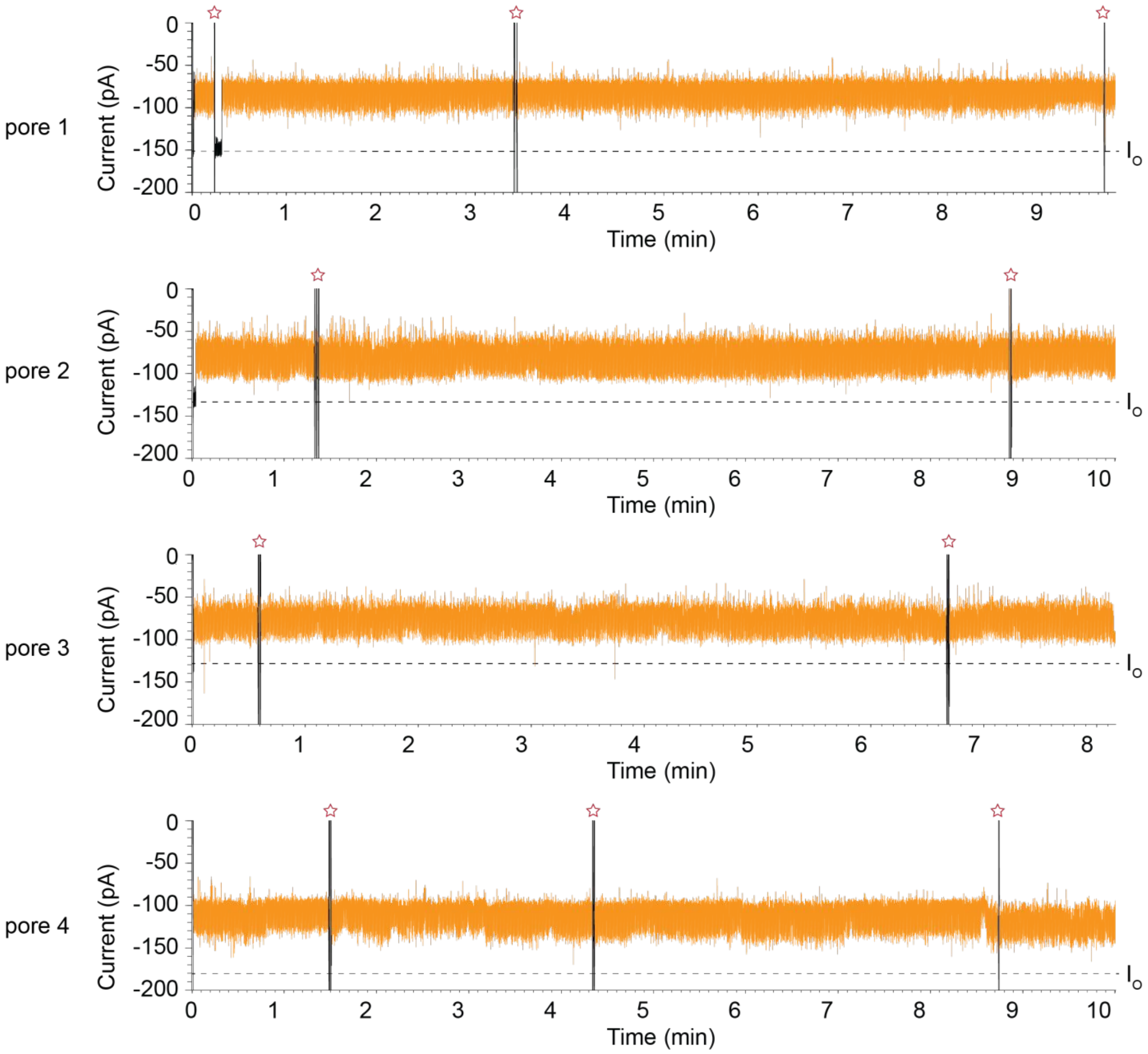

Supporting Figure 9: Macroscale SA caging by YaxA$_{\Delta40}$B$_{strepII\text{-}70aa}$$^{1.9*/2.3*/2.6*}$ (70 aa). Four representative electrophysiology traces of at least 8 min showing the binding of SA (orange) to a YaxA$_{\Delta40}$B$_{strepII\text{-}70aa}$$^{1.9*/2.3*/2.6*}$ pore. I_O indicated by black dotted line. Flipping of potential (+/– 75 mV) indicated by red star. 20 nM SA was added to *cis*. 1.9*, 2.3*, 2.6* nS pores were used. Measurements were conducted at –75 mV, at 150 mM NaCl, 15 mM TrisHCl pH 7.5, with DPhPC lipids composing the bilayer. Data were recorded at 50 kHz sampling rate, and 10 kHz Bessel filter. Traces were additionally filtered with 500 Hz low-pass Gaussian filter and for visualization.

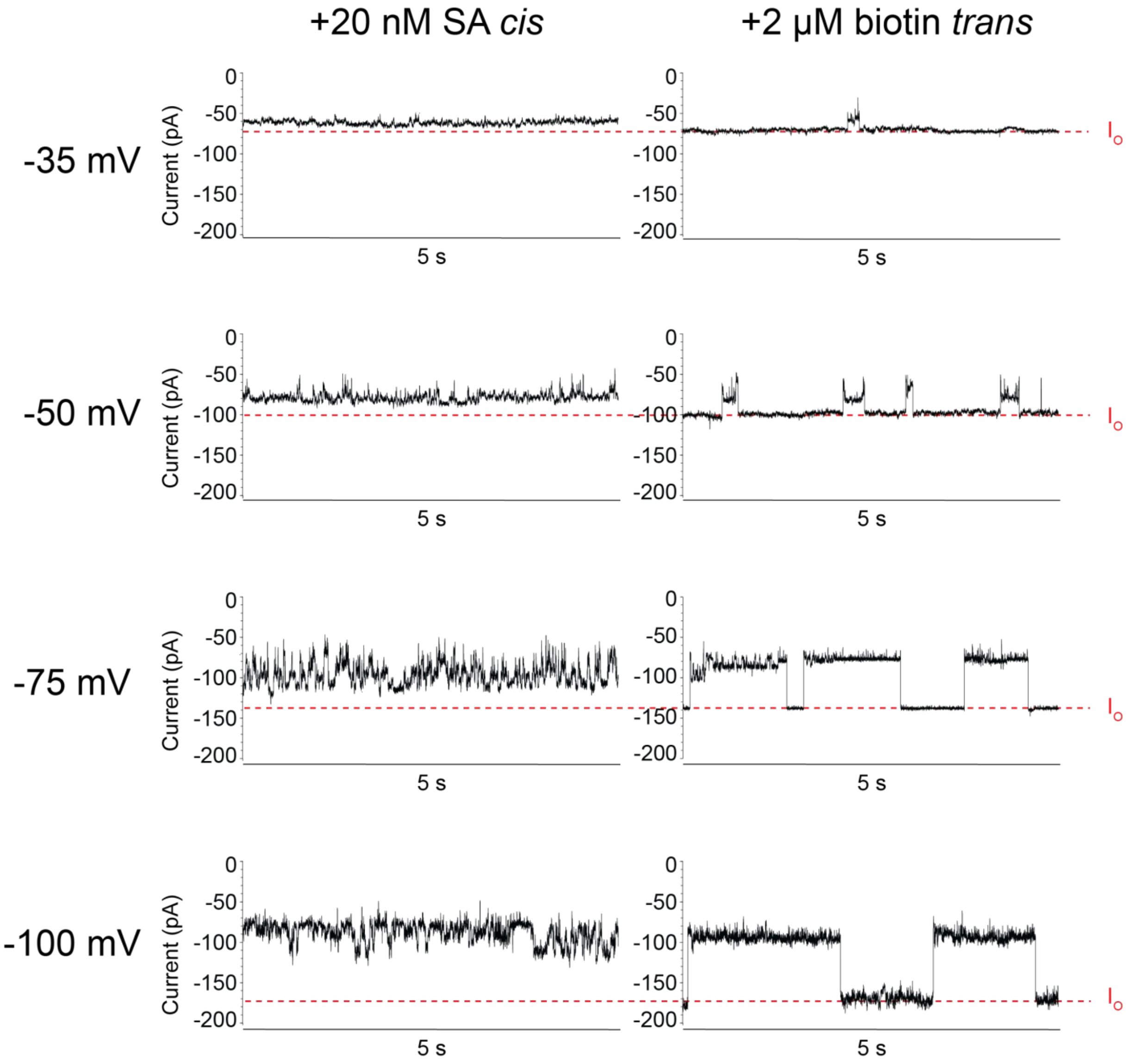

Supporting Figure 10: Voltage dependence of SA-blockades in YaxA$_{\Delta 40}$B$_{strepII\text{-}50aa}$$^{1.9*}$ (50aa) and biotin release. SA-blockades (left) and SA+biotin blockades (right) detected by YaxA$_{\Delta 40}$B$_{strepII\text{-}50aa}$$^{1.9*}$ at different voltages. I_O is indicated by red dotted line. SA was added to *cis* at 20 nM. Biotin was added to *trans* at 2 µM. Measurements were conducted at –75 mV, in 150 mM NaCl, 15 mM TrisHCl pH 7.5, with DPhPC lipids composing the bilayer. Data were recorded at 50 kHz sampling rate, and 10 kHz Bessel filter. Traces were additionally filtered with 500 Hz low-pass Gaussian filter for visualization.

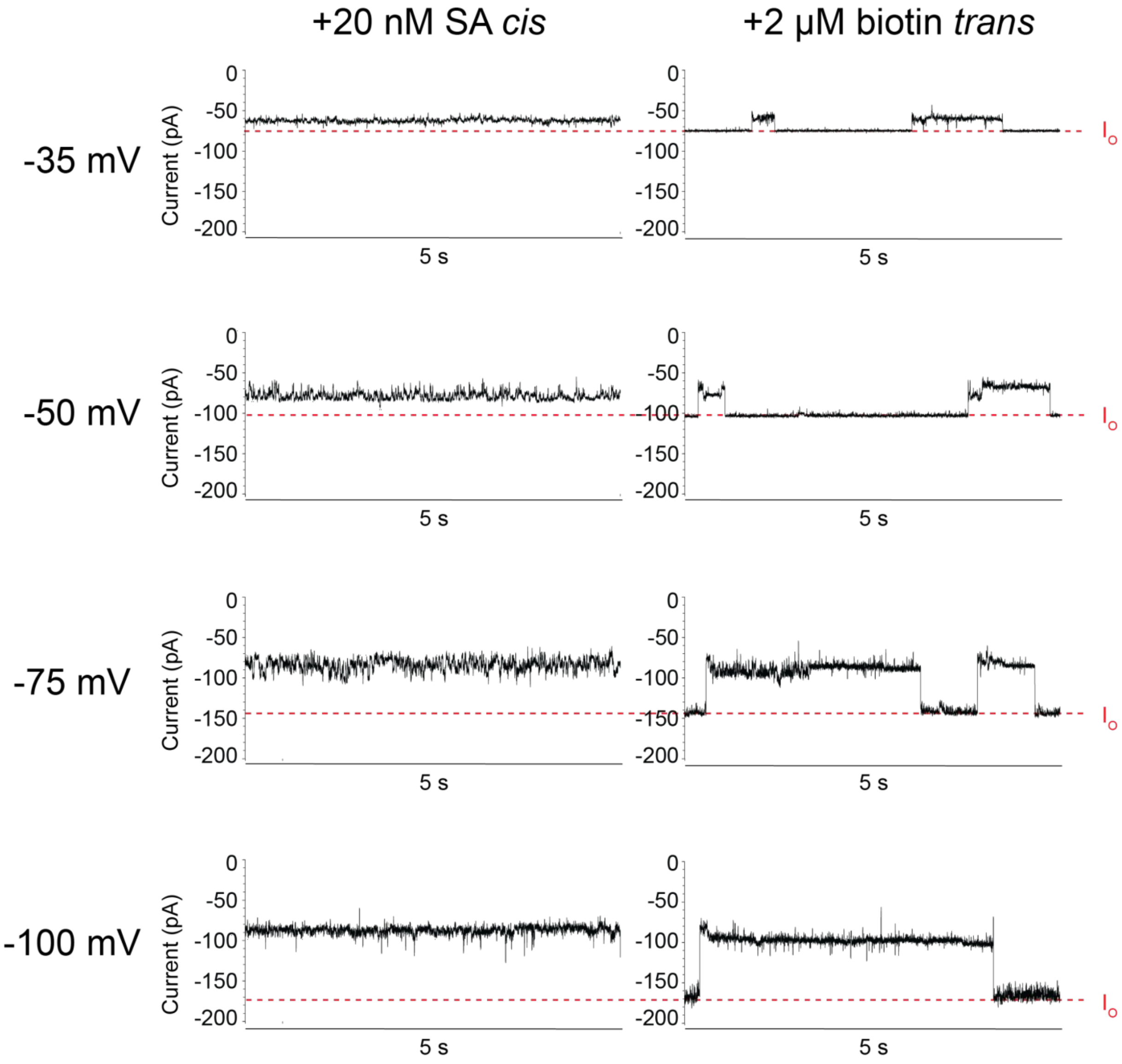

Supporting Figure 11: Voltage dependence of SA-blockades in $YaxA_{\Delta40}B_{strepII\text{-}70aa}^{2.3*}$ (70 aa) and biotin release. SA-blockades (left) and SA+biotin blockades (right) detected by $YaxA_{\Delta40}B_{strepII\text{-}70aa}^{2.3*}$ at different voltages. I_O is indicated by red dotted line. SA was added to *cis* at 20 nM. Biotin was added to *trans* at 2 μM. Measurements were conducted at –75 mV, in 150 mM NaCl, 15 mM TrisHCl pH 7.5, with DPhPC lipids composing the bilayer. Data were recorded at 50 kHz sampling rate, and 10 kHz Bessel filter. Traces were additionally filtered with 500 Hz low-pass Gaussian filter for visualization.

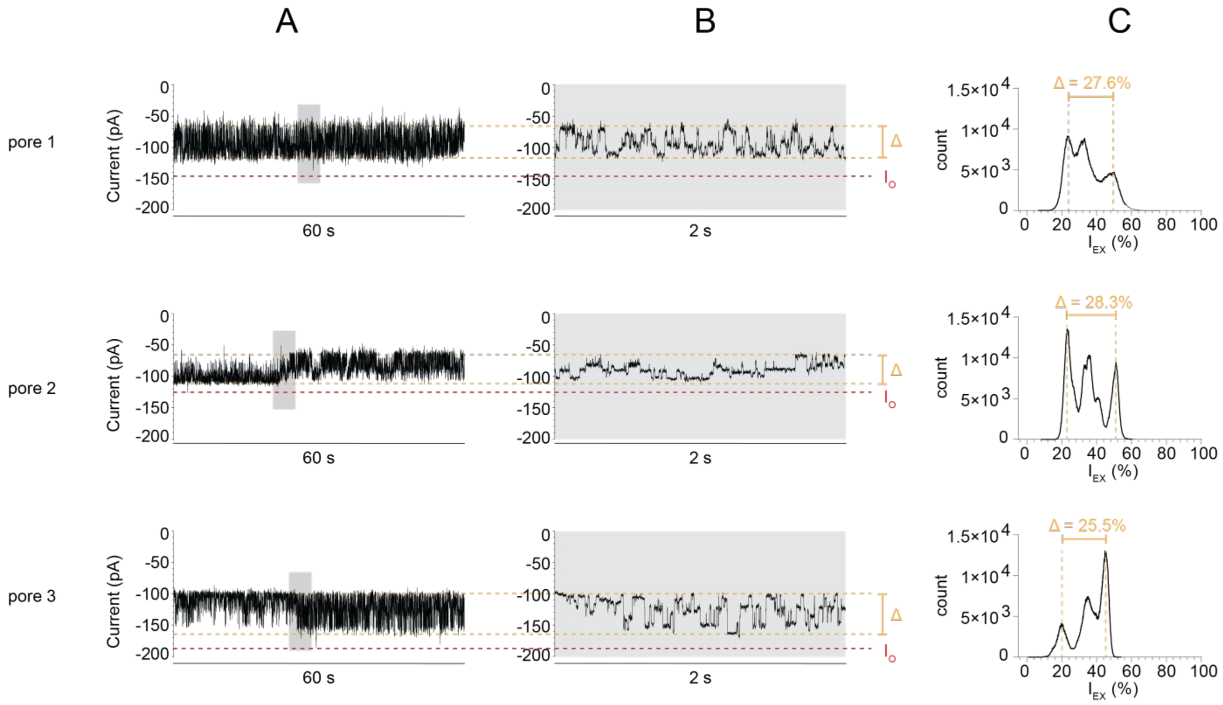

Supporting Figure 12: YaxA$_{\Delta 40}$B$_{strepII\text{-}50aa}$$^{1.9*/2.3*/2.6*}$ (50aa) caging of SA. A) Three representative electrophysiology traces (60 s) of SA bound to a YaxA$_{\Delta 40}$B$_{strepII\text{-}50aa}$$^{1.9*/2.3*/2.6*}$ pore. I_O is indicated by red dotted line. Outer blockade-boundaries are indicated by orange dotted line, spanning a bandwidth indicated by Δ. **B)** Zoom-in traces (2 s) of the corresponding section in panel A. **C)** All-point histograms (60 s) of the trace in showed in panel A, showing the delta between outer I_{EX}-boundaries (ΔI_{EX}, orange). $\Delta I_{EX,50aa}$ = 27.14 ± 1.16 Δ% (N=3, error bars represent standard deviation). 20 nM SA was added to *cis*. 1.9*, 2.3*, 2.6* nS pores were used. Measurements were conducted at –75 mV, at 150 mM NaCl, 15 mM TrisHCl pH 7.5, with DPhPC lipids composing the bilayer. Data were recorded at 50 kHz sampling rate, and 10 kHz Bessel filter. Traces were additionally filtered with 500 Hz low-pass Gaussian filter for all-point histogram (0.1 pA bin size) and for visualization. Inhouse MATLAB script was used to calculate I_{EX} of outer peaks and the ΔI_{EX}.

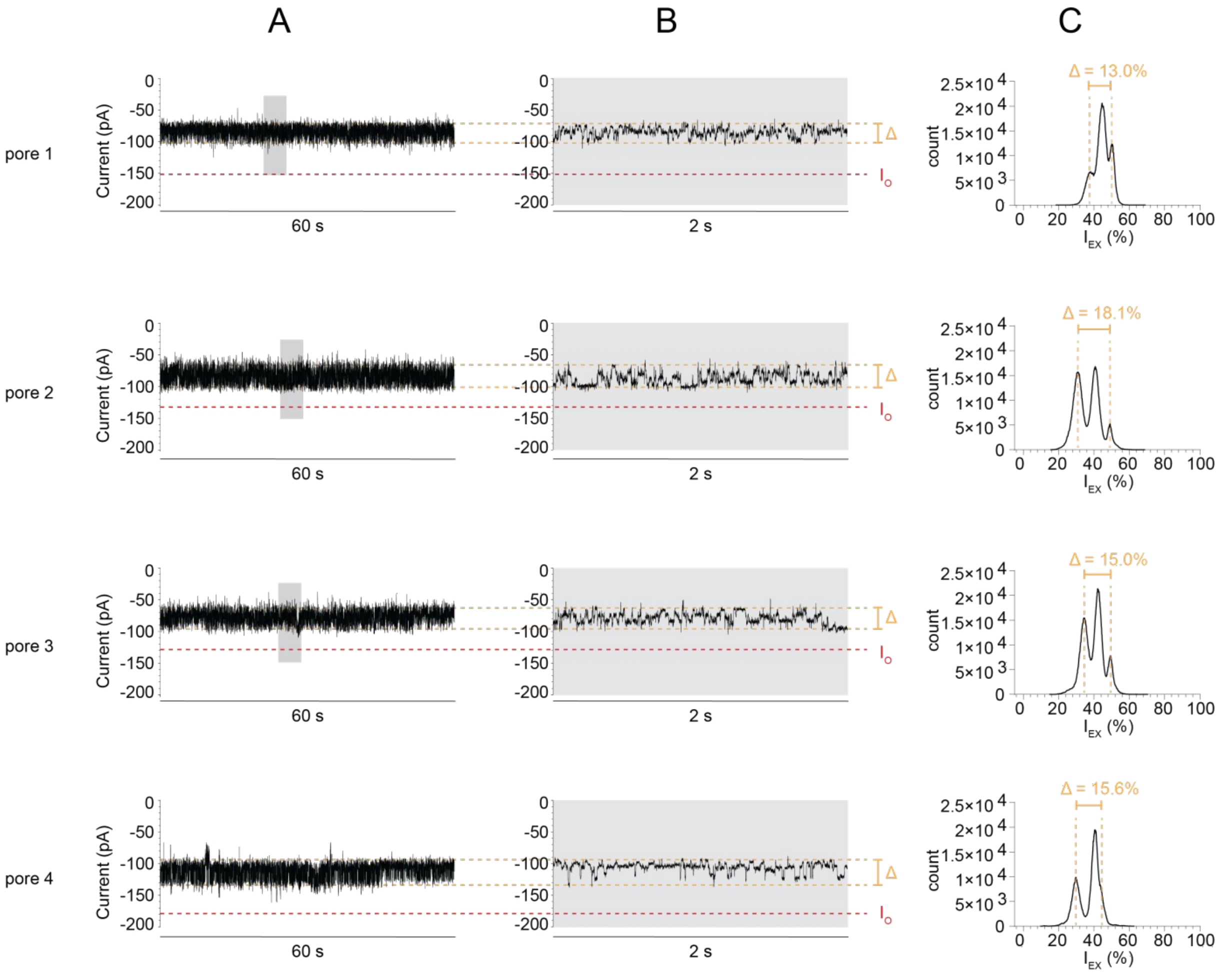

Supporting Figure 13: $YaxA_{\Delta 40}B_{strepII\text{-}70aa}{}^{1.9^*/2.3^*/2.6^*}$ (70 aa) caging SA. A) Four representative electrophysiology traces (60 s) of SA bound to a $YaxA_{\Delta 40}B_{strepII\text{-}70aa}{}^{1.9^*/2.3^*/2.6^*}$ pore. I_O is indicated by red dotted line. Outer blockade-boundaries are indicated by orange dotted line, spanning a bandwidth indicated by Δ. **B)** Zoom-in traces (2 s) of the corresponding section in panel A. **C)** All-point histograms (60 s) of the trace in showed in panel A, showing the delta between outer I_{EX}-boundaries (ΔI_{EX}, orange). $\Delta I_{EX,70aa}$ = 15.42 ± 1.83 Δ% (N=4, error bars represent standard deviation). 20 nM SA was added to *cis*. 1.9*, 2.3*, 2.6* nS pores were used. Measurements were conducted at –75 mV, at 150 mM NaCl, 15 mM TrisHCl pH 7.5, with DPhPC lipids composing the bilayer. Data were recorded at 50 kHz sampling rate, and 10 kHz Bessel filter. Traces were additionally filtered with 500 Hz low-pass Gaussian filter for all-point histogram (0.1 pA bin size) and for visualization. Inhouse MATLAB script was used to calculate I_{EX} of outer peaks and the ΔI_{EX}.

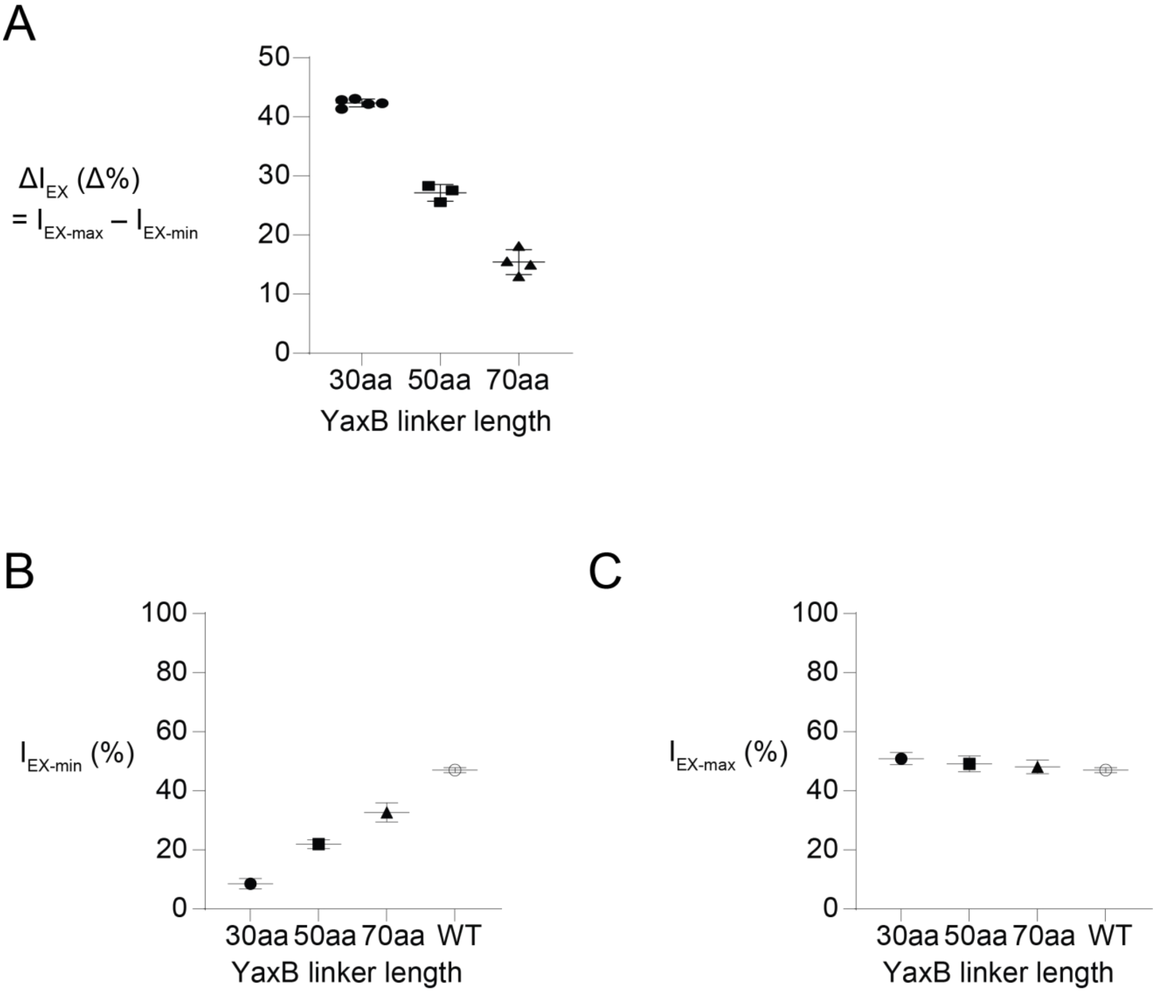

Supporting Figure 14: Length dependence of I_{EX}-values of the multilevel SA-signal of $YaxA_{\Delta 40}B^{1.9*/2.3*/2.6*}$ linker constructs. A) The bandwidth of multilevel (ΔI_{EX}) decreased; **B)** the lower boundary ($I_{EX\text{-}min}$) increased; and **C)** and the higher boundary ($I_{EX\text{-}max}$) remained stable over linker length. $YaxA_{\Delta 40}B_{WT}^{2.3*}$ was used, and for the $YaxA_{\Delta 40}B^{1.9*/2.3*/2.6*}$ linker constructs all 1.9*, 2.3*, 2.6* nS pores were used. Error bars represent standard deviation.

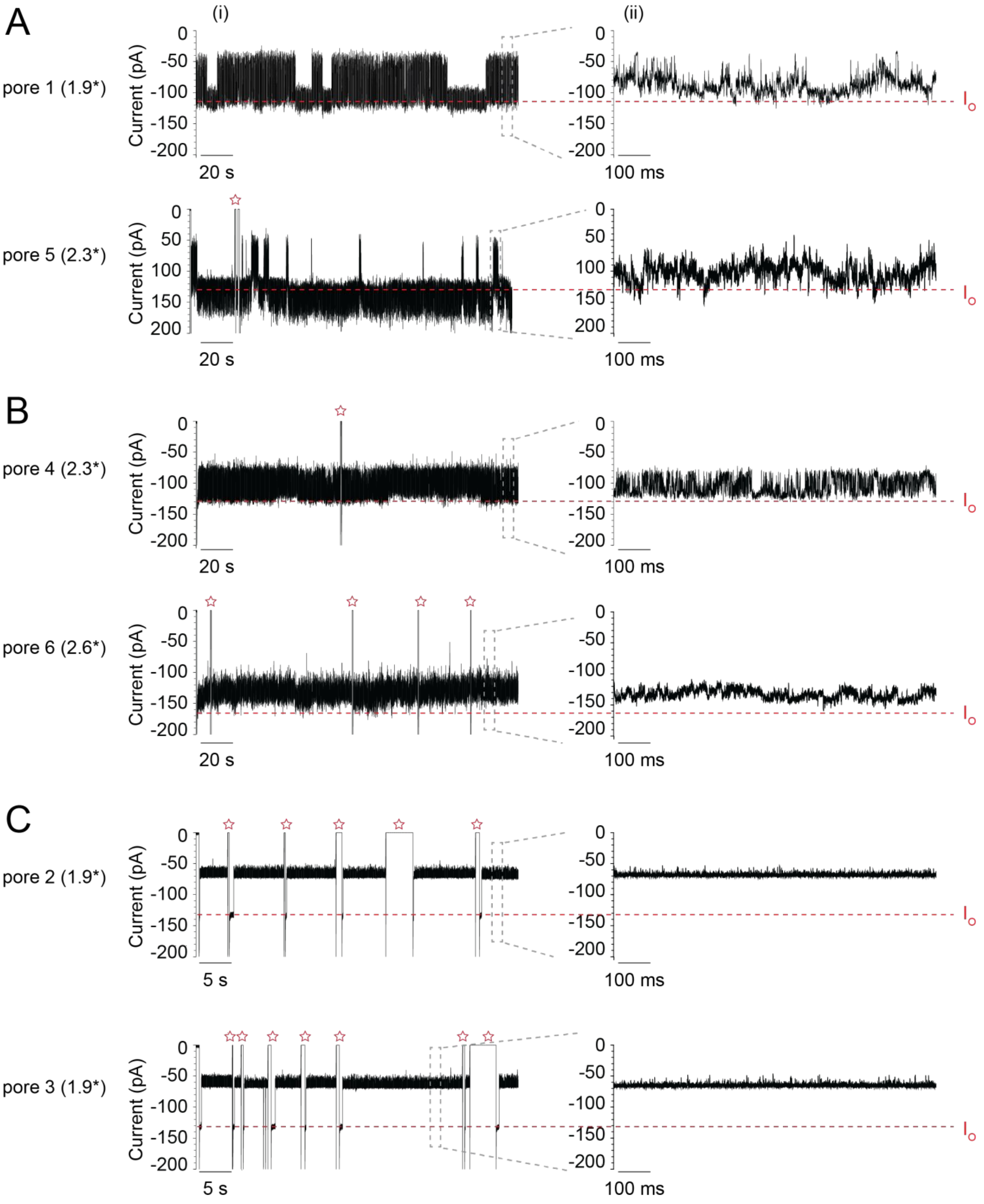

Supporting Figure 15: YaxA$_{\Delta 40}$B$_{strepII\text{-}100aa}$$^{1.9*/2.3*/2.6*}$ capturing SA. Selected electrophysiology traces of SA captured by YaxA$_{\Delta 40}$B$_{strepII\text{-}100aa}$$^{1.9*/2.3*/2.6*}$. Red stars indicate flipping of potential (+/– 75 mV). I_O is indicated by red dotted line. Panels **A-C** show typical observations. SA was added to *cis* at 20 nM. Measurements were conducted at –75 mV, in 150 mM NaCl, 15 mM TrisHCl pH 7.5, with DPhPC lipids composing the bilayer. Data were recorded at 50 kHz sampling rate, and 10 kHz Bessel filter. Traces were additionally filtered with 2 kHz low-pass Gaussian filter for visualization.

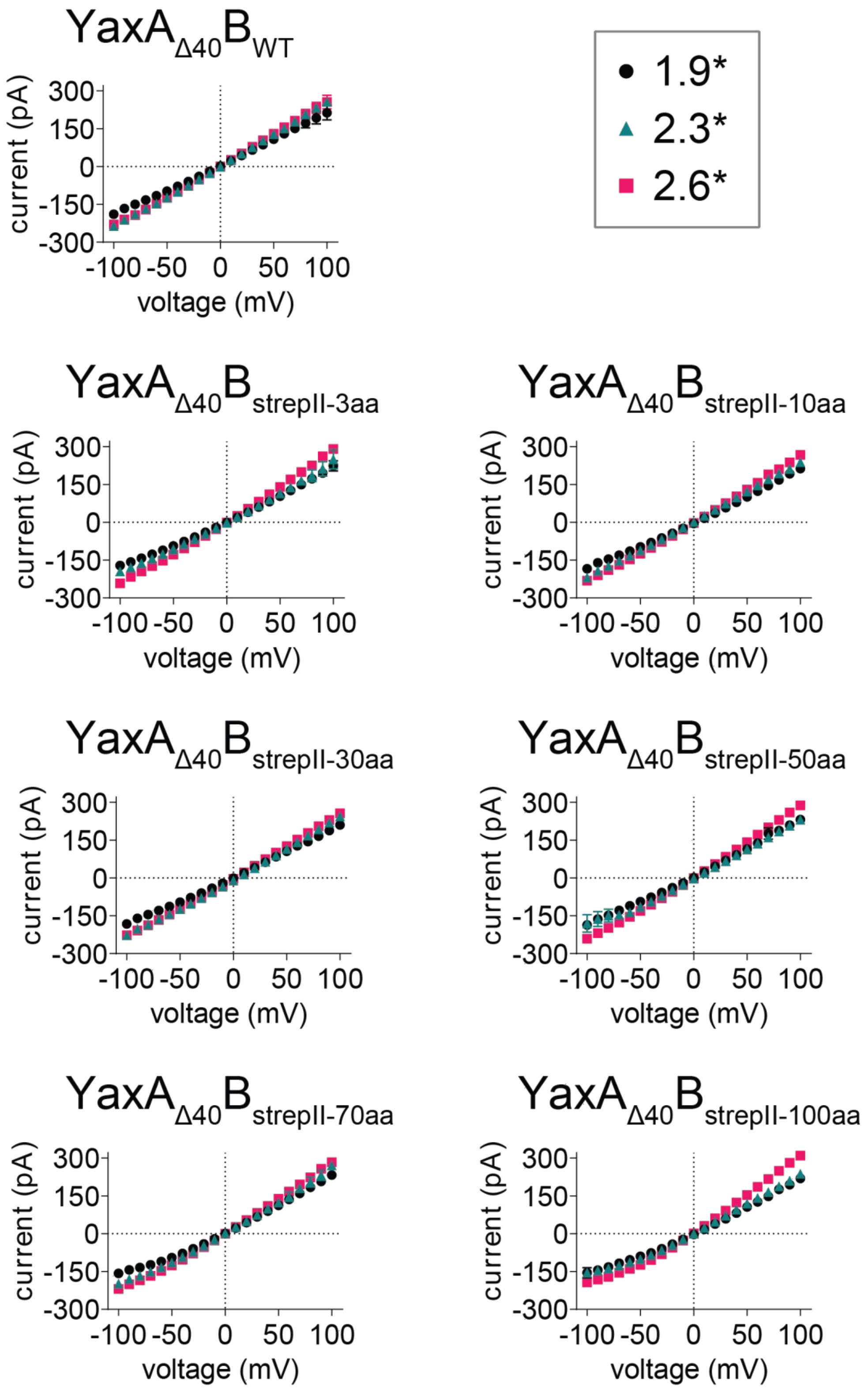

Supporting Figure 16: I/V curves of YaxA$_{\Delta40}$B$_{strepII\text{-}linker}$ constructs. Current against voltage (I/V)-curves of the YaxA$_{\Delta40}$B$_{strepII\text{-}linker}$ constructs in this work. Pores of conductance 1.94 ± 0.09 nS (1.9* nanopores; at –35 mV in 150 mM NaCl; black), 2.29 ± 0.23 nS (2.3* nanopores; teal) and 2.63 ± 0.09 nS (2.6* nanopores; pink) are indicated in legend. At least N=3 pores were included per data point, error bars indicate standard deviation. I/V curves were conducted at 150 mM NaCl, 15 mM TrisHCl pH 7.5, with DPhPC lipids composing the bilayer. Data were recorded at 50 kHz sampling rate, and 10 kHz or 5 kHz Bessel filter.

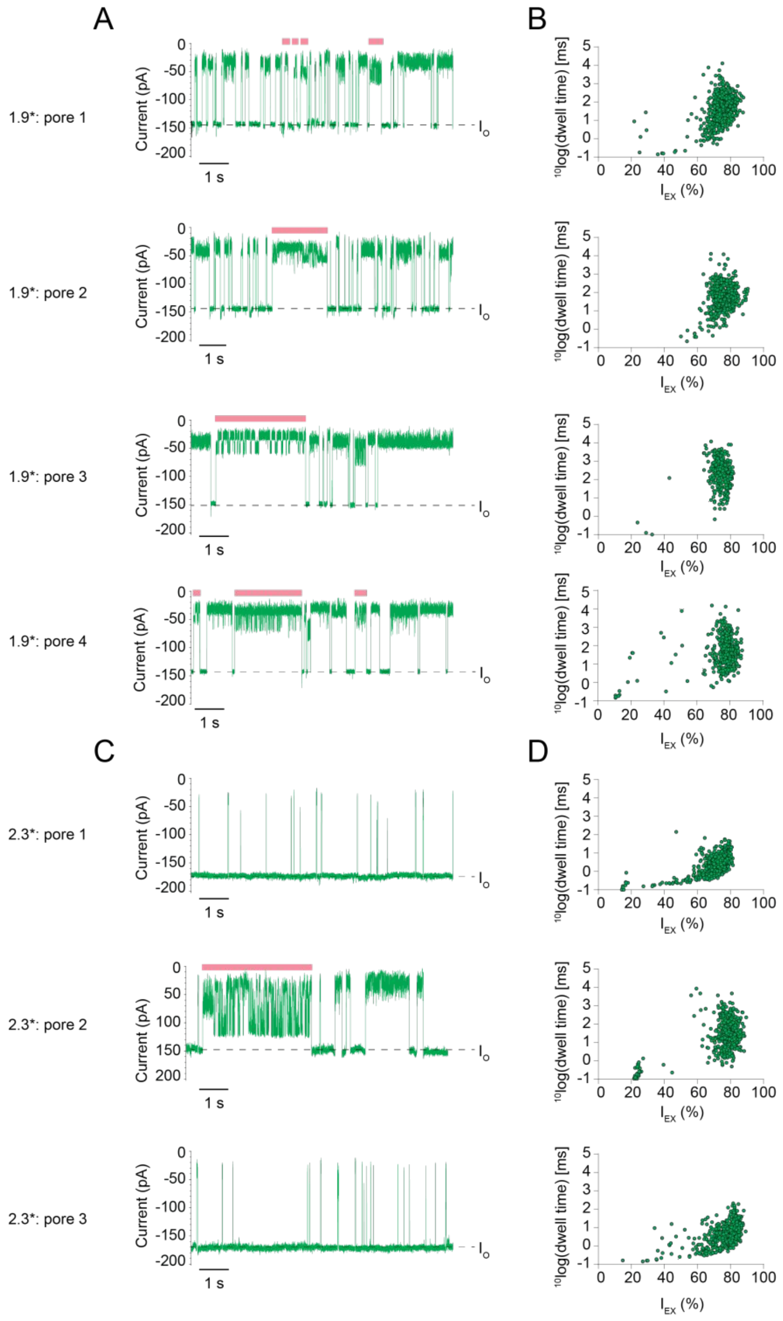

Supporting Figure 17: BT capture by YaxA$_{\Delta40}$B$_{IS20\text{-}70aa}$ 1.9* and 2.3* nS pores. A) Four representative electrophysiology traces of YaxA$_{\Delta40}$B$_{IS20\text{-}70aa}$$^{1.9*}$ nanopores capturing BT (green). **B)** Corresponding scatterplots of single BT events in panel A, of at least n=450 events per replicate (~3 mins of recording). **C)** Three representative electrophysiology traces of YaxA$_{\Delta40}$B$_{IS20\text{-}70aa}$$^{2.3*}$ nanopores capturing BT (green). **D)** Corresponding scatterplots of single BT events in panel C, of at least n=450 events per replicate (~3 mins of recording). IS20-BT binding- and unbinding interaction is indicated with pink squares. I_O is indicated by black dotted line. 20 nM BT was added to *cis* for 2.3* pores, and 50 nM BT was added to *cis* for 1.9* pores. Measurements were conducted at –75 mV, at 150 mM NaCl, 15 mM TrisHCl pH 7.5, with DPhPC lipids composing the bilayer. Data were recorded at 50 kHz sampling rate, and 10 kHz Bessel filter. Traces were additionally filtered with 2 kHz low-pass Gaussian filter for visualization.

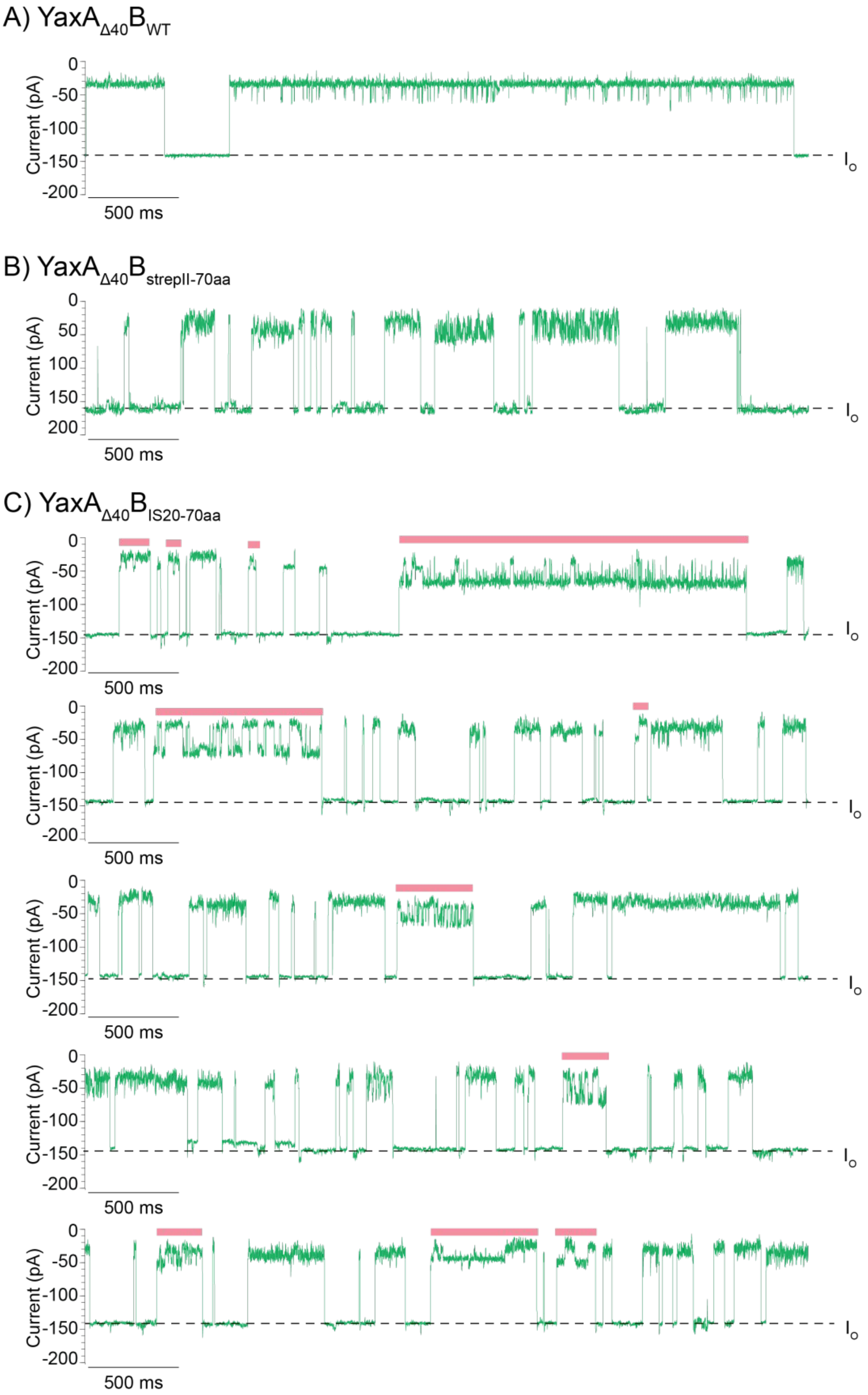

Supporting Figure 18: BT capture by YaxA$_{\Delta40}$B$_{IS20\text{-}70aa}$$^{1.9*}$ and control constructs. A) YaxA$_{\Delta40}$B$_{WT}$$^{1.9*}$ capturing BT (20 nM *cis*, –75 mV) **. B)** YaxA$_{\Delta40}$B$_{strepII\text{-}70aa}$$^{2.3*}$ capturing BT (50 nM in *trans*, +75 mV). **C)** YaxA$_{\Delta40}$B$_{IS20\text{-}70aa}$$^{1.9*}$ capturing BT (50 nM in *cis*, –75 mV). IS20-BT binding- and unbinding interaction is indicated with pink squares. I_O is indicated by black dotted line. Measurements were conducted in 150 mM NaCl, 15 mM TrisHCl pH 7.5, with DPhPC lipids composing the bilayer. Data were recorded at 50 kHz sampling rate, and 10 kHz Bessel filter. Traces were additionally filtered with 1 kHz low-pass Gaussian filter for visualization.

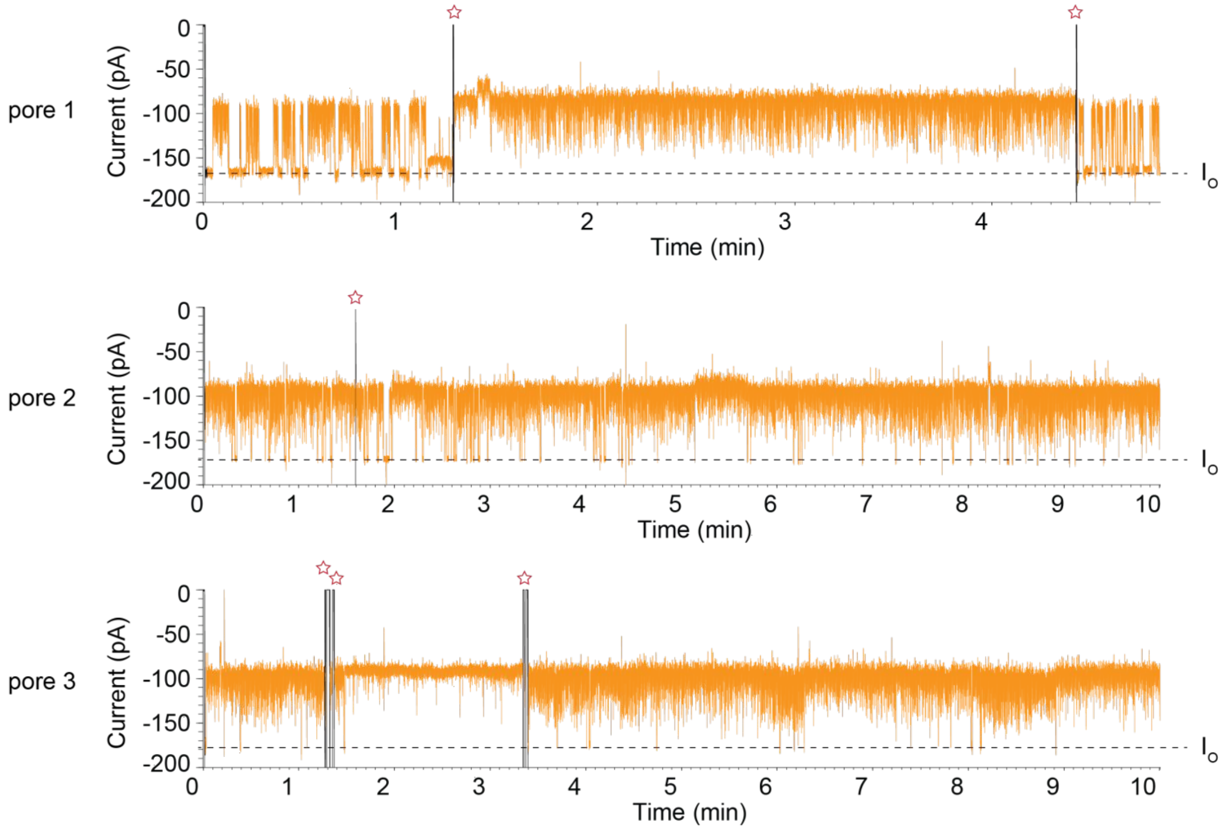

Supporting Figure 19: YaxA$_{\Delta40}$B$_{weak\text{-}50aa}$$^{2.3*/2.6*}$ capturing SA. Three representative electrophysiology traces of at least 4 min showing the capture of SA (orange) in YaxA$_{\Delta40}$B$_{weak\text{-}50aa}$$^{2.3*/2.6*}$. I_O indicated by black dotted line. Flipping of potential (+/– 75 mV) indicated by red star. 20 nM SA was added to *cis*. 2.3* and 2.6* nS pores were used. Measurements were conducted at –75 mV, at 150 mM NaCl, 15 mM TrisHCl pH 7.5, with DPhPC lipids composing the bilayer. Data were recorded at 50 kHz sampling rate, and 10 kHz Bessel filter. Traces were additionally filtered with 500 Hz low-pass Gaussian filter for visualization.

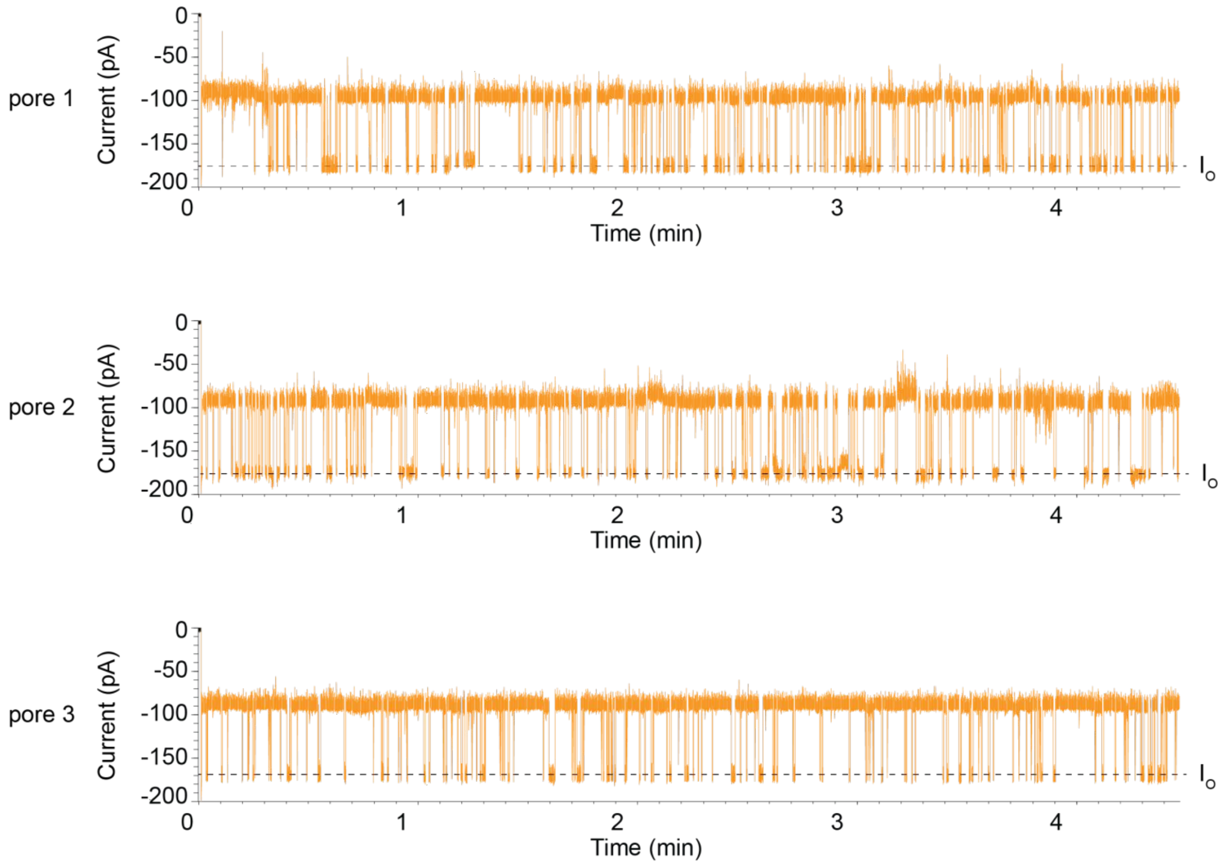

Supporting Figure 20: YaxA$_{\Delta 40}$B$_{58aa}$$^{2.3*}$ capturing SA. Three representative electrophysiology traces of at least 4 min showing the capture of SA (orange) in a YaxA$_{\Delta 40}$B$_{58aa}$$^{2.3*}$ pore. I_O indicated by black dotted line. 20 nM SA was added to *cis*. Only 2.3* nS pores were used. Measurements were conducted at –75 mV, at 150 mM NaCl, 15 mM TrisHCl pH 7.5, with DPhPC lipids composing the bilayer. Data were recorded at 50 kHz sampling rate, and 10 kHz Bessel filter. Traces were additionally filtered with 2 kHz low-pass Gaussian filter for visualization.

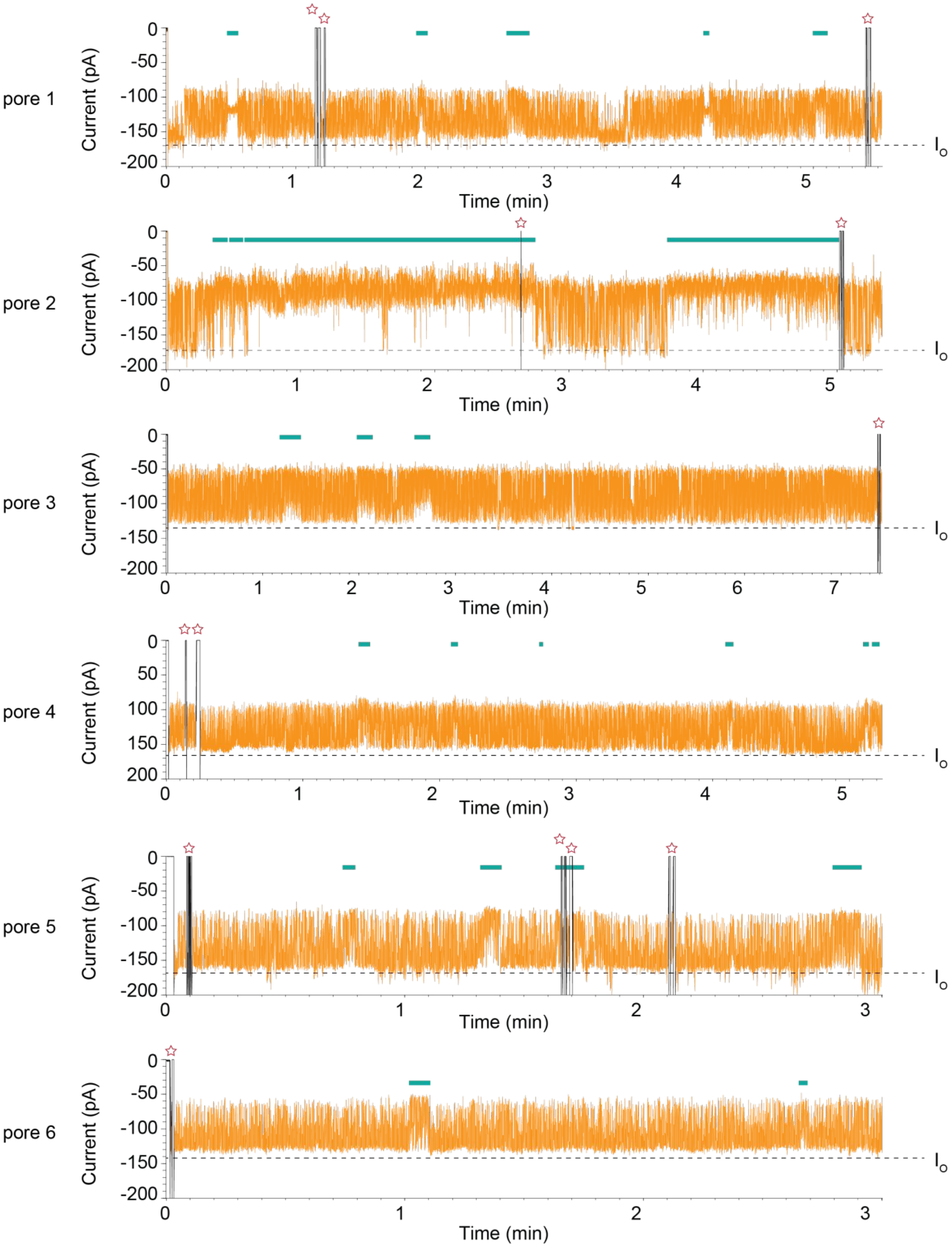

Supporting Figure 21: Macroscale SA caging by $YaxA_{\Delta40}B_{strepII\text{-}EAAAK}{}^{1.9*/2.3*}$. Six representative electrophysiology traces of at least 3 min showing the binding of SA (orange) to $YaxA_{\Delta40}B_{strepII\text{-}EAAAK}{}^{1.9*/2.3*}$. I_O indicated by black dotted line. Flipping of potential (+/– 75 mV) indicated by red star. Teal squares indicate macrostates in the multilevel. 20 nM SA was added to *cis*. 1.9* and 2.3* nS pores were used. Measurements were conducted at –75 mV, at 150 mM NaCl, 15 mM TrisHCl pH 7.5, with DPhPC lipids composing the bilayer. Data were recorded at 50 kHz sampling rate, and 10 kHz Bessel filter. Traces were additionally filtered with 500 Hz low-pass Gaussian filter for visualization.

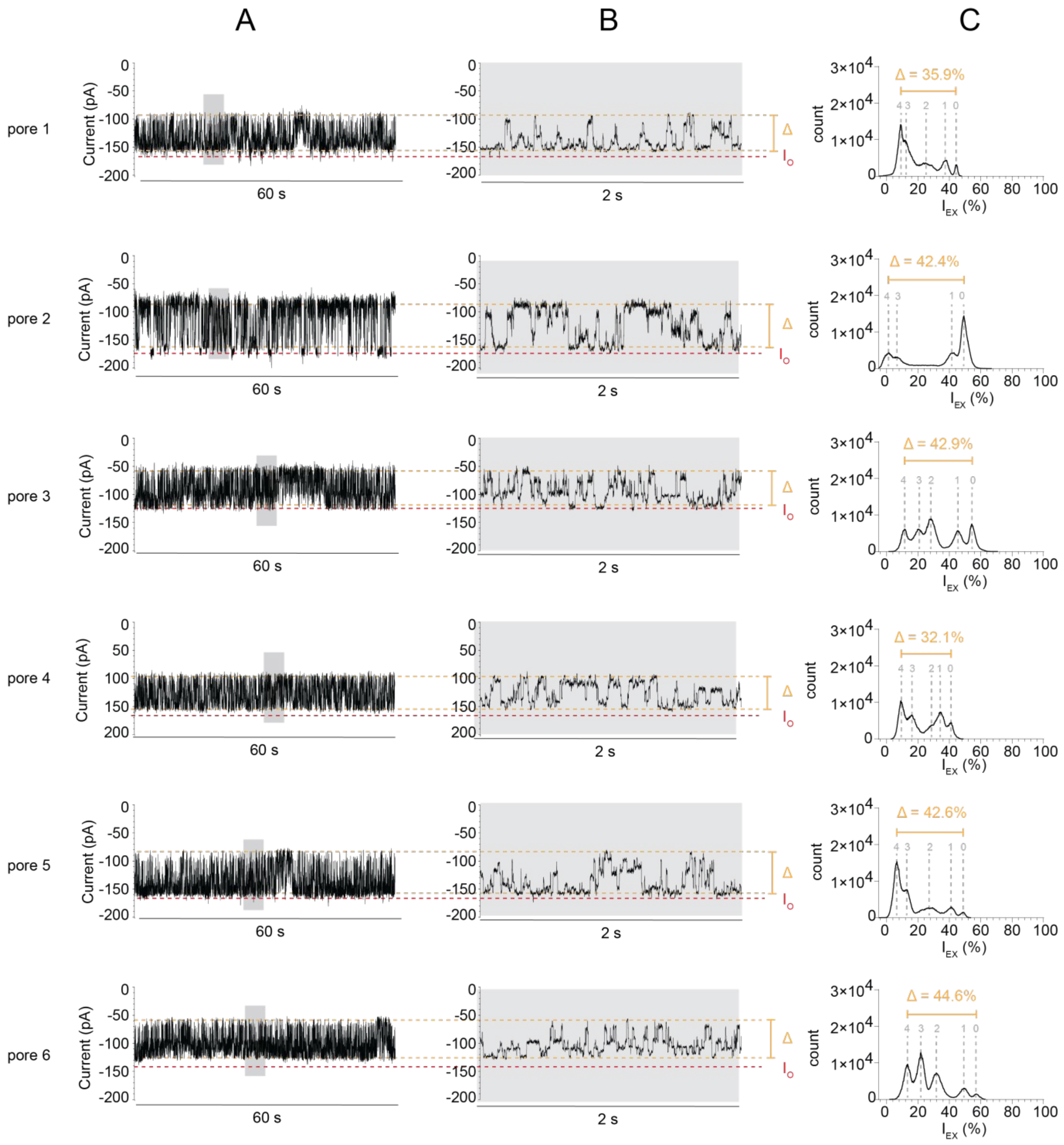

Supporting Figure 22: YaxA$_{\Delta40}$B$_{strepII\text{-}EAAAK}$$^{1.9*/2.3*}$ caging SA. A) Six representative electrophysiology traces (60 s) of SA-bound to a YaxA$_{\Delta40}$B$_{strepII\text{-}EAAAK}$$^{1.9*/2.3*}$ pore. I_O is indicated by red dotted line. Outer blockade-boundaries are indicated by orange dotted line, spanning a bandwidth indicated by Δ. **B)** Zoom-in traces (2 s) of the corresponding section in panel A. **C)** All-point histograms (60 s) of the trace in showed in panel A, showing the delta between outer I_{EX}-boundaries (ΔI_{EX}, orange). Peaks in histogram are indicated with number of linkers likely being bound to SA (1 to 4, grey dotted lines), with the last peak being unbound SA (0 tags). The $\Delta I_{EX,\ EAAAK}$ = 40.08 ± 4.50 Δ% (N=6, error bars represent standard deviation). 20 nM SA was added to *cis*. 1.9* and 2.3* nS pores were used. Measurements were conducted at –75 mV, at 150 mM NaCl, 15 mM TrisHCl pH 7.5, with DPhPC lipids composing the bilayer. Data were recorded at 50 kHz sampling rate, and 10 kHz Bessel filter. Traces were additionally filtered with 500 Hz low-pass Gaussian filter for all-point histogram (0.1 pA bin size) and for visualization. Inhouse MATLAB script was used to calculate I_{EX} of outer peaks and the ΔI_{EX}.

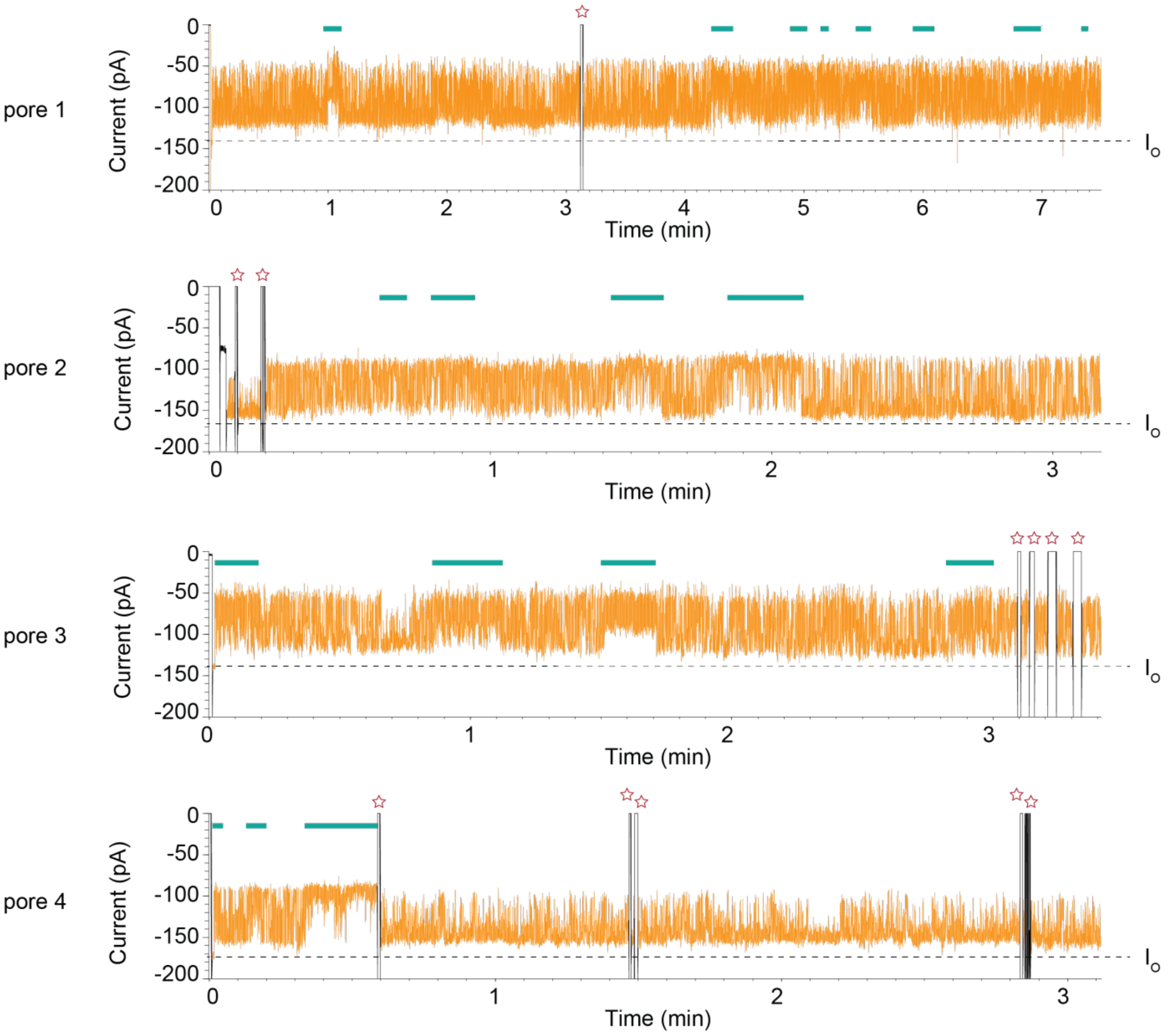

Supporting Figure 23: Macroscale SA caging by YaxA$_{\Delta 40}$B$_{strepII\text{-}AP}$$^{1.9*/2.3*}$. Four representative electrophysiology traces of at least 3 min showing the binding of SA (orange) to YaxA$_{\Delta 40}$B$_{strepII\text{-}AP}$$^{1.9*/2.3*}$. I_O indicated by black dotted line. Flipping of potential (+/– 75 mV) indicated by red star. Teal squares indicate macrostates in the multilevel. 20 nM SA was added to *cis*. 1.9* and 2.3* nS pores were used. Measurements were conducted at –75 mV, at 150 mM NaCl, 15 mM TrisHCl pH 7.5, with DPhPC lipids composing the bilayer. Data were recorded at 50 kHz sampling rate, and 10 kHz Bessel filter. Traces were additionally filtered with 500 Hz low-pass Gaussian filter and for visualization.

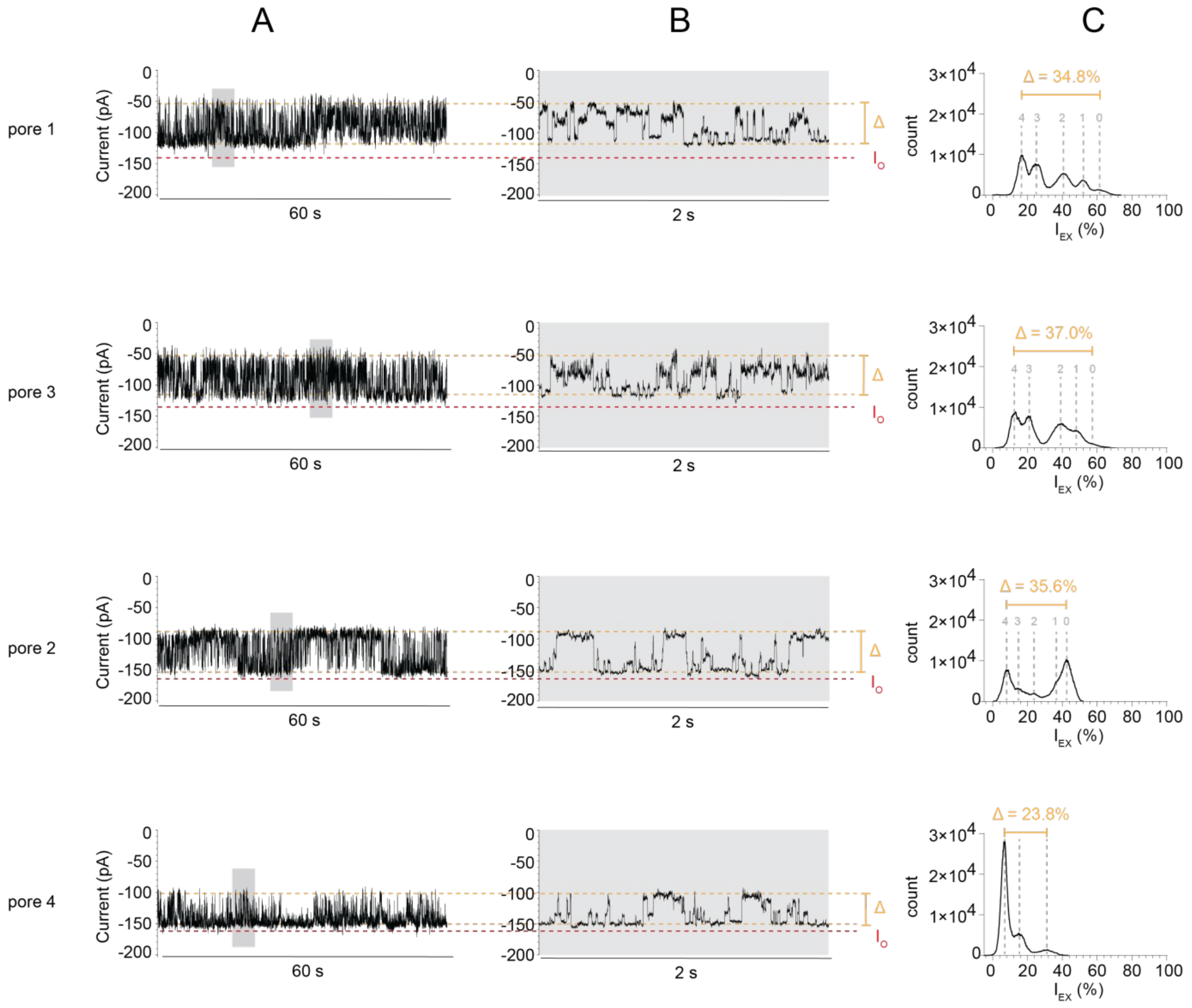

Supporting Figure 24: YaxA$_{\Delta40}$B$_{\text{strepII-AP}}$$^{1.9*/2.3*}$ caging SA. A) Four representative electrophysiology traces (60 s) of SA-bound to a YaxA$_{\Delta40}$B$_{\text{strepII-AP}}$$^{1.9*/2.3*}$ pore. I_O is indicated by red dotted line. Outer blockade-boundaries are indicated by orange dotted line, spanning a bandwidth indicated by Δ. **B)** Zoom-in traces (2 s) of the corresponding section in panel A. **C)** All-point histograms (60 s) of the trace in showed in panel A, showing the delta between outer I_{EX}-boundaries (ΔI_{EX}, orange). Peaks in histogram are indicated with number of linkers likely being bound to SA (1 to 4, grey dotted lines), with the last peak being unbound SA (0 tags). The $\Delta I_{EX,\ AP}$ = 34.80 ± 7.23 Δ% (N=4, error bars represent standard deviation). 20 nM SA was added to *cis*. 1.9* and 2.3* nS pores were used. Measurements were conducted at –75 mV, at 150 mM NaCl, 15 mM TrisHCl pH 7.5, with DPhPC lipids composing the bilayer. Data were recorded at 50 kHz sampling rate, and 10 kHz Bessel filter. Traces were additionally filtered with 500 Hz low-pass Gaussian filter for all-point histogram (0.1 pA bin size) and for visualization. Inhouse MATLAB script was used to calculate I_{EX} of outer peaks and the ΔI_{EX}.

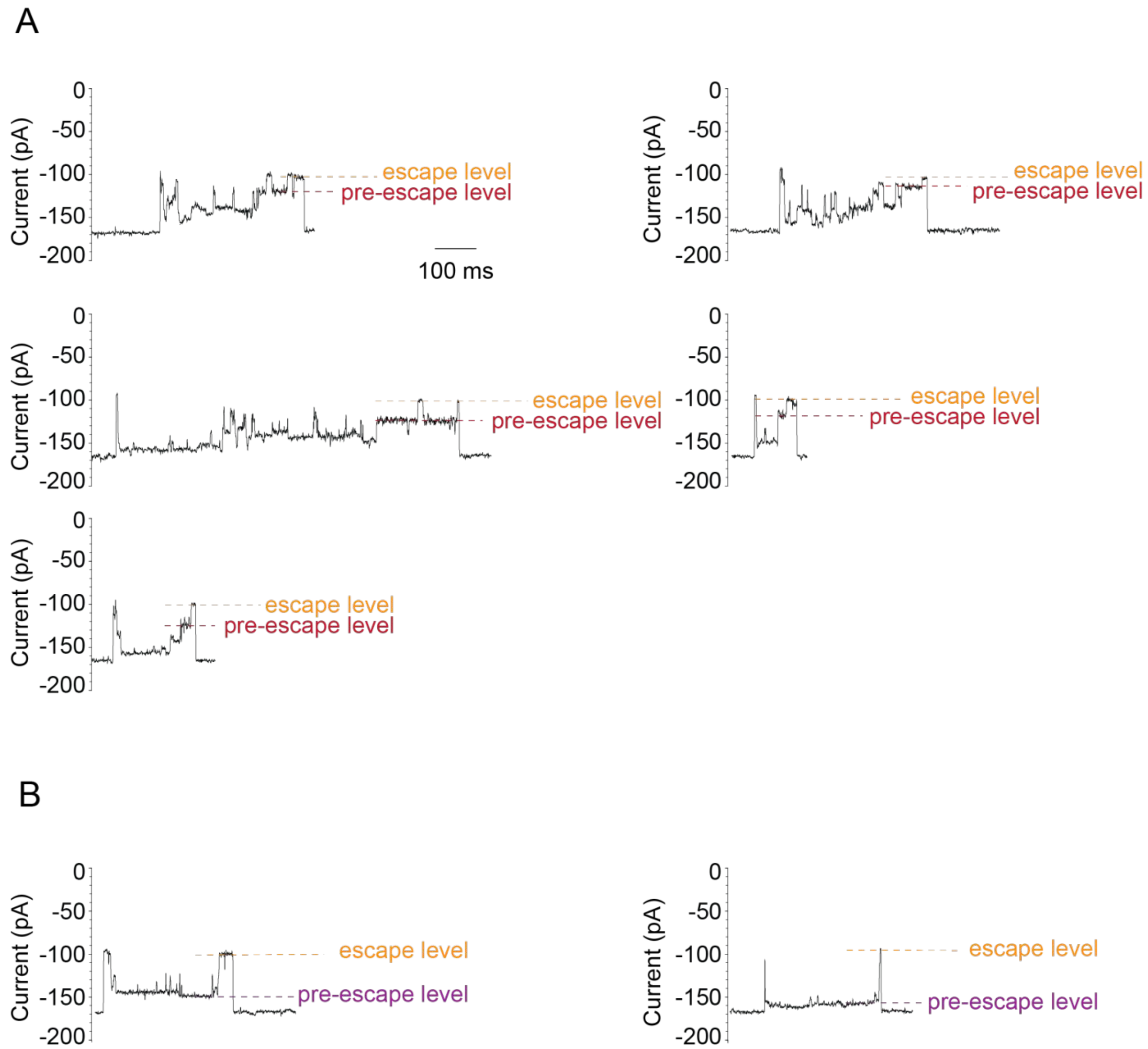

Supporting Figure 25: Biotin-induced escape of SA from $YaxA_{\Delta 40}B_{strepII\text{-}EAAAK}^{2.3*}$ nanopores. Selected multilevel SA (*cis*) blockades detected by $YaxA_{\Delta 40}B_{strepII\text{-}30aa\text{-}EAAAK}^{2.3*}$, with 2 μM biotin (*trans)* competing with strepII-tag for the interaction with SA. The escape level corresponds to the deepest current level, consistent with SA molecules fully occupied by biotin. **A)** Typical events observed for the majority of blockades recorded, whereas the pre-escape level (red) is the second deepest current level observed in SA-blockades. **B)** Examples of the minority of events whereas the release from a higher ΔI_{EX} level (purple). 20 nM SA was added to *cis*; 2 μM biotin was added to *trans*. Measurements were conducted at –75 mV, at 150 mM NaCl, 15 mM TrisHCl pH 7.5, with DPhPC lipids composing the bilayer. Data were recorded at 50 kHz sampling rate, and 10 kHz Bessel filter. Traces were additionally filtered with 500 Hz low-pass Gaussian filter for visualization.

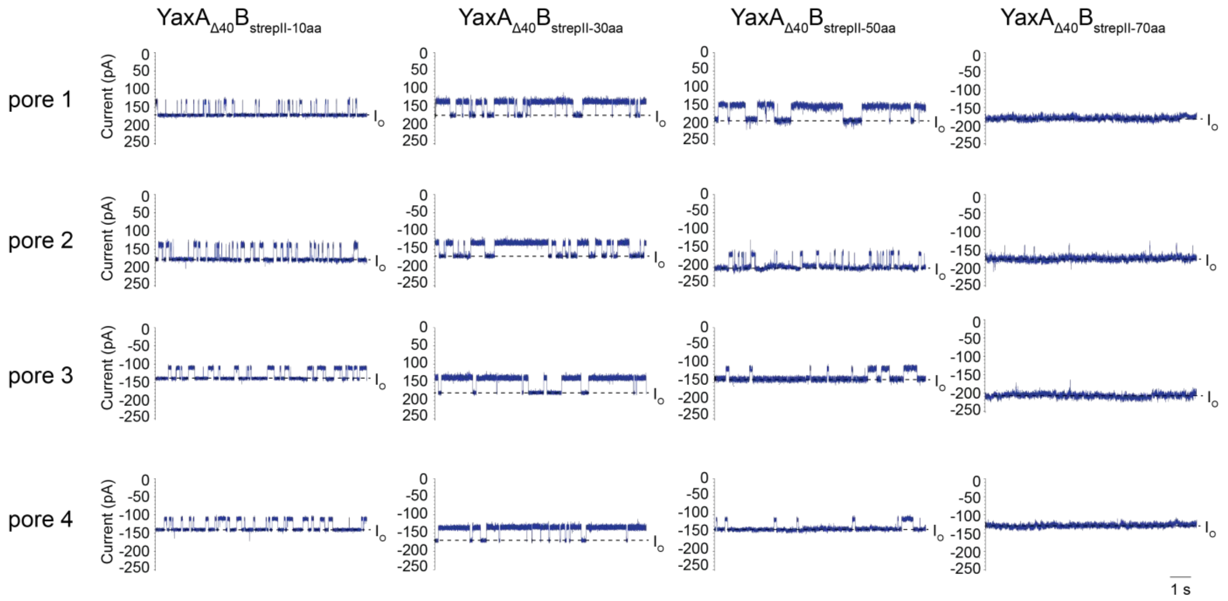

Supporting Figure 26: CRP capture by YaxA$_{\Delta 40}$B$_{\text{strepII-linker}}$$^{1.9*/2.3*/2.6*}$ nanopores. Typical current blockades induced by C-reactive protein (CRP, 125 kDa; blue) showed for a range of YaxA$_{\Delta 40}$B$_{\text{strepII-linker}}$$^{1.9*/2.3*/2.6*}$ (1.9*, 2.3* and 2.6* nS) nanopores (N=4). I_O is indicated by black dotted line. 20 nM CRP was added to *cis*. Measurements were conducted at –75 mV, at 150 mM NaCl, 15 mM TrisHCl pH 7.5, with DPhPC lipids composing the bilayer. Data were recorded at 50 kHz sampling rate, and 10 kHz Bessel filter. Traces were additionally filtered with 2 kHz low-pass Gaussian filter for visualization.

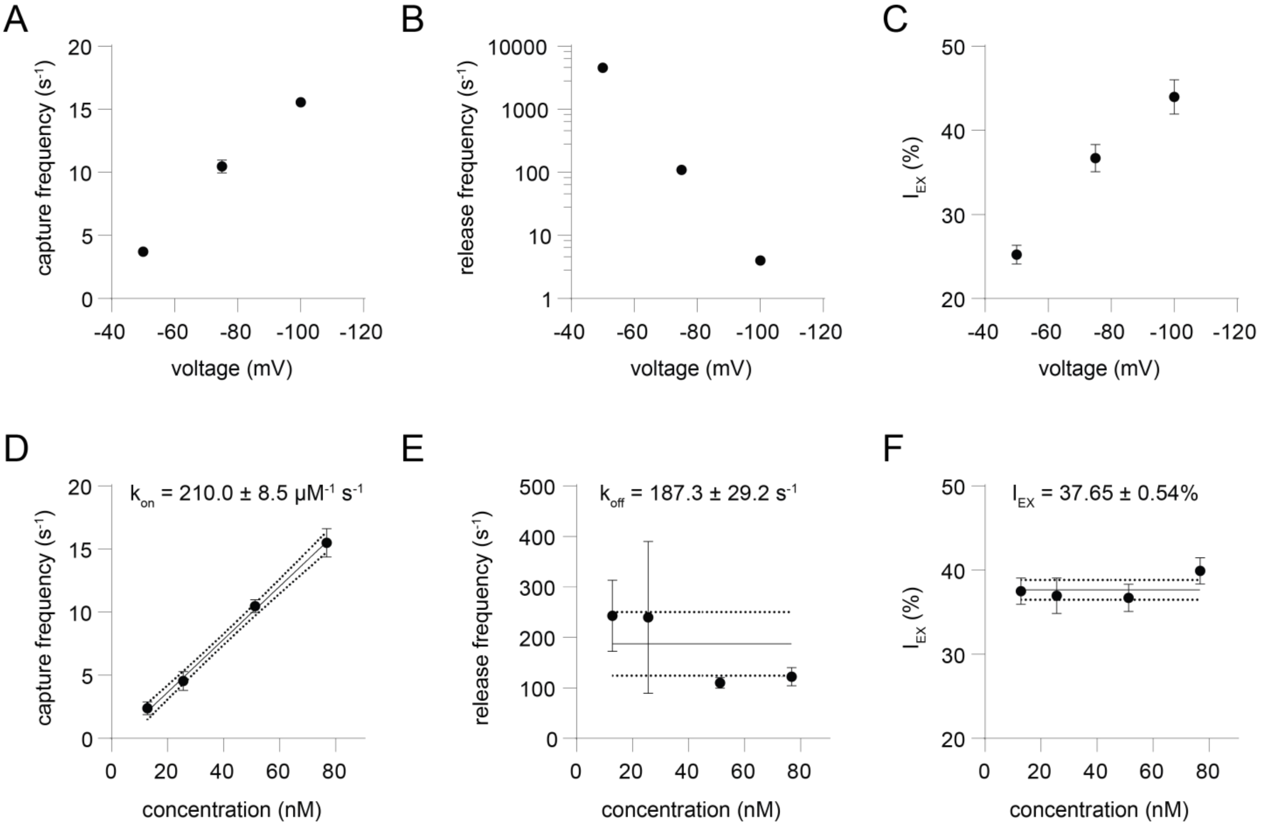

Supporting Figure 27: Characterization of Human Transferrin (HTf) by $YaxA_{\Delta40}B_{WT}^{2.3*}$ nanopores. A-C) Voltage dependency of the capture frequency **(A)**, release frequency **(B)** and I_{EX} **(C)** of HTf (76-81 kDa) blockades in $YaxA_{\Delta40}B_{WT}^{2.3*}$. **D-E)** Concentration dependency of the capture frequency **(D)** capture release **(E)** and I_{EX} **(F)** of Human Transferrin (HTf, 76-81 kDa) blockades. The k_{on} = 210.0 ± 8.5 $\mu M^{-1}s^{-1}$, and k_{off} = 87.3 ± 29.2 s^{-1} and I_{EX} = 62.35 ± 0.54% can be retrieved using least-squared regression analysis (GraphPad Prism 10.2.0). Errors of fitting represent standard error of the mean (SEM), and dotted line represents 95% confidence interval. For each datapoint, at least N=3 pores were measured, with n≥344 events per datapoint (n≥150 events for –100 mV datapoint). Error bars in graphs represent standard deviation (σ) of the mean (μ). Voltage dependency experiments were performed with 51.2 nM HTf in cis. Concentration dependency experiments were performed at –75 mV. Protein concentration was pre-determined with Bradford assay. All measurements were performed at 150 mM NaCl, 15 mM TrisHCl pH 7.5, with DPhPC lipids composing the bilayer. Data were recorded at 50 kHz sampling rate, and 10 kHz Bessel filter.

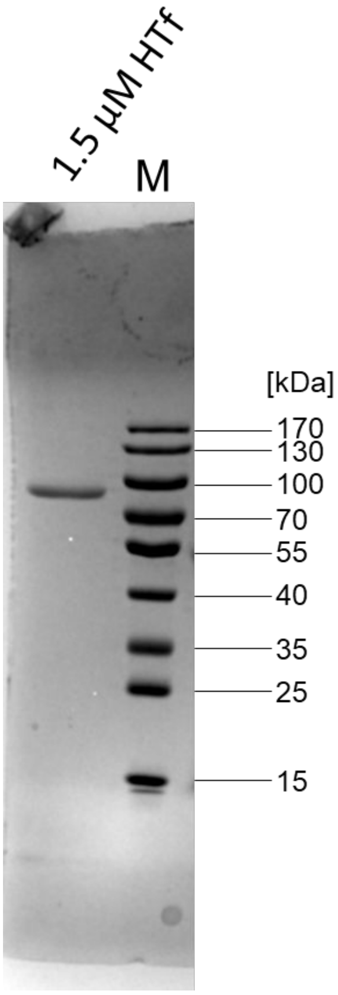

Supporting Figure 28: Human Transferrin (76-81 kDa) on 12% SDS-PAGE gel. Left lane contains 1.5 µM of HTf, right line contains pre-stained protein ladder (M). See ref.[1] for SDS-PAGE gels of CRP, SA, and BT.

A

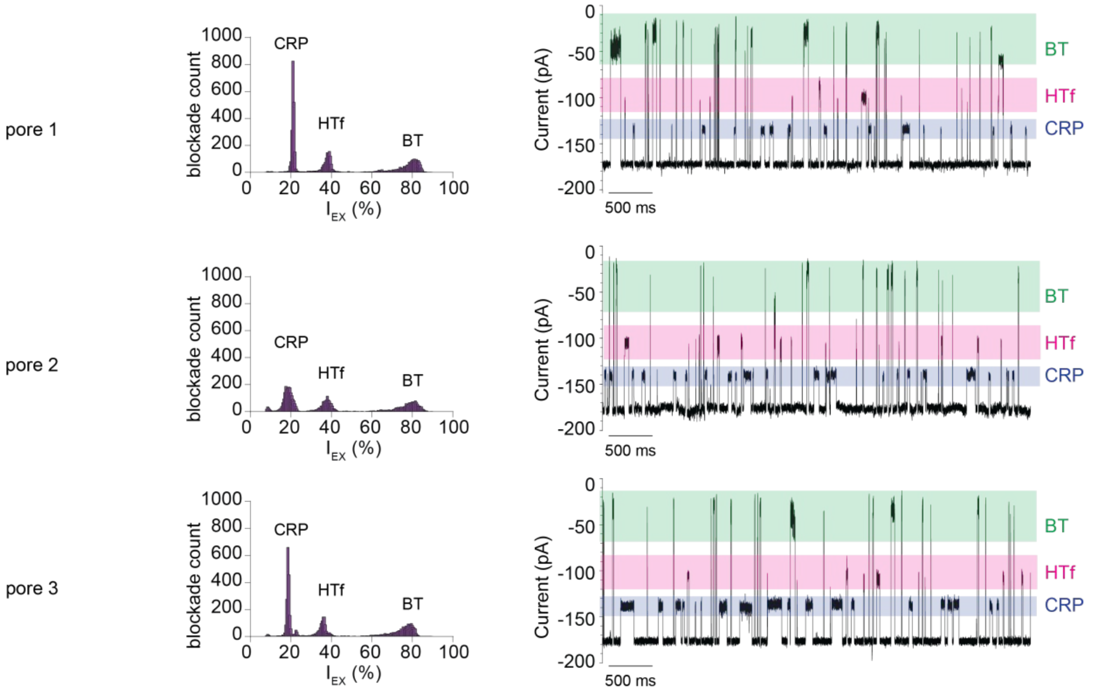

B

WT	Pore 1		Pore 2		Pore 3		N=3
	n=5276		n=4877		n=4927		
	$I_{EX} \pm \sigma$ (%)	AUC	$I_{EX} \pm \sigma$ (%)	AUC	$I_{EX} \pm \sigma$ (%)	AUC	AUC $\mu \pm \sigma$
BT(i)	80.68 ± 2.49	935.51	79.58 ± 3.51	857.54	78.10 ± 2.42	887.37	893.47 ± 32.12
BT(ii)	71.42 ± 6.36		68.18 ± 8.65		69.25 ± 6.07		
HTf	38.24 ± 1.37	481.53	37.31 ± 2.87	575.16	35.86 ± 1.40	404.24	486.98 ± 69.88
CRP	21.09 ± 0.50	968.26	18.64 ± 2.27	1003.43	18.61 ± 0.56	852.91	941.54 ± 64.29

Supporting Figure 29: Aspecific protein capture by $YaxA_{\Delta 40}B_{WT}^{2.3*}$. A) Blockade histogram (left) and representative current traces for the interaction of protein mix (BT:HTf:CRP, 1:1:1, 20 nM final conc. of each in *cis*) with $YaxA_{\Delta 40}B_{WT}^{2.3*}$ nanopores. **B)** $I_{EX} \pm \sigma$ and area under the curve (AUC) calculated with a MATLAB script (script #2) from the current blockades. AUC $\mu \pm \sigma$ were computed in Excel. BT peak in histogram contains two terms (i & ii), therefore the sum of AUC was taken to calculate the mean (μ) and standard deviation (σ) AUC over three replicates. Data were recorded at –75 mV, and the first 519 s were used for analysis. Protein concentration was determined with Bradford assay. Experiment was executed in triplicate (N=3 pores). All measurements were performed at 150 mM NaCl, 15 mM TrisHCl pH 7.5, with DPhPC lipids composing the bilayer. Data were recorded at 50 kHz sampling rate, and 10 kHz Bessel filter. Traces were additionally filtered with 2 kHz low-pass Gaussian filter for visualization.

A

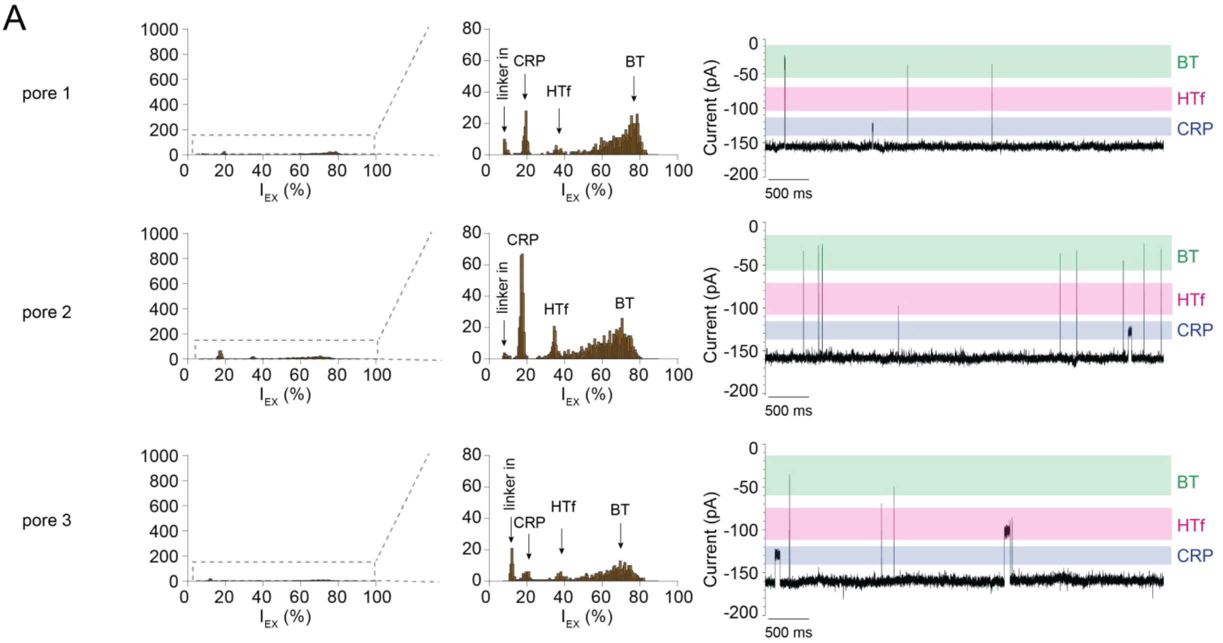

B

70aa	Pore 1		Pore 2		Pore 3		N=3	
	n=732		n=1063		n=492			
	I_{EX} ± σ (%)	AUC	I_{EX} ± σ (%)	AUC	I_{EX} ± σ (%)	AUC	AUC μ ± σ	**AUC fold-change (WT/70aa)**
BT(i)	77.14 ± 2.48	240.87	71.18 ± 3.85	286.40	71.46 ± 4.83	191.14	239.47 ± 38.90	**3.73**
BT(ii)	68.98 ± 4.69		60.77 ± 6.52		59.09 ± 7.67			
HTf	41.87 ± 12.66	43.91	34.55 ± 0.68	30.73	36.59 ± 5.01	27.83	34.16 ± 7.00	**14.26**
CRP	19.26 ± 0.80	44.89	17.24 ± 0.78	126.65	19.72 ± 1.96	26.81	66.12 ± 43.44	**14.24**

Supporting Figure 30: Premixed protein sample capture by $YaxA_{\Delta 40}B_{strepII\text{-}70aa}{}^{2.3*}$ linker construct (N=3 replicates). A) Blockade histogram (left) and representative current traces for the interaction of protein mix (BT:HTf:CRP, 1:1:1, 20 nM final conc. of each in *cis*) with $YaxA_{\Delta 40}B_{strepII\text{-}70aa}{}^{2.3*}$ nanopores. **B)** I_{EX} ± σ and area under the curve (AUC) calculated with a MATLAB script (script #2) from the current blockades. AUC μ ± σ were computed in Excel. BT peak in histogram contains two terms (i & ii), therefore the sum of AUC was taken to calculate the mean (μ) and standard deviation (σ) AUC over three replicates. AUC fold change is calculated by (AUC μ WT)/(AUC μ 70aa), and represents a quantification of the steric hindrance the linkers pose to its respective protein. Data were recorded at –75 mV, and the first 519 s were used for analysis. Protein concentration was determined with Bradford assay. Experiment was executed in triplicate (N=3 pores). All measurements were performed at 150 mM NaCl, 15 mM TrisHCl pH 7.5, with DPhPC lipids composing the bilayer. Data were recorded at 50 kHz sampling rate, and 10 kHz Bessel filter. Traces were additionally filtered with 2 kHz low-pass Gaussian filter for visualization.

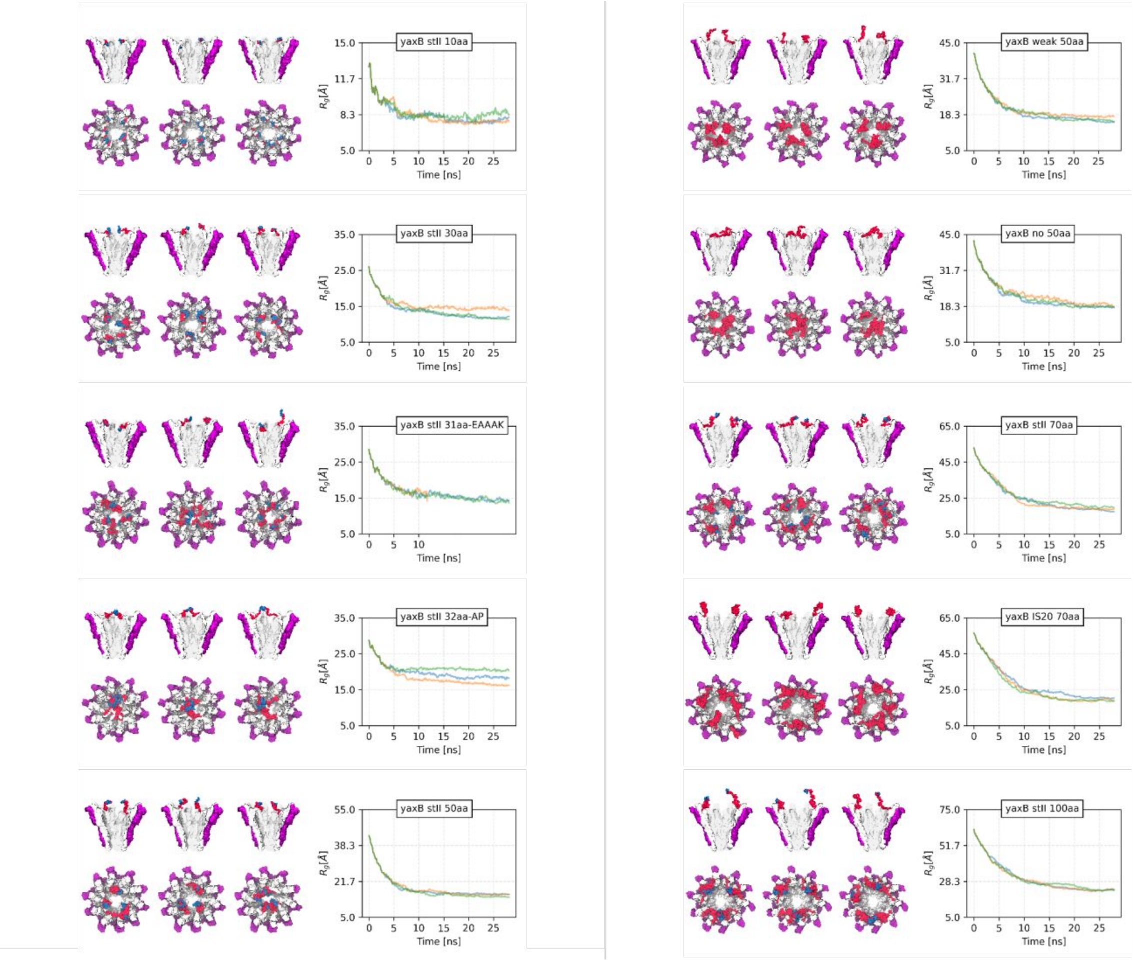

Supporting Figure 31: Implicit-solvent MD simulations of YaxAB linker compaction. Snapshots of the last frame of each production run, for all the systems reported in **Supporting Table 1**, three replicas per system. Right graphs report the mean gyration of radius of the linkers along the simulations. Each coloured line corresponds to a single replica. After ~20 ns, all the systems reached an equilibrium state. For selected systems (30aa, 50aa, 70aa, 100aa), we computed explicit solvent simulations, to better estimate the flexibility of the linkers, see **Supporting Figure 32**. Indeed, implicit solvent would bring to an over-compaction of the disordered domains.[4,5] See also **Supporting Videos 1-5**.

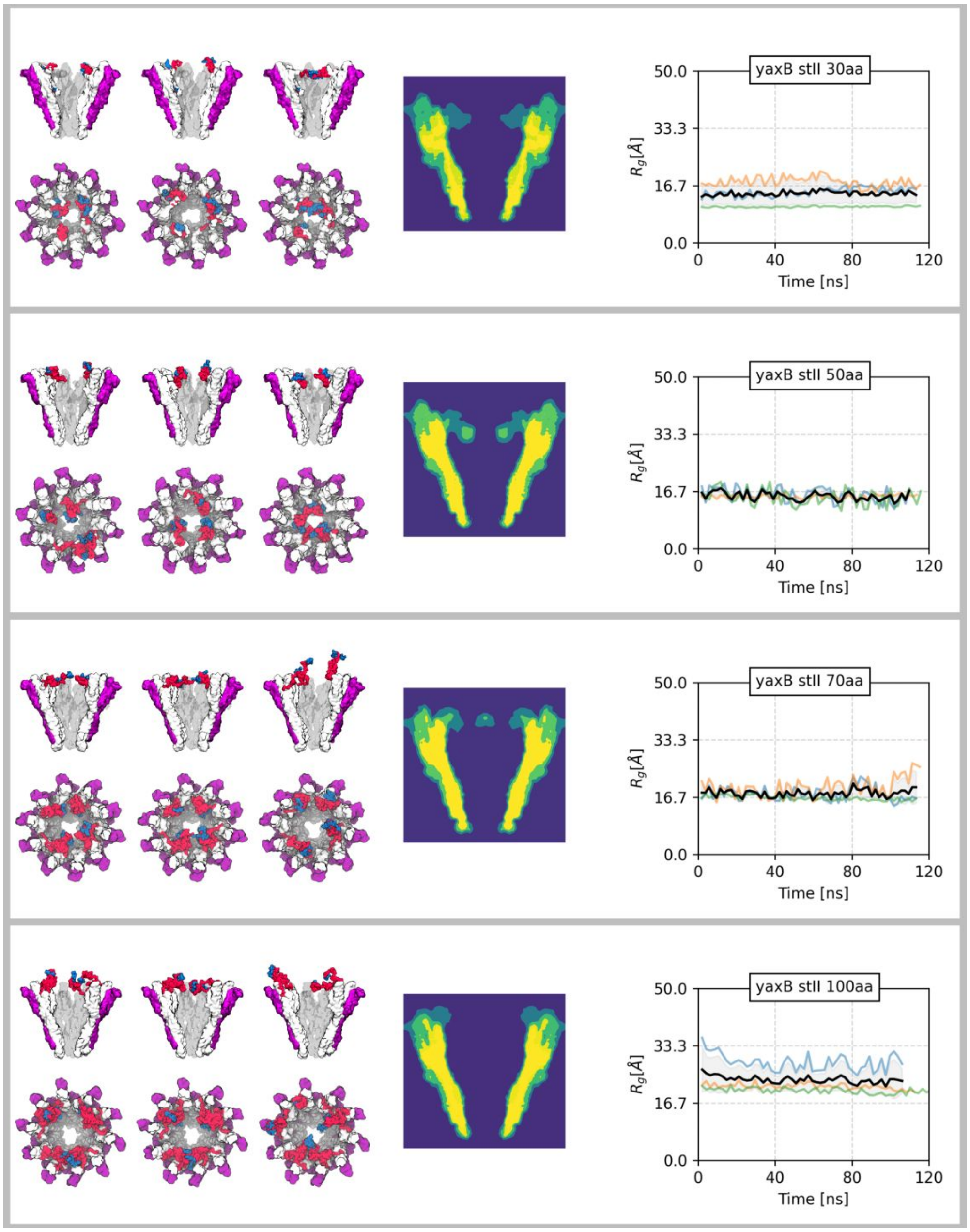

Supporting Figure 32: All-atom MD simulations, explicit solvent. Left column shows the snapshots of the last frame of each production run, for different linkers length, three replicas per system. In purple are represented the YaxA chains, in white the YaxB chains with the linkers in red and the strepII tag in blue. Right column reports the mean gyration of radius of the tails along the simulations. Each coloured line corresponds to a single replica, while in black is reported their average. Conformational changes are extremely slow, as highlighted from the relatively small fluctuation of the radius of gyration of each replica.

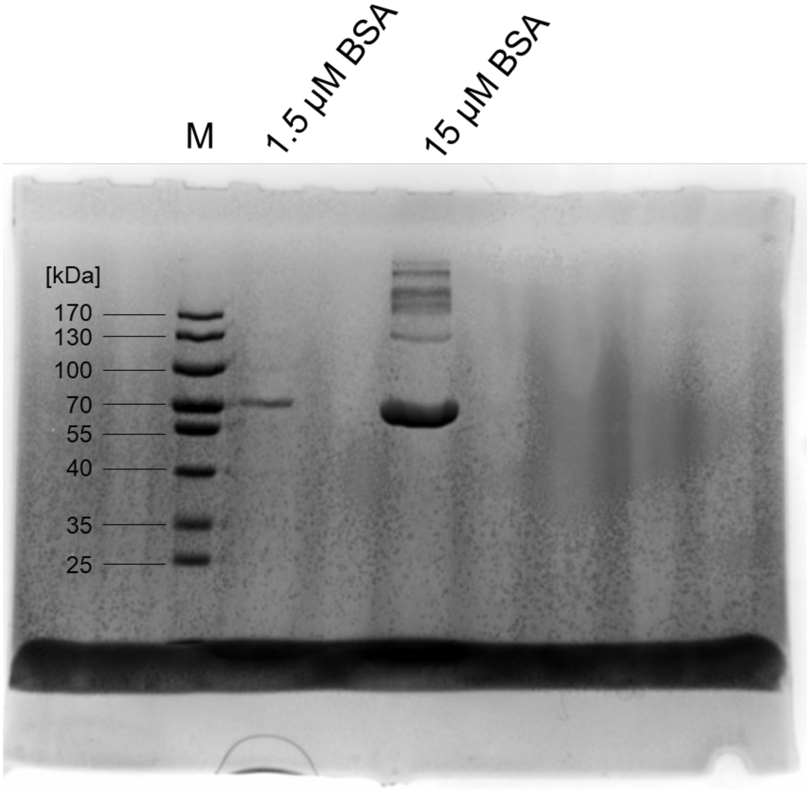

Supporting Figure 33: Bovine Serum Albumin (BSA) on 12% SDS-PAGE gel. M is pre-stained protein ladder.

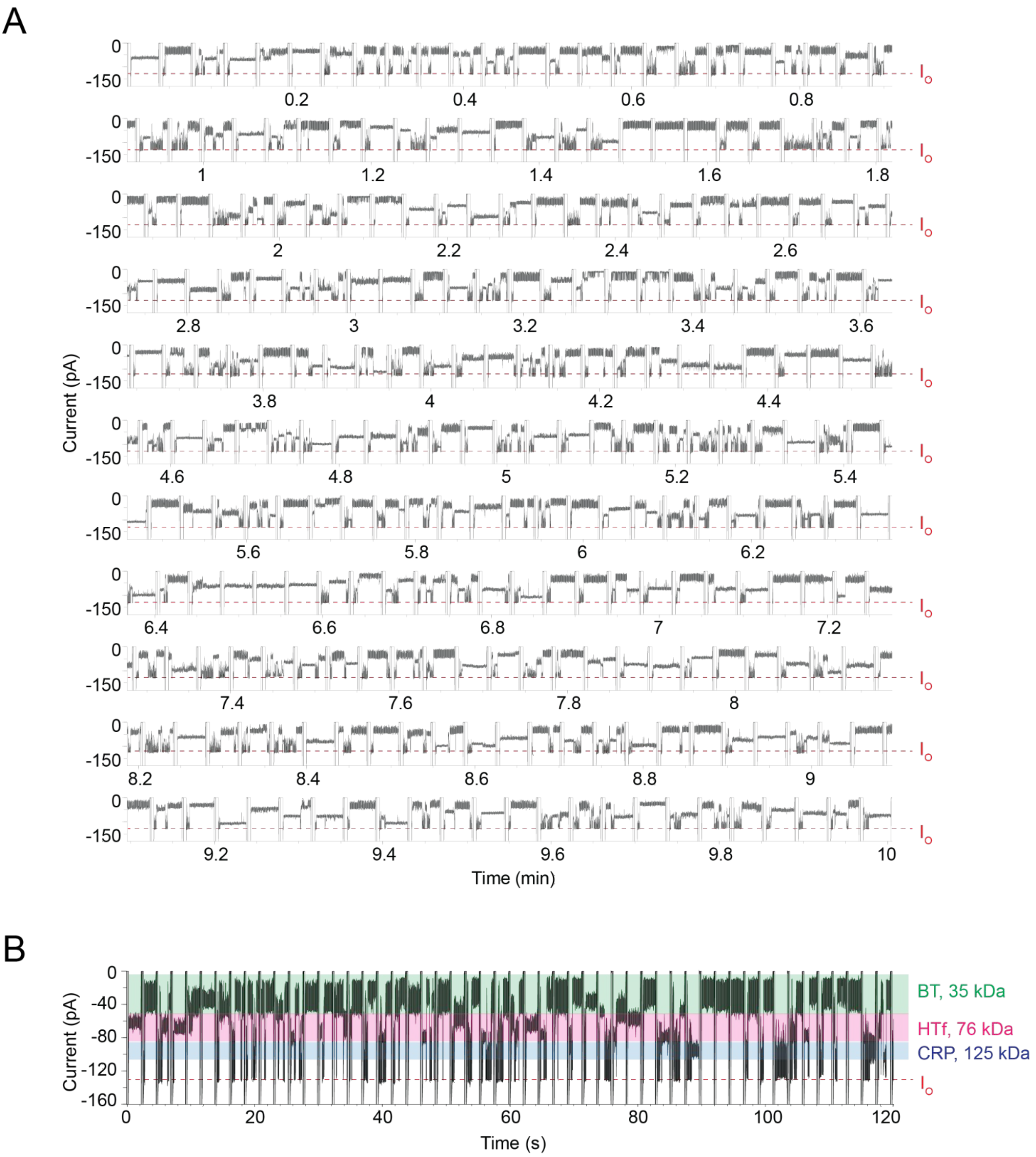

Supporting Figure 34: YaxA$_{\Delta40}$B$_{WT}$$^{1.9*}$ detecting mixed protein solution. A) 10 min electrophysiology trace and **B)** 2 min zoom-in of YaxA$_{\Delta40}$B$_{WT}$$^{1.9*}$ detecting mixed protein solution with high concentrations of BT (green), HTf (pink), CRP (blue) and BSA. I_O is indicated by red dotted line. 25% (v/v) mixed protein solution was added to *cis*, final concentrations in *cis*: 150 µM BSA, 8.8 µM HTf, 25 nM BT, 25 nM CRP. Measurement was performed at –75 mV in sweeps protocol, in 150 mM NaCl, 15 mM TrisHCl pH 7.5, and PDB$_{11}$PEO$_8$:DPhPC (1:1)-hybrid bilayer. Data were recorded at 50 kHz sampling rate, and 10 kHz Bessel filter. Trace was additionally filtered with 2 kHz low-pass Gaussian filter for visualization.

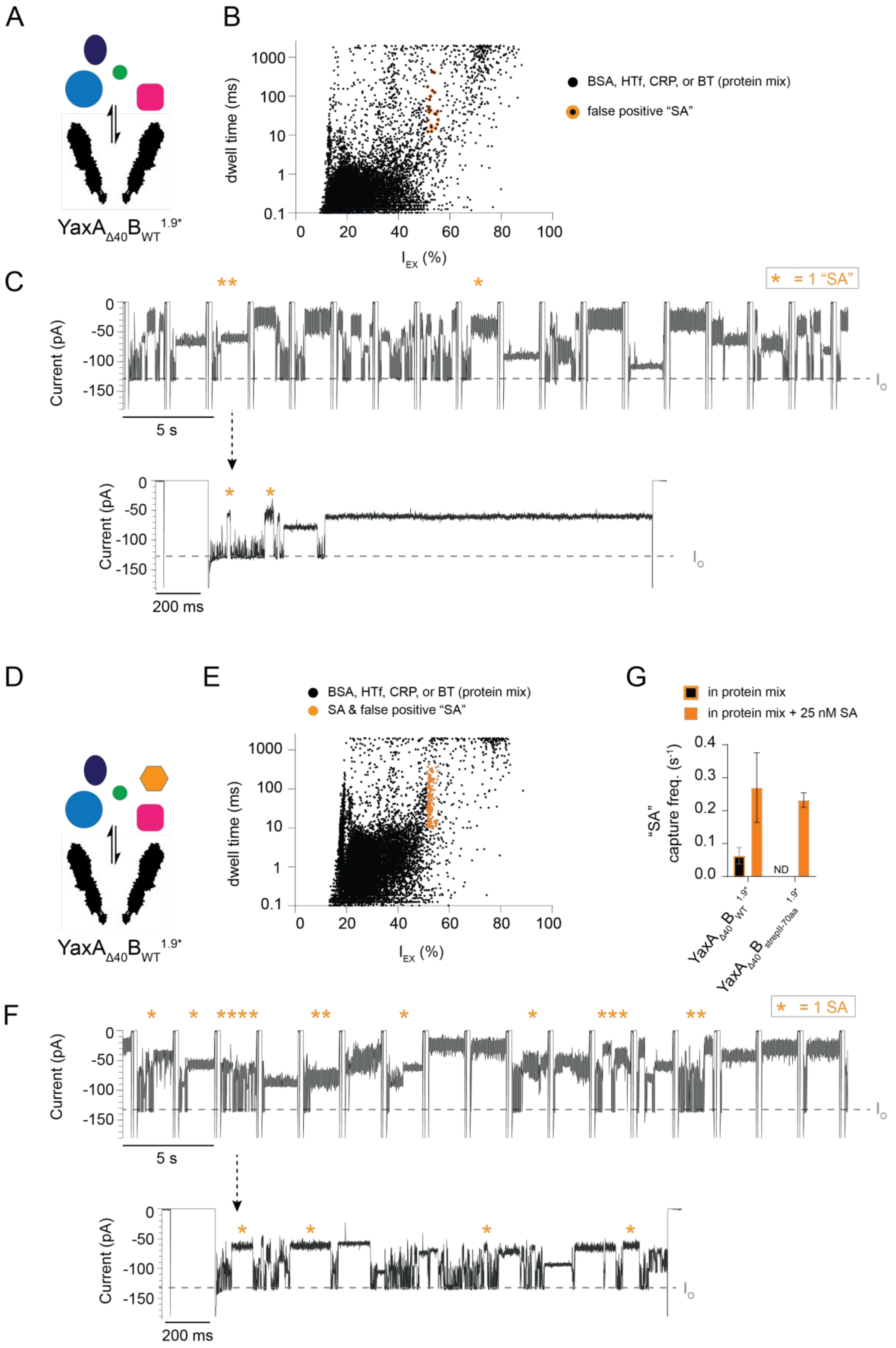

Supporting Figure 35: YaxA$_{\Delta 40}$B$_{WT}$$^{1.9*}$ detecting 25% mixed protein solution with 0 and 25 nM SA. A) Schematic picture of YaxA$_{\Delta 40}$B$_{WT}$$^{1.9*}$ detecting the mixed protein solution with four different proteins: BSA (dark blue), HTf (pink), CRP (light blue), BT (green). **B)** Scatterplot events of 10 min trace of single pore, false positive "SA"-events annotated in orange. **C)** Electrophysiology trace indicating false positive "SA"-events (orange star), with zoom-in of individual sweeps. I_O is indicated by grey dotted line. **D)** Schematic picture of YaxA$_{\Delta 40}$B$_{WT}$$^{1.9*}$ detecting the mixed protein solution with five different proteins: BSA, HTf, CRP, BT and SA (orange). **E)** Scatterplot events of 10 min trace of single pore, "SA"-events annotated in orange.

F) Electrophysiology trace indicating "SA"-events (orange star), with zoom-in of individual sweeps. **G)** Capture frequency of "SA" in mixed protein solution comparing $YaxA_{\Delta 40}B_{WT}^{1.9*}$ and $YaxA_{\Delta 40}B_{strepII\text{-}70aa}^{1.9*}$. In absence of SA, $YaxA_{\Delta 40}B_{WT}^{1.9*}$ depicted "SA"-events at 0.06 ± 0.03 s^{-1} (N=4 pores), compared to 0.27 ± 0.11 s^{-1} (N=6 pores) in presence of 25 nM SA. This suggests that, in 25% mixed protein solution with 25 nM SA, ~20% of "SA"-events are false positive in $YaxA_{\Delta 40}B_{WT}^{1.9*}$.25% (v/v) mixed protein solution was added to *cis*, final concentrations in *cis*: 150 µM BSA, 8.8 µM HTf, 25 nM BT, 25 nM CRP, and 0 or 25 nM SA, respectively. Only 1.9* pores were used. Measurement was performed at –75 mV in sweeps protocol, in 150 mM NaCl, 15 mM TrisHCl pH 7.5, and $PDB_{11}PEO_8$:DPhPC (1:1)-hybrid bilayer. Data were recorded at 50 kHz sampling rate, and 10 kHz Bessel filter. Trace was additionally filtered with 2 kHz low-pass Gaussian filter for visualization, but not for analysis. Events were labelled as "SA" if I_{EX}±2σ and log^{10}(dwell time)±2log^{10}(σ), of 1.9*-pores, see **Supporting Table 1**, *i.e.*, I_{EX} = 51.328–55.751 & log^{10}(dwell time) = 0.967–2.648.

A

B

C

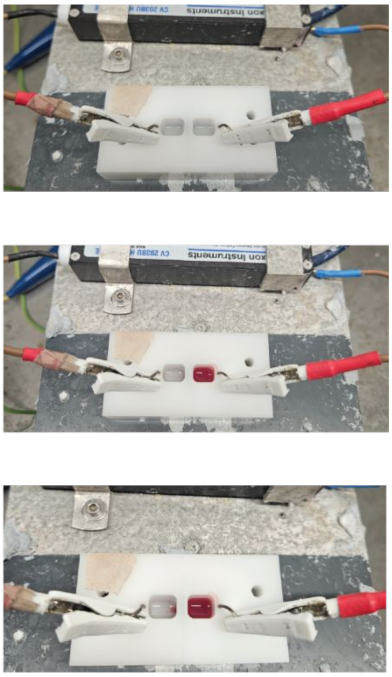

Supporting Figure 36: 25% (v/v) blood experiments. A) Chamber with buffer (150 mM NaCl, 15 mM TrisHCl pH 7.5) in *cis* and *trans*. **B)** The *cis*-compartment contains 25% whole blood (300 µL buffer + 100 µL whole blood) with intact bilayer. **C)** When the bilayer broke, blood can transfuse to *trans*-compartment *via* aperture.

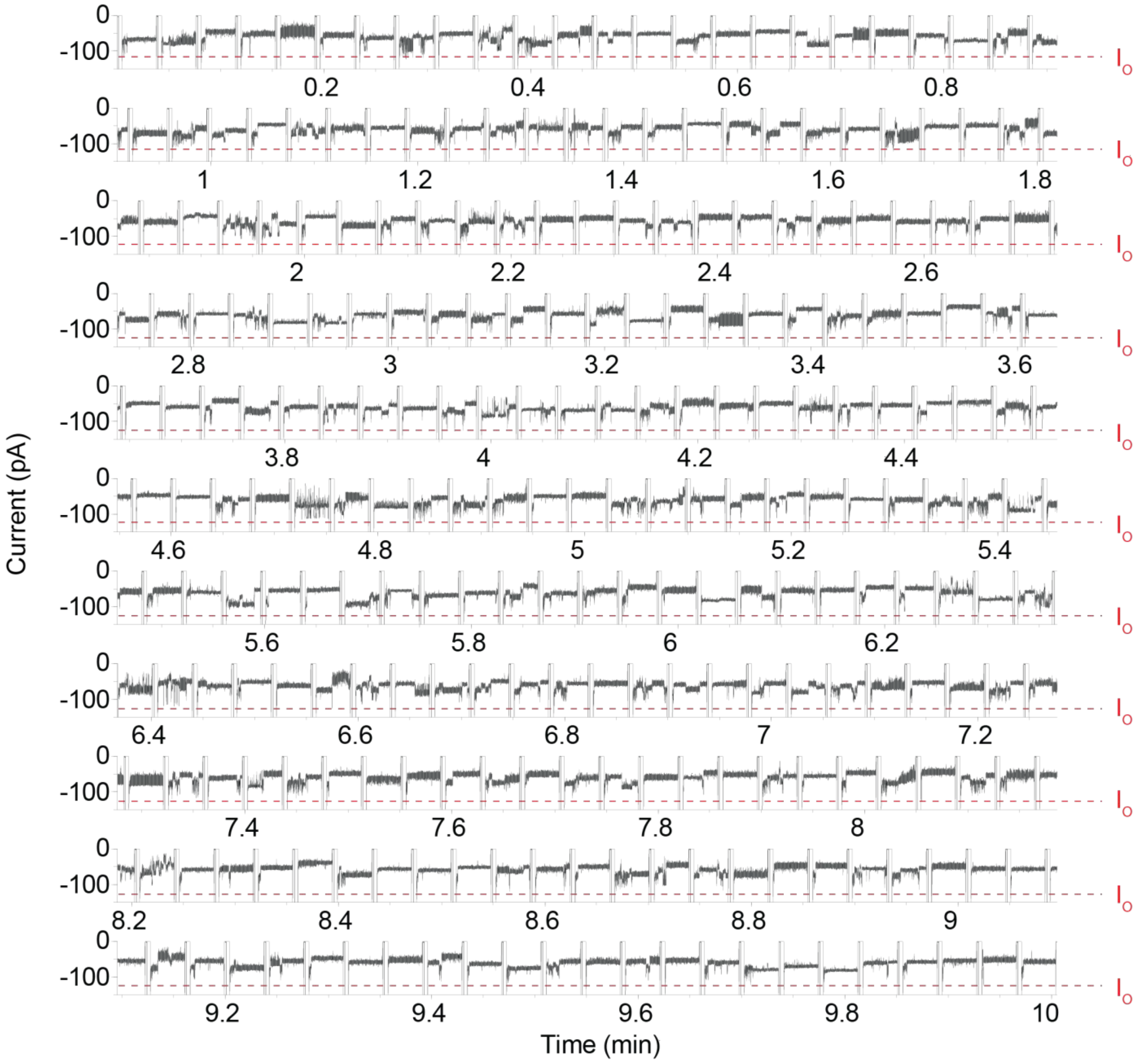

Supporting Figure 37: Blood proteins detected by YaxA$_{\Delta 40}$B$_{WT}$$^{1.9*}$. Electrophysiology trace of YaxA$_{\Delta 40}$B$_{WT}$$^{1.9*}$ detecting whole, defibrinated sheep blood at 25% (v/v) final concentration in *cis*. I_O is indicated by red dotted line. Blood was added after pore insertion. Measurement was recorded for 10 min, at –75 mV using a sweeps protocol in PDB$_{11}$PEO$_8$:DPhPC (1:1)-hybrid bilayer. Data were recorded at 50 kHz sampling rate, and 10 kHz Bessel filter. Trace was additionally filtered with 2 kHz low-pass Gaussian filter for visualization.

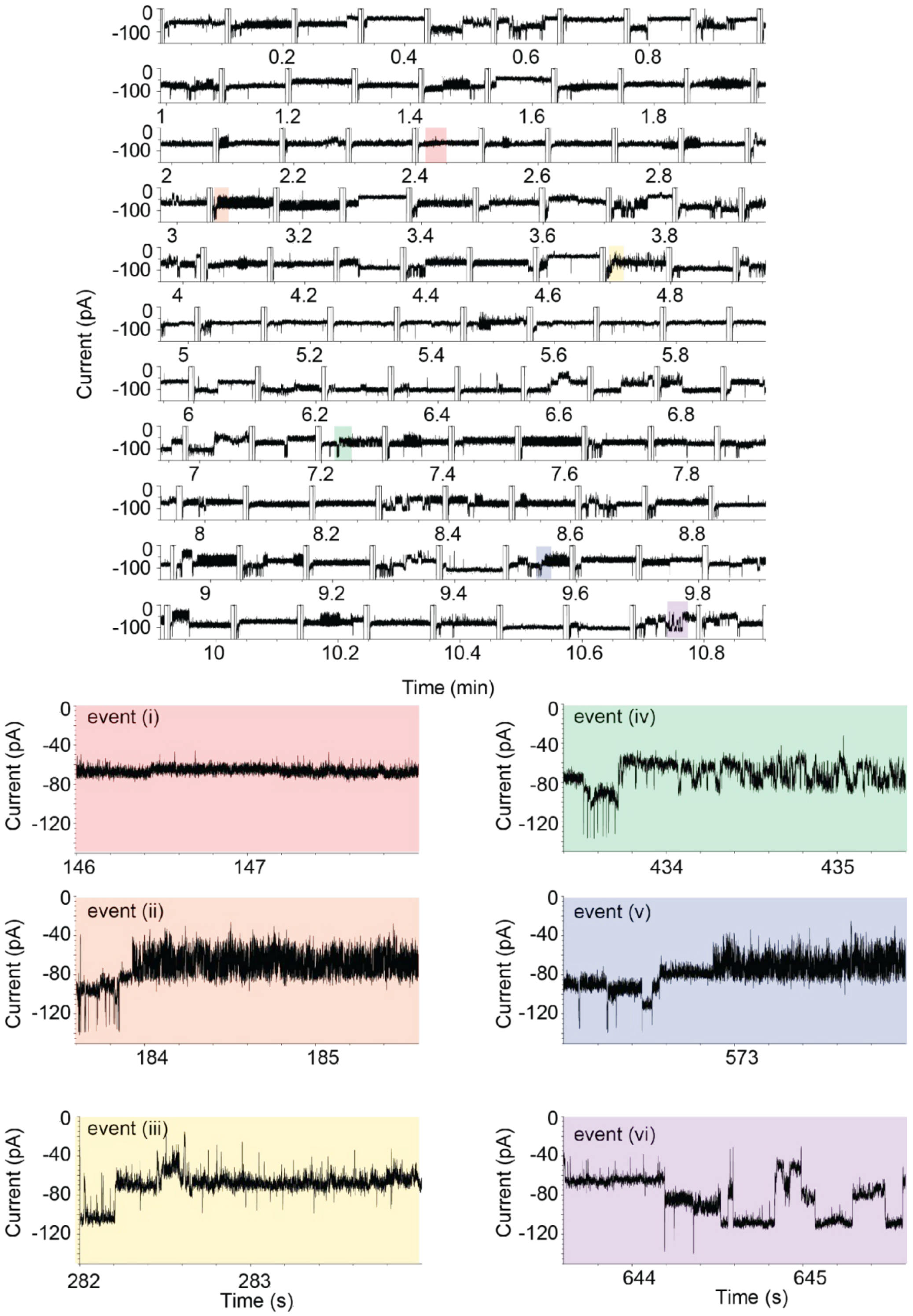

Supporting Figure 38: Blood proteins detected by YaxA$_{\Delta 40}$B$_{\text{strepII-70aa}}$$^{1.9*}$. Electrophysiology trace (10 min) of YaxA$_{\Delta 40}$B$_{\text{strepII-70aa}}$$^{1.9*}$ detecting whole, defibrinated sheep blood at 25% (v/v) final concentration in *cis* (measurement 1). Examples of long dwelling blockades (i) and multilevel blockades (ii-vi) are indicated, but SA-blockades were not observed. Measurements was recorded for >10 min, at –75 mV in sweeps protocol, in 150 mM NaCl, 15 mM TrisHCl pH 7.5, and PDB$_{11}$PEO$_8$:DPhPC (1:1)-hybrid bilayer. Data were recorded at 50 kHz sampling rate, and 10 kHz Bessel filter. Trace was additionally filtered with 2 kHz low-pass Gaussian filter for visualization.

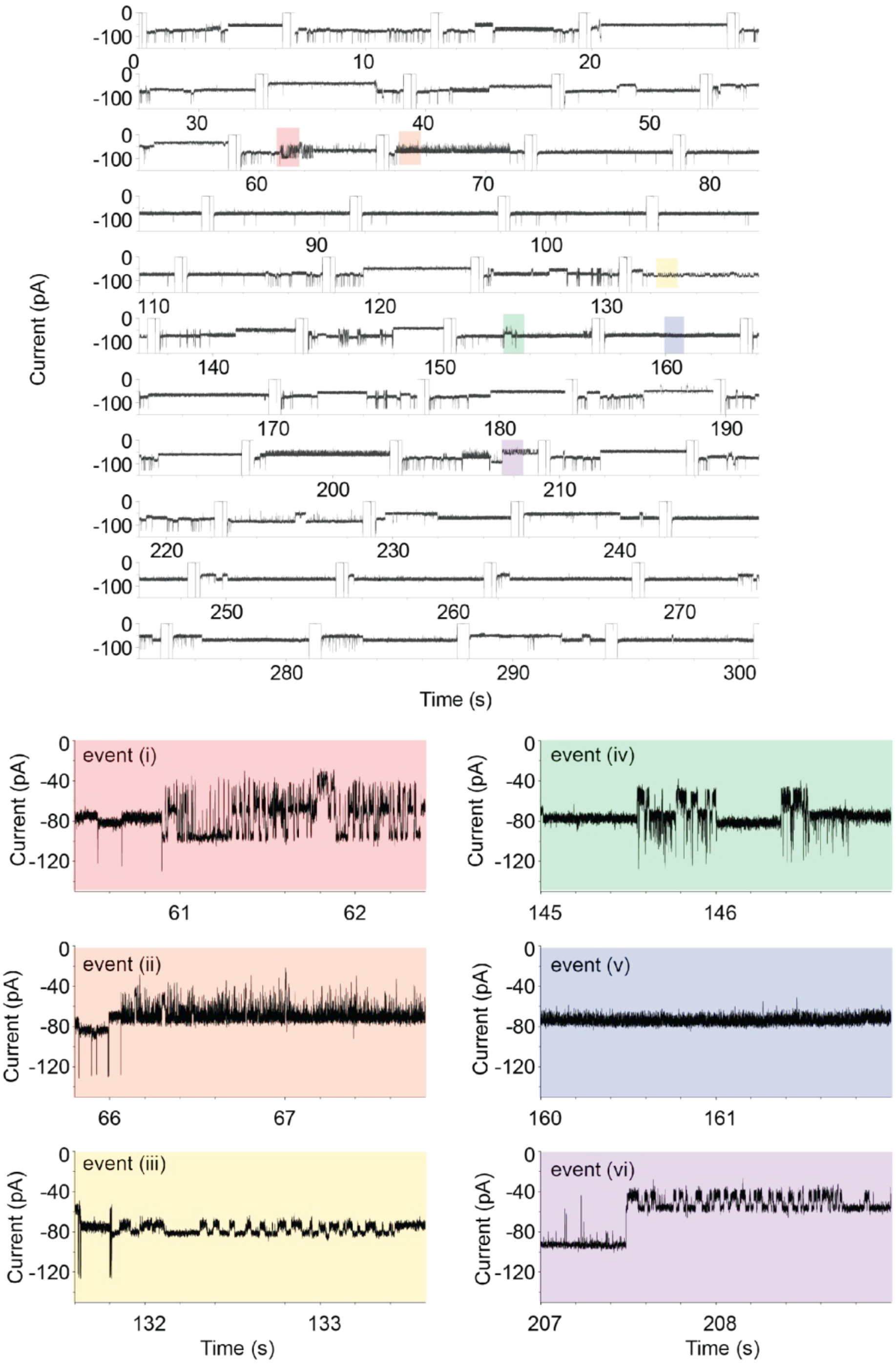

Supporting Figure 39: Blood proteins detected by YaxA$_{\Delta 40}$B$_{strepII\text{-}70aa}$$^{1.9*}$. Electrophysiology trace (5 min) of YaxA$_{\Delta 40}$B$_{strepII\text{-}70aa}$$^{1.9*}$ detecting whole, defibrinated sheep blood at 25% (v/v) final concentration in *cis* (measurement 2). Examples of long dwelling blockades (v) and multilevel blockades (i-iv,vi) are indicated, but SA-blockades were not observed. Measurements was recorded for 5 min, at –75 mV in sweeps protocol, in 150 mM NaCl, 15 mM TrisHCl pH 7.5, and PDB$_{11}$PEO$_8$:DPhPC (1:1)-hybrid bilayer. Data were recorded at 50 kHz sampling rate, and 10 kHz Bessel filter. Trace was additionally filtered with 2 kHz low-pass Gaussian filter for visualization.

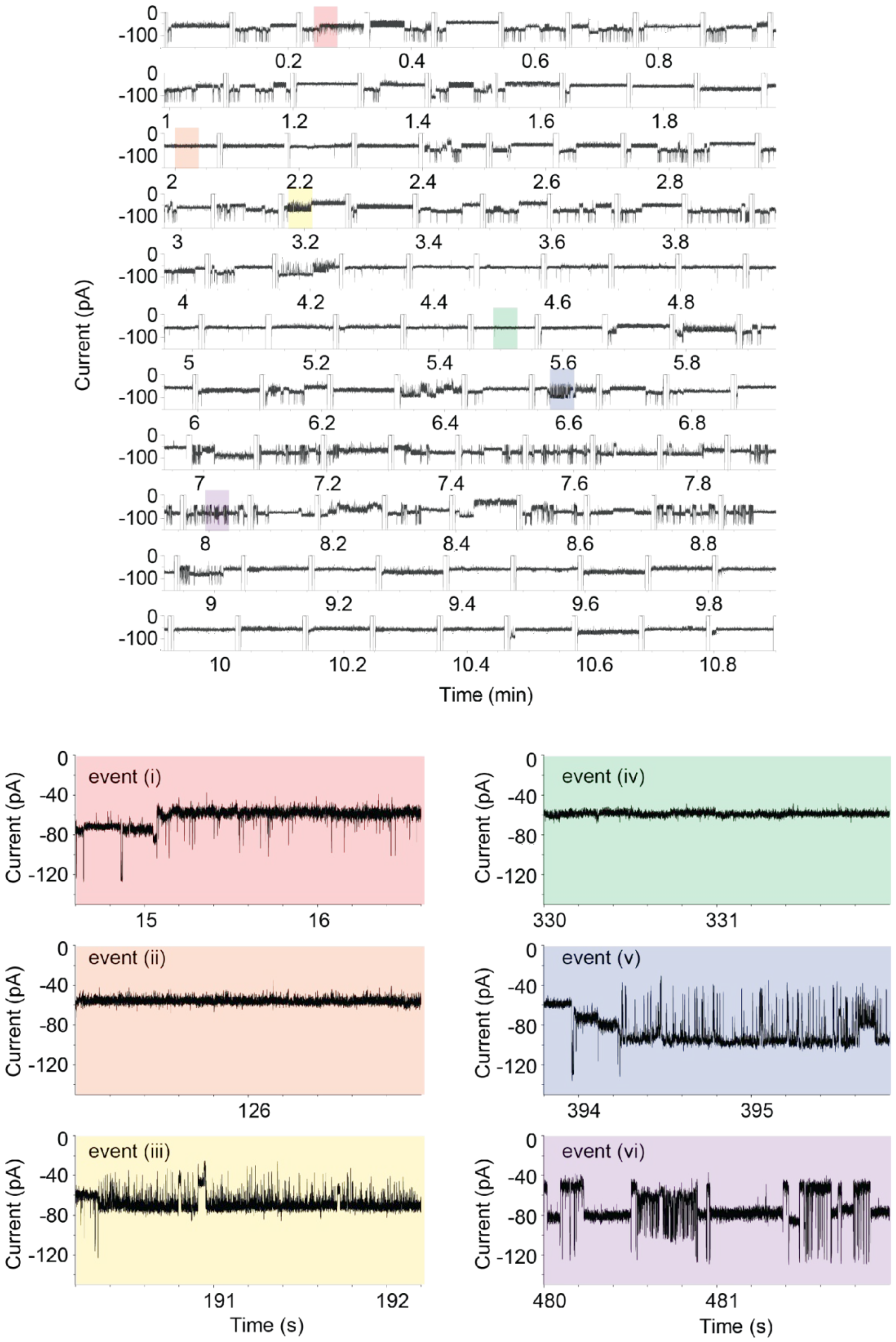

Supporting Figure 40: Blood proteins detected by YaxA$_{\Delta40}$B$_{strepII\text{-}70aa}$$^{1.9*}$. Electrophysiology trace (10 min) of YaxA$_{\Delta40}$B$_{strepII\text{-}70aa}$$^{1.9*}$ detecting whole, defibrinated sheep blood at 25% (v/v) final concentration in *cis* (measurement 3). Examples of long dwelling blockades (ii, iv) and multilevel blockades (i, iii, v, vi) are indicated, but SA-blockades were not observed. Measurements was recorded for >10 min, at –75 mV in sweeps protocol, in 150 mM NaCl, 15 mM TrisHCl pH 7.5, and PDB$_{11}$PEO$_8$:DPhPC (1:1)-hybrid bilayer. Data were recorded at 50 kHz sampling rate, and 10 kHz Bessel filter. Trace was additionally filtered with 2 kHz low-pass Gaussian filter for visualization.

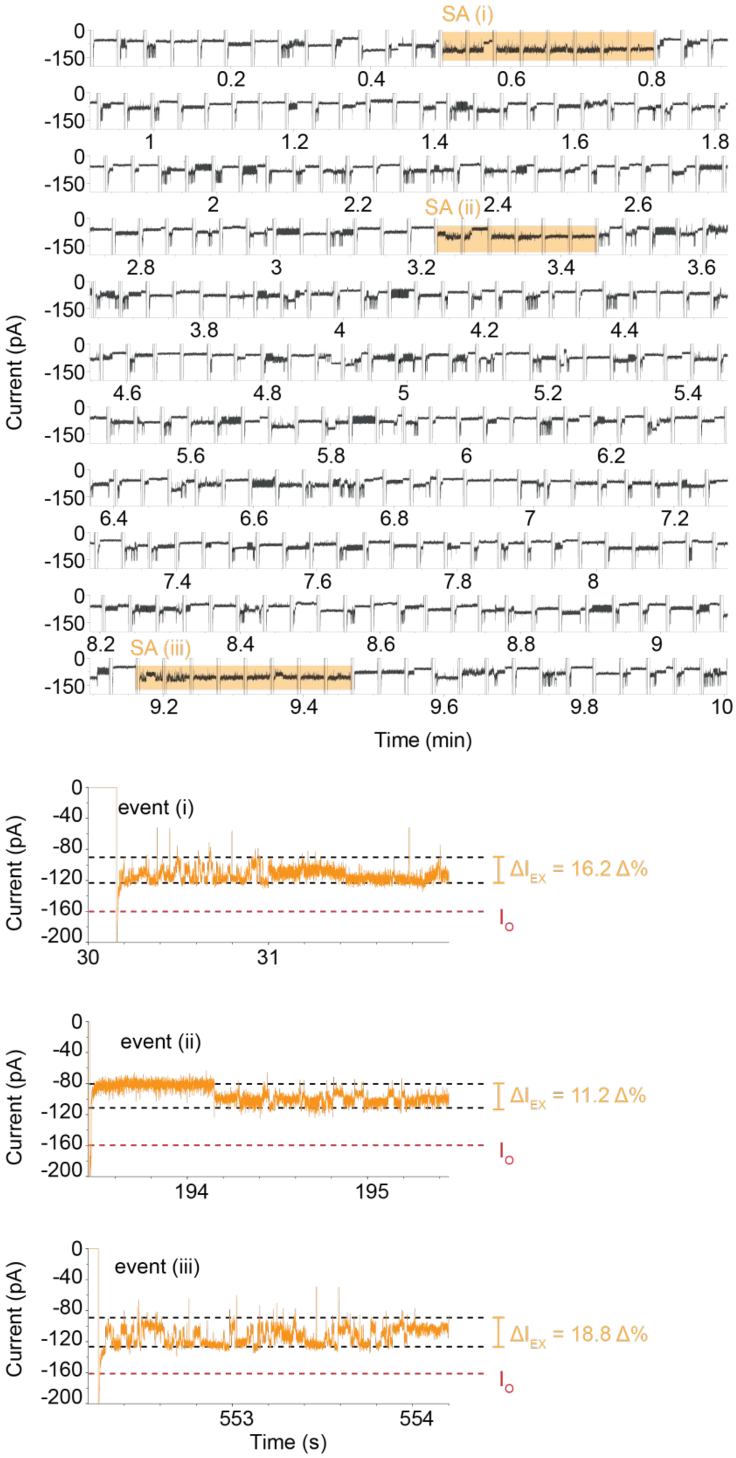

Supporting Figure 41: Nanomolar SA detection in blood by YaxA$_{\Delta 40}$B$_{strepII\text{-}70aa}$$^{2.3*}$. Electrophysiology trace of YaxA$_{\Delta 40}$B$_{strepII\text{-}70aa}$$^{2.3*}$ detecting whole, defibrinated sheep blood premixed with SA (extended data from Figure 7 main text; measurement 1). Defibrinated sheep blood was premixed with 50 nM SA. After YaxA$_{\Delta 40}$B$_{strepII\text{-}70aa}$$^{2.3*}$ nanopore insertion, 40 µL of buffer in *cis* was replaced 40 µL of spiked blood (*i.e.*, 10% dilution, 5 nM SA final concentration in *cis*) with biotin (10 nM final concentration) added to *trans* to increase the off-rate of SA-strepII binding. In 10 minutes recording, three characteristic SA-blockades (orange) can be observed among blood proteins. Measurements were conducted in 150 mM NaCl, 15 mM TrisHCl pH 7.5, and PDB$_{11}$PEO$_8$:DPhPC (1:1)-hybrid bilayer. Data were recorded at –75 mV in sweeps protocol, at 50 kHz sampling rate, and 10 kHz Bessel filter. Traces were additionally filtered with 2 kHz low-pass Gaussian filter for visualization.

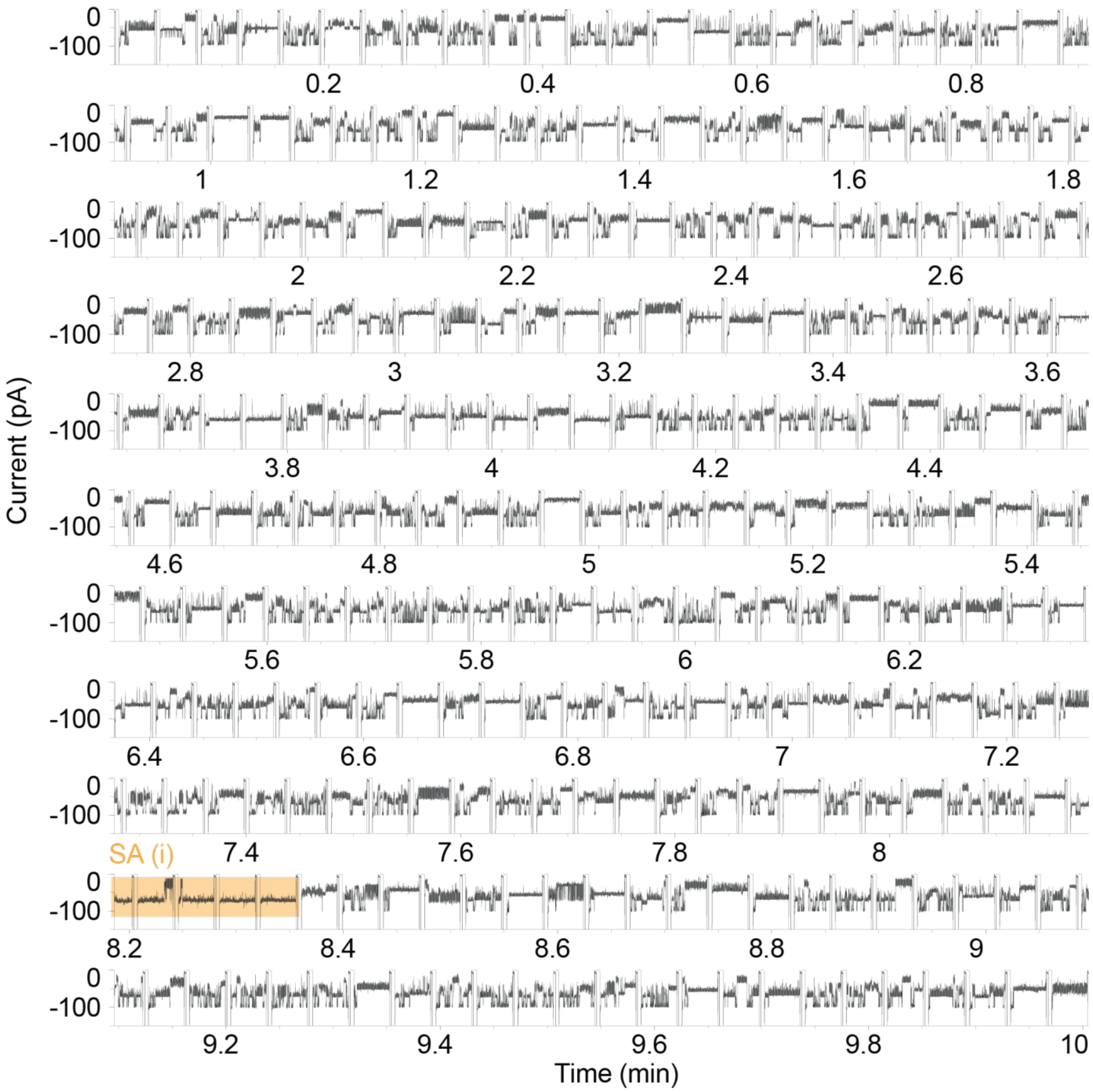

Supporting Figure 42: YaxA$_{\Delta40}$B$_{strepII\text{-}70aa}$$^{1.6*}$ detecting 25% whole blood and SA. Electrophysiology trace of YaxA$_{\Delta40}$B$_{strepII\text{-}70aa}$$^{1.6*}$ detecting whole, defibrinated sheep blood mixed with SA (measurement 2). After YaxA$_{\Delta40}$B$_{strepII\text{-}70aa}$$^{1.6*}$ nanopore insertion, 100 µL of buffer in *cis* was replaced 100 µL of whole blood (*i.e.*, 25% dilution) and then SA was added on top (~4 nM SA final concentration in *cis*) with biotin (125 nM final concentration) added to *trans* to increase the off-rate of SA-strepII binding. In 10 minutes recording, one characteristic SA-blockades (orange) can be observed among blood proteins. Measurements were conducted in 150 mM NaCl, 15 mM TrisHCl pH 7.5, and PDB$_{11}$PEO$_{8}$:DPhPC (1:1)-hybrid bilayer. Data were recorded at –75 mV in sweeps protocol, at 50 kHz sampling rate, and 10 kHz Bessel filter. Traces were additionally filtered with 2 kHz low-pass Gaussian filter for visualization.

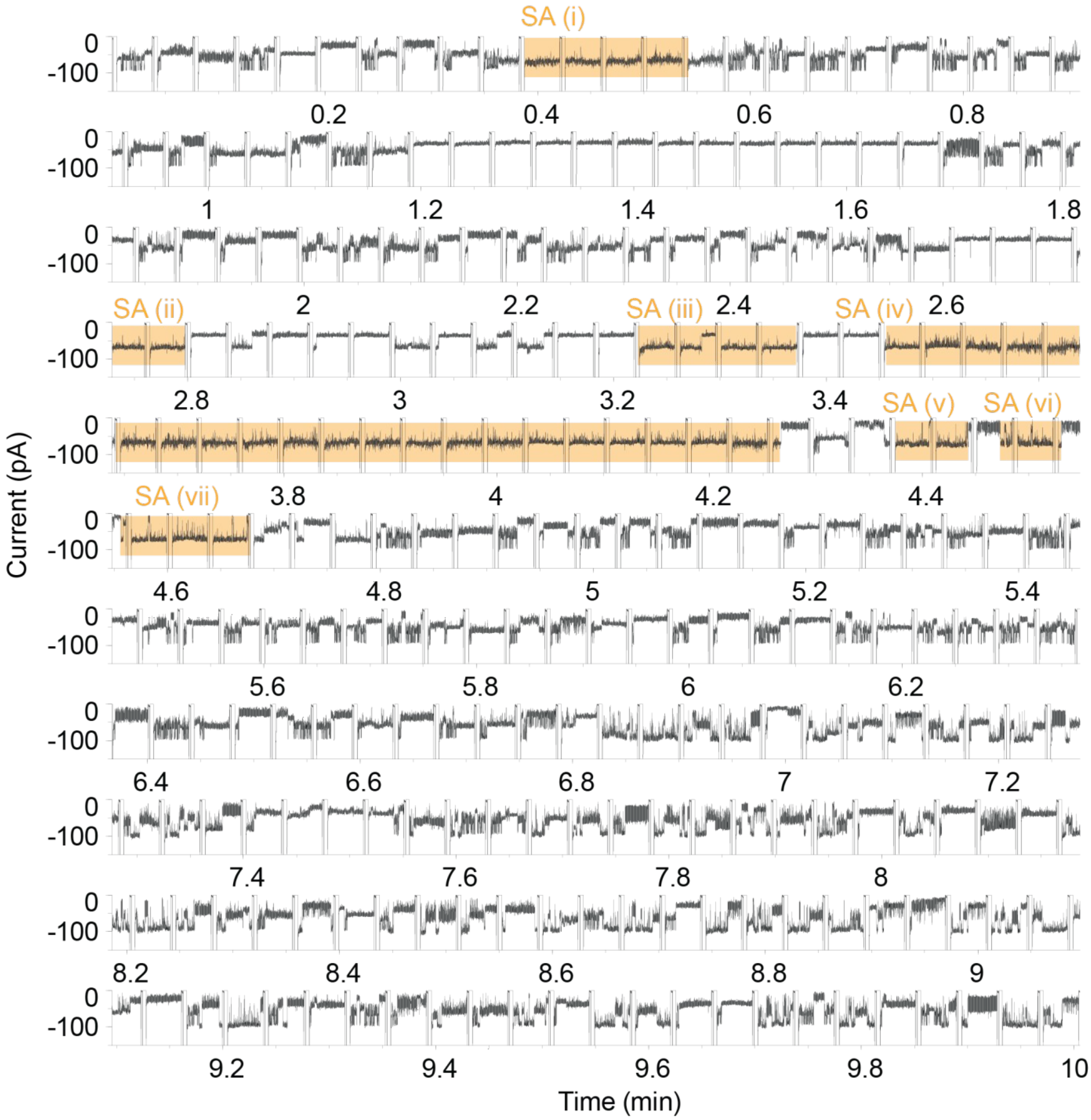

Supporting Figure 43: YaxA$_{\Delta40}$B$_{strepII\text{-}70aa}$$^{1.6*}$ detecting 25% whole blood and SA. Electrophysiology trace of YaxA$_{\Delta40}$B$_{strepII\text{-}70aa}$$^{1.6*}$ detecting whole, defibrinated sheep blood mixed with SA (measurement 3). After YaxA$_{\Delta40}$B$_{strepII\text{-}70aa}$$^{1.6*}$ nanopore insertion, 100 µL of buffer in *cis* was replaced 100 µL of whole blood (*i.e.*, 25% dilution) and then SA was added on top (~4 nM SA final concentration in *cis*) with biotin (125 nM final concentration) added to *trans* to increase the off-rate of SA-strepII binding. In 10 minutes recording, seven characteristic SA-blockades (orange) can be observed among blood proteins. Measurements were conducted in 150 mM NaCl, 15 mM TrisHCl pH 7.5, and PDB$_{11}$PEO$_8$:DPhPC (1:1)-hybrid bilayer. Data were recorded at -75 mV in sweeps protocol, at 50 kHz sampling rate, and 10 kHz Bessel filter. Traces were additionally filtered with 2 kHz low-pass Gaussian filter for visualization.

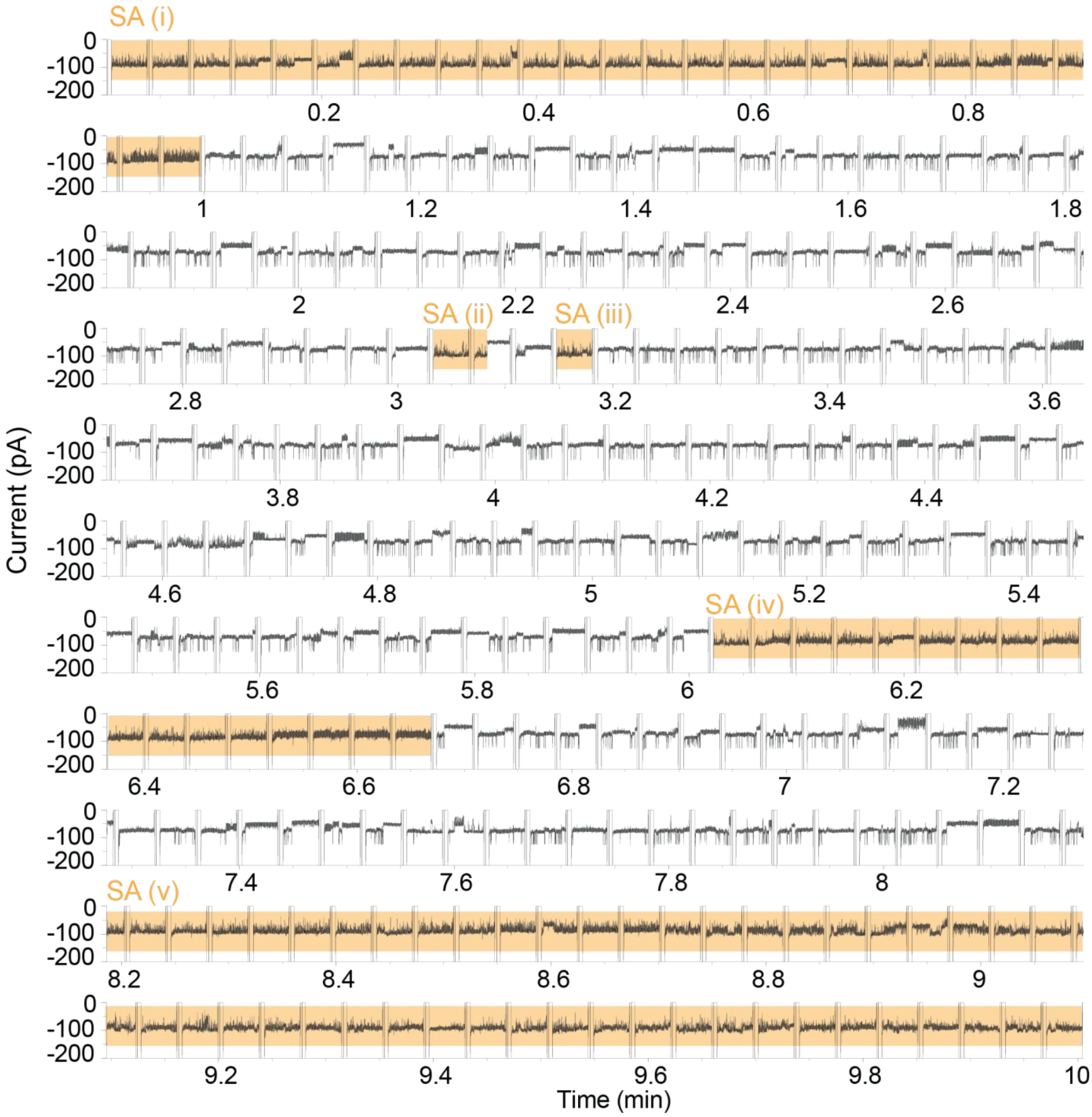

Supporting Figure 44: YaxA$_{\Delta 40}$B$_{strepII\text{-}70aa}$$^{1.9*}$ detecting 10% SA-spiked whole blood. Electrophysiology trace of YaxA$_{\Delta 40}$B$_{strepII\text{-}70aa}$$^{1.9*}$ detecting whole, defibrinated sheep blood premixed with SA (measurement 4). Whole blood was premixed with SA at 50 nM concentration. After YaxA$_{\Delta 40}$B$_{strepII\text{-}70aa}$$^{1.9*}$ nanopore insertion, 40 µL of buffer in *cis* was replaced 40 µL of whole blood spiked with SA (*i.e.*, 10% dilution, 5 nM SA final concentration in *cis*) with biotin (10 nM final concentration) added to *trans* to increase the off-rate of SA-strepII binding. In 10 minutes recording, five characteristic SA-blockades (orange) can be observed among blood proteins. Measurements were conducted in 150 mM NaCl, 15 mM TrisHCl pH 7.5, and PDB$_{11}$PEO$_8$:DPhPC (1:1)-hybrid bilayer. Data were recorded at –75 mV in sweeps protocol, at 50 kHz sampling rate, and 10 kHz Bessel filter. Traces were additionally filtered with 2 kHz low-pass Gaussian filter for visualization.

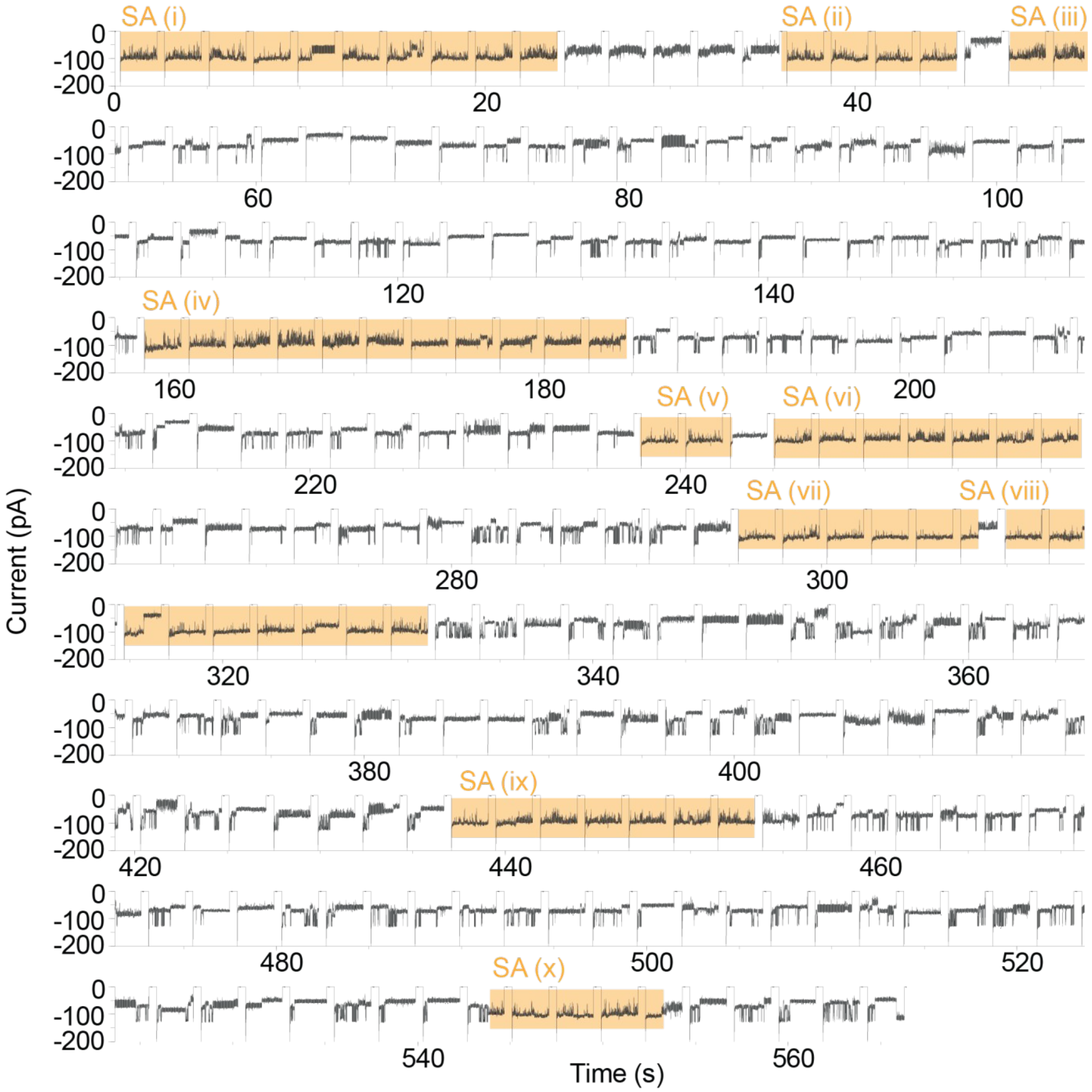

Supporting Figure 45: YaxA$_{\Delta 40}$B$_{strepII\text{-}70aa}$$^{1.9*}$ detecting 10% SA-spiked blood supernatant. Electrophysiology trace of YaxA$_{\Delta 40}$B$_{strepII\text{-}70aa}$$^{1.9*}$ detecting supernatant of defibrinated sheep blood premixed with SA (measurement 5). Whole blood was premixed with SA at 50 nM concentration, and the blood cells were pelleted with centrifugation (1 min, 10.000*g*). Supernatant containing SA was used next, and the pelleted cells were discarded. After YaxA$_{\Delta 40}$B$_{strepII\text{-}70aa}$$^{1.9*}$ nanopore insertion, 40 µL of buffer in *cis* was replaced 40 µL of spun-down blood spiked with SA (*i.e.*, 10% dilution, 5 nM SA final concentration in *cis*) with biotin (10 nM final concentration) added to *trans* to increase the off-rate of SA-strepII binding. In ~10 minutes recording, ten characteristic SA-blockades (orange) can be observed among blood proteins. Trace is cropped due to second pore insertion. Measurements were conducted in 150 mM NaCl, 15 mM TrisHCl pH 7.5, and PDB$_{11}$PEO$_8$:DPhPC (1:1)-hybrid bilayer. Data were recorded at –75 mV in sweeps protocol, at 50 kHz sampling rate, and 10 kHz Bessel filter. Traces were additionally filtered with 2 kHz low-pass Gaussian filter for visualization.

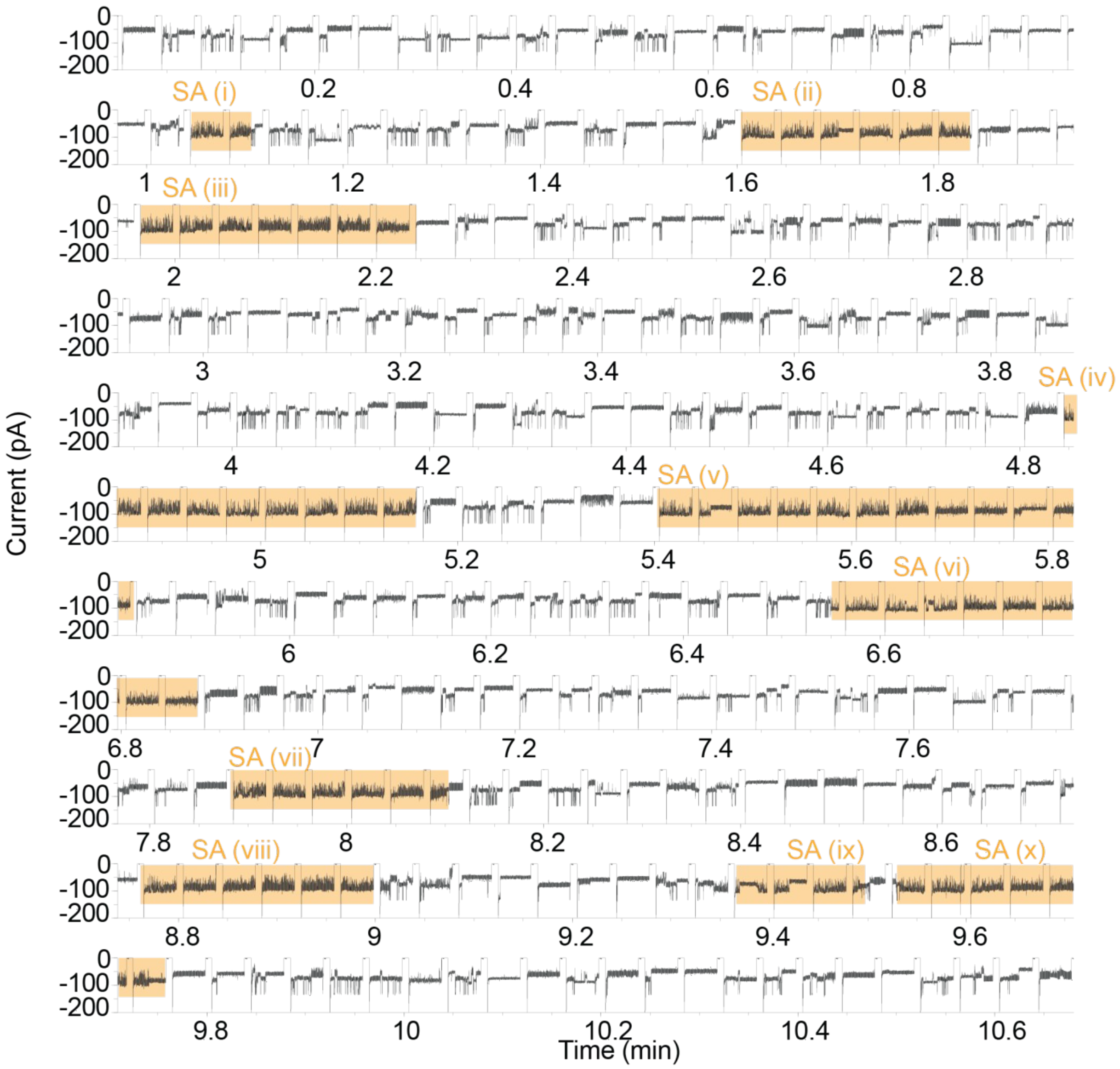

Supporting Figure 46: YaxA$_{\Delta40}$B$_{strepII\text{-}70aa}$$^{1.9*}$ detecting 10% SA-spiked blood supernatant. Electrophysiology trace of YaxA$_{\Delta40}$B$_{strepII\text{-}70aa}$$^{1.9*}$ detecting supernatant of defibrinated sheep blood premixed with SA (measurement 6). Whole blood was premixed with SA at 50 nM concentration, and the blood cells were pelleted with centrifugation (1 min, 10.000*g*). Supernatant containing SA was used next, and the pelleted cells were discarded. After YaxA$_{\Delta40}$B$_{strepII\text{-}70aa}$$^{1.9*}$ nanopore insertion, 40 µL of buffer in *cis* was replaced 40 µL of spun-down blood spiked with SA (*i.e.*, 10% dilution, 5 nM SA final concentration in *cis*) with biotin (10 nM final concentration) added to *trans* to increase the off-rate of SA-strepII binding. In 10 minutes recording, ten characteristic SA-blockades (orange) can be observed among blood proteins. Measurements were conducted in 150 mM NaCl, 15 mM TrisHCl pH 7.5, and PDB$_{11}$PEO$_8$:DPhPC (1:1)-hybrid bilayer. Data were recorded at –75 mV in sweeps protocol, at 50 kHz sampling rate, and 10 kHz Bessel filter. Traces were additionally filtered with 2 kHz low-pass Gaussian filter for visualization.

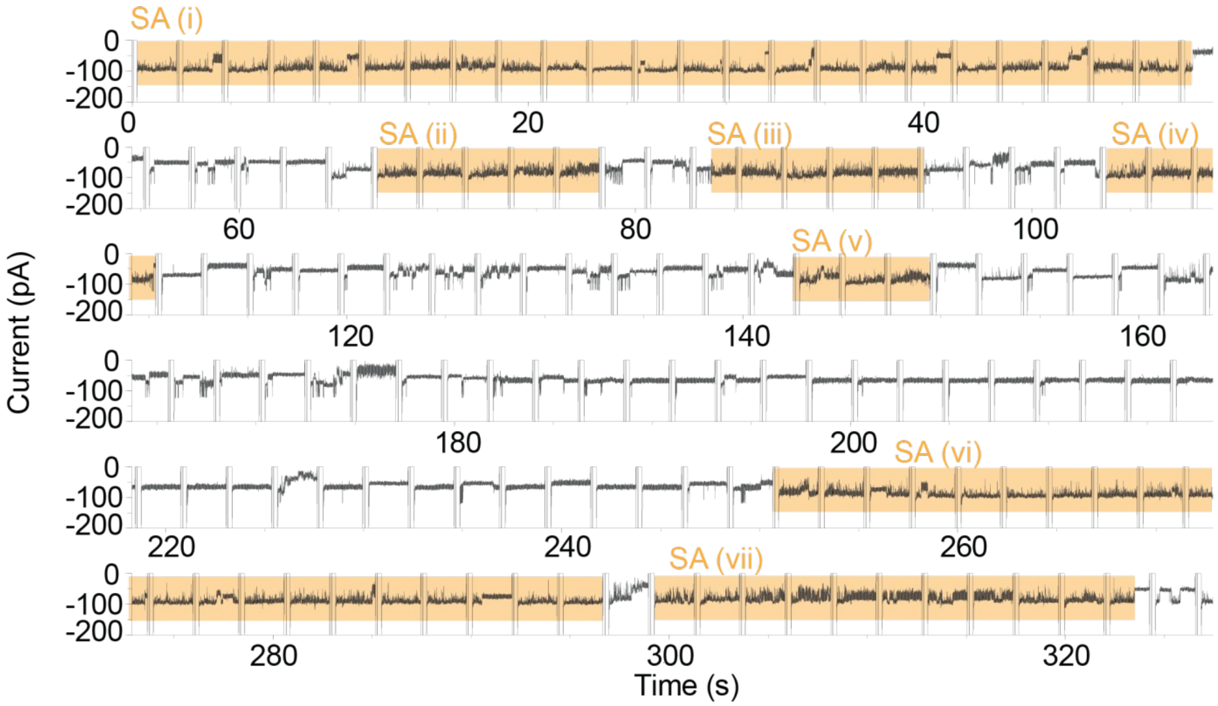

Supporting Figure 47: YaxA$_{\Delta 40}$B$_{\text{strepII-70aa}}$$^{1.9*}$ detecting 25% SA-spiked blood supernatant. Electrophysiology trace of YaxA$_{\Delta 40}$B$_{\text{strepII-70aa}}$$^{1.9*}$ detecting supernatant of defibrinated sheep blood premixed with SA (measurement 7). Whole blood was premixed with SA at 50 nM concentration, and the blood cells were pelleted with centrifugation (1 min, 10.000*g*). Supernatant containing SA was used next, and the pelleted cells were discarded. After YaxA$_{\Delta 40}$B$_{\text{strepII-70aa}}$$^{1.9*}$ nanopore insertion, 100 µL of buffer in *cis* was replaced 100 µL of spun-down blood spiked with SA (*i.e.*, 25% dilution, ~13 nM SA final concentration in *cis*) with biotin (10 nM final concentration) added to *trans* to increase the off-rate of SA-strepII binding. In >5 minutes recording, seven characteristic SA-blockades (orange) can be observed among blood proteins. Measurements were conducted in 150 mM NaCl, 15 mM TrisHCl pH 7.5, and PDB$_{11}$PEO$_8$:DPhPC (1:1)-hybrid bilayer. Data were recorded at -75 mV in sweeps protocol, at 50 kHz sampling rate, and 10 kHz Bessel filter. Traces were additionally filtered with 2 kHz low-pass Gaussian filter for visualization.

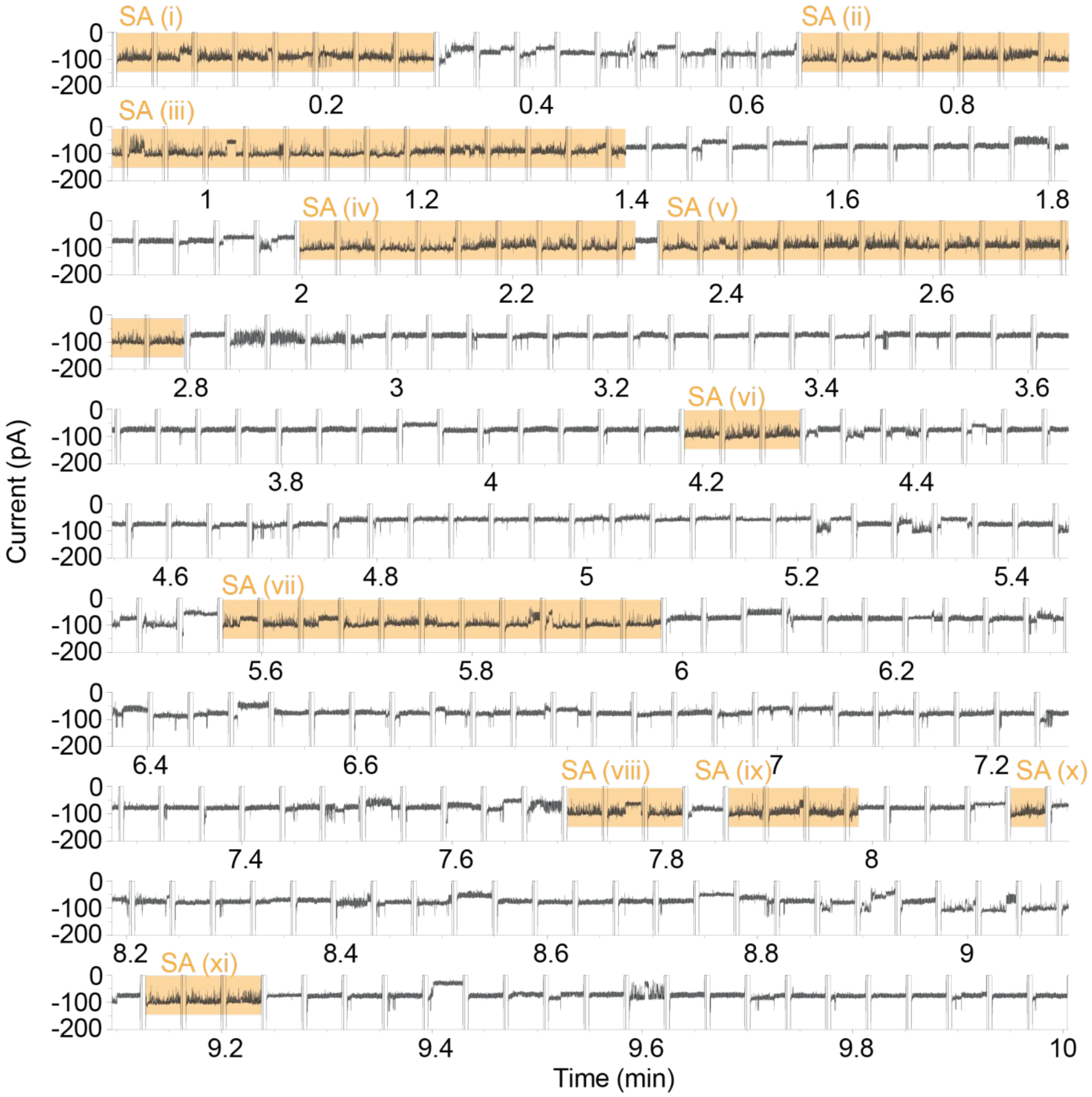

Supporting Figure 48: YaxA$_{\Delta40}$B$_{strepII\text{-}70aa}$$^{1.9*}$ detecting 25% blood supernatant supplemented with SA. Electrophysiology trace of YaxA$_{\Delta40}$B$_{strepII\text{-}70aa}$$^{1.9*}$ detecting supernatant of defibrinated sheep blood supplemented with SA (measurement 8). Whole blood was premixed with SA at 4 nM concentration, and the blood cells were pelleted with centrifugation (1 min, 10.000*g*). Supernatant containing SA was used next, and the pelleted cells were discarded. After YaxA$_{\Delta40}$B$_{strepII\text{-}70aa}$$^{1.9*}$ nanopore insertion, 100 µL of buffer in *cis* was replaced 100 µL of spun-down blood spiked with SA (*i.e.*, 10% dilution) and an additional SA was added (~20 nM final concentration in *cis*) with biotin (10 nM final concentration) added to *trans* to increase the off-rate of SA-strepII binding. In 10 minutes recording, 11 characteristic SA-blockades (orange) can be observed among blood proteins. Measurements were conducted in 150 mM NaCl, 15 mM TrisHCl pH 7.5, and PDB$_{11}$PEO$_{8}$:DPhPC (1:1)-hybrid bilayer. Data were recorded at –75 mV in sweeps protocol, at 50 kHz sampling rate, and 10 kHz Bessel filter. Traces were additionally filtered with 2 kHz low-pass Gaussian filter for visualization.

Supplementary Note: Theoretical Comparison of Capture Rates in functionalized YaxAB Nanopore, With and Without an Entropic Gate

We compare the capture rates of a particle in two nanopore systems:

1. A nanopore without an entropic gate.

2. The same nanopore with an entropic gate.

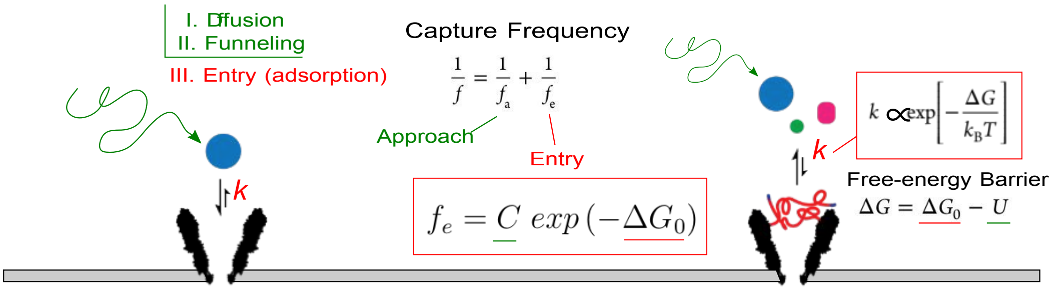

Supporting Figure 49: Schematic representation of the capture process in nanopores with and without an entropic gate. The capture process is divided into three stages: (i) Diffusion and approach (green, identical in both cases), (ii) Funneling (green, identical in both cases), and (iii) Entry (red, different in the two cases).

Theoretical models of polyelectrolyte capture by a nanopore can be derived from transport equations with appropriate boundary conditions[6]. Typically, the models include: i) the concentration of the analytes far from the pore, e.g. by fixing it at a constant value $C(r \to \infty) = C_0$; iii) adsorbing boundary conditions at the pore entrance, linked to the “docking” or “entrance” probability of a particle into the nanopore; iv) impermeability to the ions and water flow across the membrane; v) applied voltage. The diffusivity of the particles in the medium have to be imposed also. For example, Chinappi and Cecconi[7] derived analytical formulas for the total capture rate f_c, which can be generally expressed as

$$\frac{1}{f_c} = \frac{1}{f_a} + \frac{1}{f_{e,}} \qquad \text{(S1)}$$

where f_a corresponds to the approach and funneling phase and f_e represents the entry phase. The model was used to interpret the data from our previous work on the $YaxA_{\Delta 40}B$ nanopore. Here, we quickly summarize the general protocol adopted to interpret the capture rates.

Approach Frequency: Diffusion and Funneling

The flux $J(r)$ of proteins toward the pore is governed by

$$J(r) = -D\frac{\partial C(r)}{\partial r} + \mu F\, C(r) + u_f\, C(r), \qquad \text{(S2)}$$

where

- D is the diffusion coefficient,
- $C(r)$ is the protein concentration at distance r,
- μ is the protein mobility,
- F represents external forces (e.g., electrophoretic, dielectrophoretic), and
- u_f is the advection velocity (e.g., electroosmotic flow).

Under steady-state conditions,

$$\frac{d}{dr}\left[D\frac{dC(r)}{dr} - \mu F\, C(r) - u_f\, C(r)\right] = 0. \qquad \text{(S3)}$$

The approach frequency is given by

$$f_a = \frac{2\pi C_0 D}{\int_{r_e}^{\infty} dp\, \frac{e^{-\phi(p)}}{p^2}}, \qquad \text{(S4)}$$

where $\phi(r)$ represents the potential field affecting particle motion—particularly relevant as the particle approaches the pore entrance (the funneling stage). A model for $\phi(r)$, including electrophoresis, dielectrophoresis, and electro-osmosis, is discussed in the original paper[7]. The model incorporates the total current I, the charge and dipole of the particle, and the advection contribution (assuming the presence of electro-osmotic flow, EOF). Using this model, we previously showed that the limiting step in protein capture in the $YaxA_{\Delta 40}B$ nanopore is primarily related to the entrance barrier.[1] Indeed, the approach rates f_a predicted by this model, using the experimental concentrations (from 20 to 120 nM), are more than one order of magnitude larger than the experimentally measured global capture rates f_c.

Since the estimated difference in open pore current between the functionalized and nonfunctionalized nanopores ($YaxA_{\Delta 40}B_{WT}$ and $YaxA_{\Delta 40}B_{strepII\text{-}70aa}$, see **Supporting Figure 5**) remains within ±10%, we assume that the entropic gate does not significantly alter the approach frequency. This is consistent with the observation that the transport properties of the large YaxAB nanopore are primarily dictated by the transmembrane region, far from the localized functionalized linkers. Moreover, MD simulations suggest that these linkers do not

significantly occlude the pore but rather reduce the overall entrance section or, in some cases, form a “sieve” structure that permits ion and small molecule passage while restricting larger particles.

Thus, as a first approximation, we can consider $f_c \simeq f_e$.

Entry: Partially Adsorbing Boundary Condition – the Limiting Step

Entry into the nanopore is governed by a partially adsorbing boundary condition

$$-J(r_e) = k\, C(r_e), \tag{S5}$$

where k is the adsorption rate. The entry frequency is given by

$$fe = 2\pi C0\ k\ re2\ e\phi(re). \tag{S6}$$

The adsorption rate can be assumed to follow an Arrhenius-like relation:

$$k = k_0\, r_e\, \exp\left(-\frac{\Delta G}{k_B T}\right), \tag{S7}$$

where

- $\Delta G = \Delta G_0 - U$,
- ΔG_0 is the equilibrium free-energy barrier,
- U accounts for electric and advection effects that reduce (or increase) the barrier, and
- k_0 is a rate prefactor, approximated using an Eyring-like expression as $\frac{k_B T}{h}$.

Thus, the final expression for f_e becomes

$$f_e = 2\pi C_0\, r_e^3\, \frac{k_B T}{h}\, \exp\left[\phi(r_e) - \frac{\Delta G_0}{k_B T} + \frac{U}{k_B T}\right]. \tag{S8}$$

In the original model, it was assumed that $U = \phi_e k_B T$, so that

$$f_e = C\, \exp(-\Delta G_0)\ .$$

Since $C = C(\phi)$, and following the discussion on the approaching stage, we can consider that the proportionality constant remains nearly the same in the presence and absence of the entropic gate, with the main difference being in the equilibrium barrier ΔG_0.

Modeling ΔG_0: The Free Energy Barrier at Equilibrium

The capture model illustrated in the previous section was used in earlier work to compute the entrance free-energy barrier ΔG_0 for various proteins (including CRP and streptavidin) in $YaxA_{\Delta 40}B_{WT}$ (without the entropic gate) from experimentally measured capture rates.[1]

The estimated ΔG_0 was found to be lower than $20k_BT$ for CRP and smaller globular proteins (streptavidin, bovine thrombin, and hemoglobin).

Hence, the ability of the nanopore functionalized with disordered polypeptides to selectively permit or exclude proteins is determined by changes in the free-energy barrier,

$$\Delta G_0' = \Delta G_0 + \Delta G_0^* .$$

The additional barrier ΔG_0^* emerges from a combination of entropic and enthalpic contributions (electrostatic, hydrophilic/hydrophobic protein-protein interactions, hydrogen bonds, etc.), which collectively determine the probability of protein entry.

Regarding the entropic barrier formed by intrinsically disordered proteins in confined channels and cavities, several studies have attempted to theoretically quantify the equilibrium conformation of polymeric chains and the effects of electrostatic and hydrophobic interactions on the translocation potential[8,9], based on molecular theories that explicitly consider shape, size, conformation, charge and charge distribution, and intermolecular interactions. Interesting conclusions were drawn regarding the role of hydrophobicity and charge patterning in excluding or allowing the passage of specific proteins. However, these general considerations cannot be easily transferred from one system to another. Moreover, the morphological behavior of these systems is very complex and cannot be described by the simple theoretical arguments developed for infinite channels or planar walls.[10]

In general, the free-energy functional of the system, $G(h)$, is given by a combination of different terms:

$$G(h) = -T\,S_{solv}(h) - T\,S_{conf}(h) + H_{vdW}(h) + H_{ele}(h), \qquad \text{(S9)}$$

where

- h is the polymer extension,
- $T\,S_{solv}$ accounts for the mixing entropy of solvent molecules,
- $T\,S_{conf}$ accounts for the total conformational entropy of the chains,
- H_{vdW} accounts for the van der Waals interactions between polymer monomers, and

- H_{ele} accounts for electrostatic interactions between polymer monomers.

The forms of these terms can be highly nonlinear and environment dependent, leading to many different morphologies depending on the solvent quality, pore geometry, grafting position, and, in general, the degree of polymer confinement.

Overall, for hydrophobic chains (or under poor solvent conditions) the main regimes in which the polymer chains can be found are:

- collapsed onto the walls,
- forming a compact plug within the pore, or
- self-assembling into domains of various shapes due to microphase separation.

The morphology of these domains (aggregates on pore walls or stacked micelles along the pore axis) is mainly determined by the relationship between chain length and pore radius.

In our work, we functionalized the YaxAB pore with linkers of different lengths (*N* = 10–100 aa). The general observation is that increasing the linker length increases the entrance barrier, with the maximum effect observed at *N* = 70 aa (**Figure 5, Supporting Figure 26**). In agreement with the picture described above, our hypothesis is that the linkers generally reduce the entrance section (when aggregated on the pore walls) and that, beyond a certain length, they can form a "sealing state" (aggregated micelles) in which the linkers connect to form a mesh that prevents the passage of large molecules while still allowing electrolyte flow.

Emilsson et al.[9] derived a simple "de Gennes style" model[11] to estimate the free energy of polymer brush chains in functionalized solid-state nanopores as a function of polymer length, given the number of monomers and the pore radius *R*,

$$\frac{G_0(h)}{k_B T} = \frac{2\Gamma R \nu N^2}{2Rh - h^2} + \frac{3h^2}{2Nab} + \text{constant}\,, \qquad \text{(S10)}$$

where

- *h* is the polymer extension,
- *a* is the monomer length,
- *b* is the Kuhn length,
- *v* is the excluded volume parameter,
- *N* is the number of monomers, and

- Γ is the grafting density (roughly corresponding to the inverse of the area per coil).

The equilibrium height *H*, for a given *N* and *R*, corresponds to the minimum of *G*(*h*) with respect to *h*. As stated by the authors, expressions of this kind can accurately predict the brush height, although it is not straightforward to determine the most accurate values for *a*, *b*, and *v*.

Nevertheless, using reasonable parameter values, the expression was proven useful in predicting that a sealing state can occur when *h/R* > 0.8, and in roughly estimating the magnitude of the entropic barrier free energy. Calculations of Eq. (S10) with *a* = 0.3 nm, *b* = 1.0 nm, $\nu = \frac{4}{3}\pi a^3$, and Γ = 10/(4*πRa*) = 0.35nm^{-2}, for different values of *N*, are reported in Fig. 50a.

The formula predicts that, in our geometry, the sealing state would occur from *N* ≥ 50 (green line in panel a). Another interesting parameter that can be derived from their model is the comparison between the chain height in the confined state and that on a planar functionalized surface [9] (i.e., as *R* →∞),

$$H^* = \lim_{R\to\infty} H = \left[\frac{\Gamma ab\nu}{3}\right]^{\frac{1}{3}} N \ , \qquad \text{(S11)}$$

as reported in **Supporting Figure 50b**.

Here, we can see that for *N* ≤ 50 the entropic penalties due to excluded volume in confinement are essentially negligible, while they become quite significant for *N* ≥ 70.

Then, assuming that in the absence of any analyte inside the pore (i) the most favored state of our chains is the collapsed one and (ii) a metastable state can exist for the free chains, we can use Eq. (S10) to estimate an ideal barrier, ΔG_0^*,

$$\Delta G_0^* = G(H) - G(H^*)$$

required to extend the chains to their metastable, non-confined equilibrium length H^*. This barrier serves as a measure of the “work” needed to “disentangle” or “open” the polymer chains;

or, as an estimation of the probability of finding the pore open is $p_o \propto \exp(-\Delta G_0^*)$.

Although this additional calculation is rather symbolic, we observe an interesting difference between the *N* = 50 and *N* = 70 chains. In fact, while both are predicted to allow for a sealing state, the shorter chain exhibits a relatively low barrier, $\Delta G_0^* \simeq 1.7 k_B T$, corresponding to a spontaneous pore-opening probability of approximately $p_o \sim 20\%$. In contrast, the *N* = 70 case shows a much larger barrier, $\Delta G_0^* \simeq 21 k_B T$, so that the probability of the pore being open is

essentially zero. For the N = 100 case, the calculation cannot be performed since Eq. (S10) is only valid for h = 0–2R, and the computed ΔG_0^* becomes meaningless.

From this analysis, we conclude that for the N = 70 case the only way for a protein to enter the pore is to specifically interact with it—thus lowering the entry barrier by many k_BT. For example, in the case of Streptavidin binding to Strep-II tags, a very large binding free energy per binding site of 5.6kcal/mol ~ 9.4k_BT is observed.[2] Since Streptavidin presents four binding sites and the YaxAB complex exposes ten Strep-II tags, the entry barrier in this case would be effectively reduced to very low values, depending only on the probability of bond formation.

Finally, we also tried to compute the equilibrium length of the linkers by performing enhanced multiple-walker metadynamics MD simulations, starting from the equilibrated configurations shown in **Supporting Figure 32**. Here, the free-energy landscape is computed as a function of the average radius of gyration of the linkers, grouped into two sets, $R_{g,1}$ and $R_{g,2}$, normalized by the pore radius R_p = 5 nm (see **Supporting Figure 51**). Although the sampling is not particularly extensive in time or in the space of collective variables (due to the complexity and size of the system, 1.8M atoms), three main results can be confidently highlighted:

1. The intermediate cases (N = 50–70) exhibit a broad distribution of R_g values, exceeding the Emilsson[9] threshold of R_g/R_p = 0.8, indicating that they can reach the proper extension for forming a stable "sealing state".
2. The N = 100 case shows a net decrease in R_g compared to the N = 50–70 cases, suggesting that further increases in linker length do not enhance the probability of pore sealing.
3. In the N = 100 case, the long chains undergo a hydrophobic collapse together with their neighbors, forming large "hydrophobic blobs" (of three or four chains) that hardly extend toward chains on the opposite side.

In conclusion, both the theoretical arguments and the MD results strongly suggest that there exists a maximum in sealing propensity—and consequently a maximum in the entry barrier—for linker lengths in the range of N = 50–70 aa for the functionalized YaxAB pore, which is in excellent agreement with the experimental results.

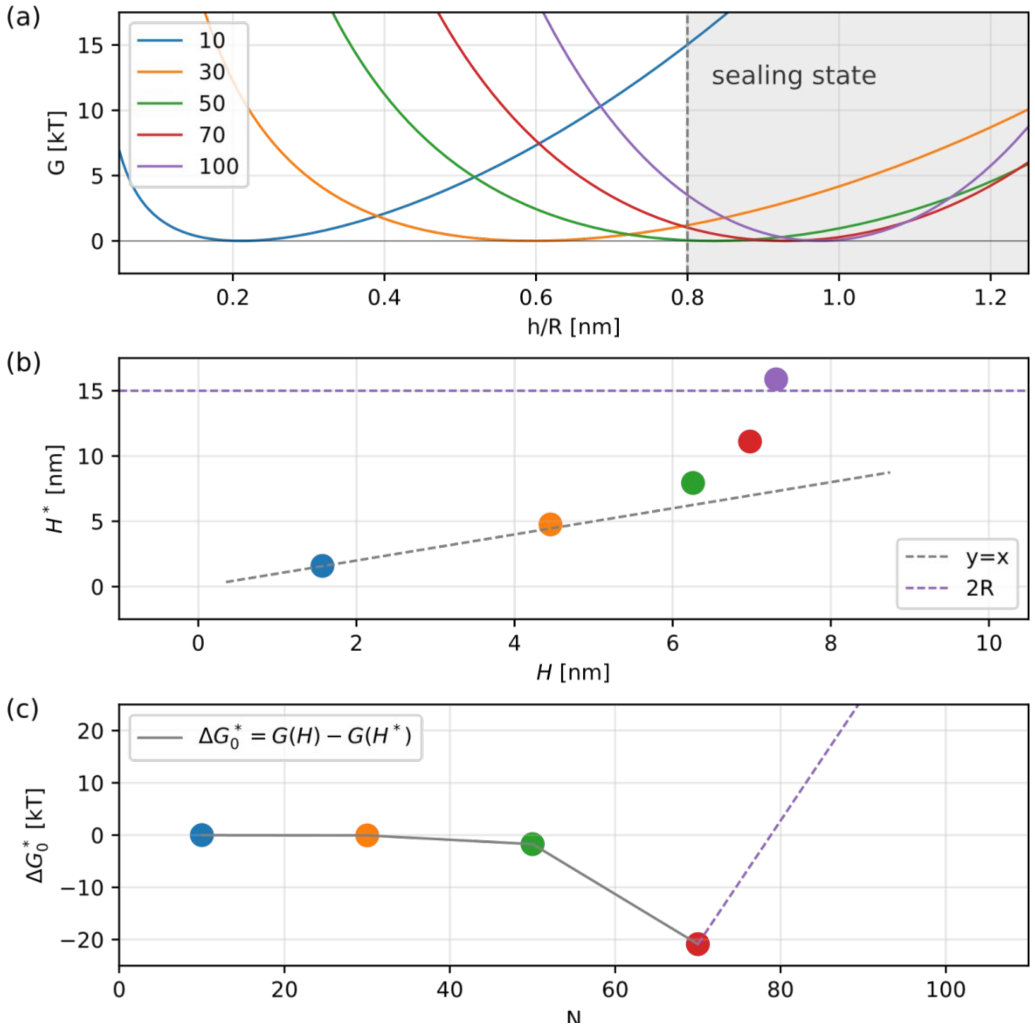

Supporting Figure 50: a) Calculated free energy $G_0(h)/k_BT$ as a function of polymer extension h, obtained from Eq. (S10), using parameters a = 0.3 nm, b = 1.0 nm, $\nu = \frac{4}{3}\pi a^3$, and Γ = 10/(4πRa) = 0.35nm^{-2}, for various chain lengths N. The curves illustrate that the sealing state is predicted to occur for $N \geq 50$. b) Comparison between the equilibrium height H of the polymer chains in confinement and the equilibrium height H^* on a planar surface (obtained in the limit $R \rightarrow \infty$). c) Ideal "opening" barrier, computed as $\Delta G_0^* = G(H) - G(H^*)$, indicating that the up to $N \leq 50$ the entropic penalty due to confinement can be considered negligible, while it becomes significant at $N \geq 70$. This quantity can be used to approximately quantify the probability to find the pore open, $p_o \propto \exp(-\Delta G_0*)$.

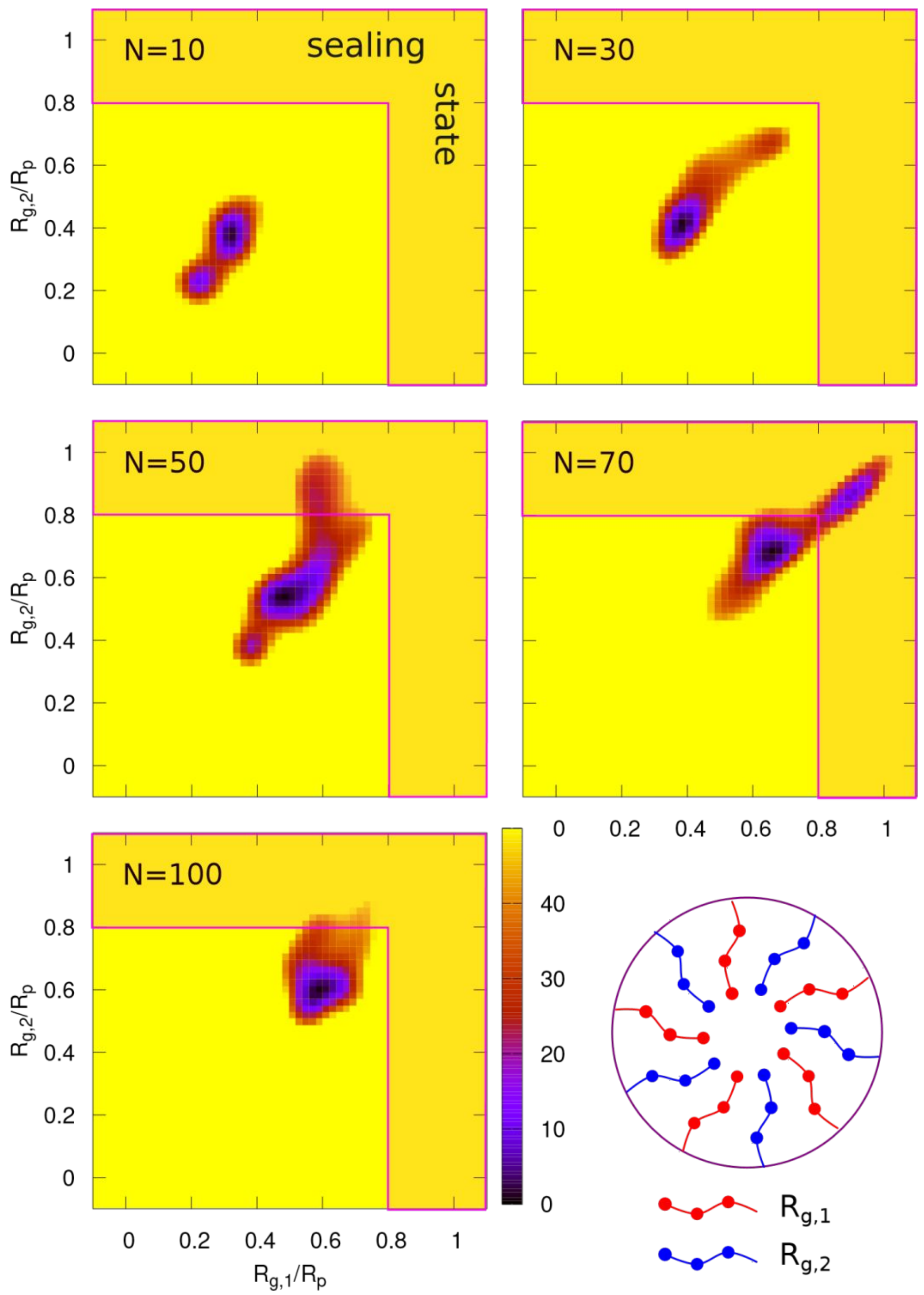

Supporting Figure 51: Free Energy Landscapes obtained from multiple-walker metadynamics simulations of the systems shown in **Supporting Figure 32**. Each panel corresponds to a different linker length N, where $R_{g,1}$ and $R_{g,2}$ i represent the mean radius of gyration for two distinct linker groups (depicted in blue and red in the bottom-right schematic). The inner pore radius is set to R_p = 50 ˚A. The color scale indicates the free energy, with darker regions corresponding to lower-energy states. For each system, three walkers were initialized from the last frame of the corresponding equilibrium simulations and sampled for 150 ns using well-tempered metadynamics with a bias factor of 16. The violet square area at R_g > 0.8 is the threshold reported by Emilsson et al.[9], above which a complete sealing state can be reached.

References

(1) Straathof, S.; Di Muccio, G.; Yelleswarapu, M.; Alzate Banguero, M.; Wloka, C.; Van Der Heide, N. J.; Chinappi, M.; Maglia, G. Protein Sizing with 15 Nm Conical Biological Nanopore YaxAB. *ACS Nano* **2023**, *17* (14), 13685–13699. https://doi.org/10.1021/acsnano.3c02847.

(2) Schmidt, T. G. M.; Koepke, J.; Frank, R.; Skerra, A. Molecular Interaction Between the Strep-Tag Affinity Peptide and Its Cognate Target, Streptavidin. *Journal of Molecular Biology* **1996**, *255* (5), 753–766. https://doi.org/10.1006/jmbi.1996.0061.

(3) Iyer, J. K.; Koh, C. Y.; Kazimirova, M.; Roller, L.; Jobichen, C.; Swaminathan, K.; Mizuguchi, J.; Iwanaga, S.; Nuttall, P. A.; Chan, M. Y.; Kini, R. M. Avathrin: A Novel Thrombin Inhibitor Derived from a Multicopy Precursor in the Salivary Glands of the Ixodid Tick, *Amblyomma Variegatum*. *FASEB j.* **2017**, *31* (7), 2981–2995. https://doi.org/10.1096/fj.201601216R.

(4) Shabane, P. S.; Izadi, S.; Onufriev, A. V. General Purpose Water Model Can Improve Atomistic Simulations of Intrinsically Disordered Proteins. *J. Chem. Theory Comput.* **2019**, *15* (4), 2620–2634. https://doi.org/10.1021/acs.jctc.8b01123.

(5) Rizzuti, B. Molecular Simulations of Proteins: From Simplified Physical Interactions to Complex Biological Phenomena. *Biochimica et Biophysica Acta (BBA) - Proteins and Proteomics* **2022**, *1870* (3), 140757. https://doi.org/10.1016/j.bbapap.2022.140757.

(6) Muthukumar, M. Theory of Capture Rate in Polymer Translocation. *The Journal of Chemical Physics* **2010**, *132* (19), 195101. https://doi.org/10.1063/1.3429882.

(7) Chinappi, M.; Yamaji, M.; Kawano, R.; Cecconi, F. Analytical Model for Particle Capture in Nanopores Elucidates Competition among Electrophoresis, Electroosmosis, and Dielectrophoresis. *ACS Nano* **2020**, *14* (11), 15816–15828. https://doi.org/10.1021/acsnano.0c06981.

(8) Tagliazucchi, M.; Peleg, O.; Kröger, M.; Rabin, Y.; Szleifer, I. Effect of Charge, Hydrophobicity, and Sequence of Nucleoporins on the Translocation of Model Particles through the Nuclear Pore Complex. *Proc. Natl. Acad. Sci. U.S.A.* **2013**, *110* (9), 3363–3368. https://doi.org/10.1073/pnas.1212909110.

(9) Emilsson, G.; Xiong, K.; Sakiyama, Y.; Malekian, B.; Ahlberg Gagnér, V.; Schoch, R. L.; Lim, R. Y. H.; Dahlin, A. B. Polymer Brushes in Solid-State Nanopores Form an Impenetrable Entropic Barrier for Proteins. *Nanoscale* **2018**, *10* (10), 4663–4669. https://doi.org/10.1039/C7NR09432A.

(10) Peleg, O.; Tagliazucchi, M.; Kröger, M.; Rabin, Y.; Szleifer, I. Morphology Control of Hairy Nanopores. *ACS Nano* **2011**, *5* (6), 4737–4747. https://doi.org/10.1021/nn200702u.

(11) de Gennes, P. G. Conformations of Polymers Attached to an Interface. *Macromolecules* **1980**, *13*, 1069–1075.
